# CXCL10 secreted by SPRY1-deficient epidermal keratinocytes fuels joint inflammation in psoriatic arthritis via CD14 signaling

**DOI:** 10.1172/JCI186135

**Published:** 2025-06-05

**Authors:** Fan Xu, Ying-Zhe Cui, Xing-Yu Yang, Yu-Xin Zheng, Xi-Bei Chen, Hao Zhou, Zhao-Yuan Wang, Yuan Zhou, Yi Lu, Ying-Ying Li, Li-Ran Ye, Ni-Chang Fu, Si-Qi Chen, Xue-Yan Chen, Min Zheng, Yong Yang, Xiao-Yong Man

**Affiliations:** 1 Department of Dermatology and; 2 Department of Orthopedic Surgery, Second Affiliated Hospital, Zhejiang University School of Medicine, Hangzhou, Zhejiang, China.; 3 Orthopedics Research Institute of Zhejiang University, Hangzhou, Zhejiang, China.; 4 Institute of Dermatology, Chinese Academy of Medical Sciences and Peking Union Medical College, Nanjing, Jiangsu, China.

**Keywords:** Dermatology, Immunology, Arthritis, Chemokines, Macrophages

## Abstract

Psoriatic arthritis (PsA) is a multifaceted, chronic inflammatory disease affecting the skin, joints, and entheses, and it is a major comorbidity of psoriasis. Most patients with PsA present with psoriasis before articular involvement; however, the molecular and cellular mechanisms underlying the link between cutaneous psoriasis and PsA are poorly understood. Here, we found that epidermis-specific SPRY1-deficient mice spontaneously developed PsA-like inflammation involving both the skin and joints. Excessive CXCL10 was secreted by SPRY1-deficient epidermal keratinocytes through enhanced activation of JAK1/2/STAT1 signaling, and CXCL10 blockade attenuated PsA-like inflammation. Of note, CXCL10 was found to bind to CD14, but not CXCR3, to promote the TNF-α production of periarticular CD14^hi^ macrophages via PI3K/AKT and NF-κB signaling pathways. Collectively, this study reveals that SPRY1 deficiency in the epidermis is sufficient to drive both skin and joint inflammation, and it identifies keratinocyte-derived CXCL10 and periarticular CD14^hi^ macrophages as critical links in the skin-joint crosstalk leading to PsA. This keratinocyte SPRY1/CXCL10/periarticular CD14^hi^ macrophage/TNF-α axis provides valuable insights into the mechanisms underlying the transition from psoriasis to PsA and suggests potential therapeutic targets for preventing this progression.

## Abstract 2

SPRY1-deficient keratinocytes produce CXCL10 to trigger arthritisCD14^hi^ macrophages exert pro-inflammatory effects in arthritis by producing TNF𝜶

## Graphical abstract

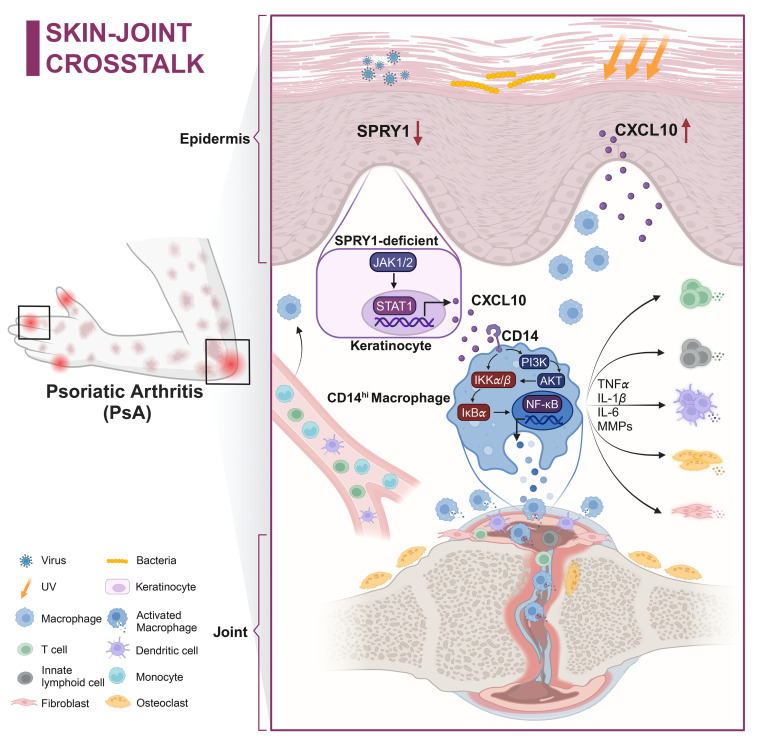

## Introduction

Psoriasis is a chronic, immune-mediated skin disease that affects approximately 125 million people worldwide, with erythematous and scaly plaques as its most common feature (1, 2). Psoriatic arthritis (PsA) is a multifaceted, chronic inflammatory disease affecting the skin, nails, joints, entheses, or spine, and is a major comorbidity of psoriasis (3, 4). PsA affects 0.10%–0.25% of the adult general population (5), and many patients develop a destructive form of arthritis with substantial morbidity and disability (4). Psoriasis usually precedes the development of PsA (6, 7), and up to 30% of patients with psoriasis will develop PsA (8). This sequential progression from skin lesions to arthritis suggests a crosstalk between skin and joints. However, the mechanisms underlying this progression remain unclear.

The epidermis, the outer layer of the skin, is primarily composed of keratinocytes, which play a central role in the recruitment and activation of immune cells by secreting soluble mediators in response to various stimuli (9, 10). The dermis exhibits substantial immune diversity, including resident and infiltrating immune cells such as T cells, macrophages, DCs, neutrophils, and mast cells (10, 11). Psoriatic skin lesions are triggered by the interaction between keratinocytes and immune cells, primarily involving the activation of IL-23/IL-17A and TNF-α pathways (12, 13). In contrast, the pathogenesis of PsA is not well understood due to its phenotypic heterogeneity involving multiple tissues (14), particularly the crosstalk between skin and joints. Notably, dermis and bone originate from a common primordial mesenchyme, and developmental pathways and epithelial-mesenchymal interactions in skin and bone share some remarkable similarities (15). Recent studies have shown that keratinocytes or skin-derived molecules can directly modulate bone remodeling by regulating the signaling pathways in osteoblasts, osteoclasts, and osteocytes, which mediate skin-bone crosstalk (16, 17). These findings highlight the importance of further investigation into skin-joint crosstalk to better understand the mechanisms underlying the transition from psoriasis to PsA.

Sprouty1 (SPRY1) is a known negative regulator of the RTK/Ras-MAPK signaling pathway (18). Our previous work has shown that SPRY1 is deficient in psoriatic epidermal keratinocytes and that overexpression of SPRY1 inhibits proliferation, enhances apoptosis, and alters differentiation in keratinocytes. Additionally, overexpression of SPRY1 in mice ameliorated imiquimod-induced psoriasis-like skin inflammation. These findings indicate that epidermal SPRY1 plays a crucial negative regulatory role in the pathogenesis of psoriasis (19, 20). However, the specific function of epidermal SPRY1 and the underlying mechanisms remain to be elucidated.

In this study, we generated epidermis-specific SPRY1-deficient (*Spry1*-cKO) mice and, surprisingly, observed that these mice spontaneously developed psoriasis-like skin inflammation and arthritis, closely resembling PsA. This finding indicates that alterations in the epidermis/skin alone are sufficient to drive joint inflammation. Focusing on the links between skin lesions and arthritis, we utilized these *Spry1*-cKO mice as a mouse model for PsA and found that SPRY1-deficient keratinocytes produced excessive CXCL10, which bound to CD14 to promote the proinflammatory response of periarticular CD14^hi^ macrophages. This study identifies keratinocyte-derived CXCL10 and periarticular CD14^hi^ macrophages as critical links in the skin-joint crosstalk leading to PsA and provides insights into the mechanisms underlying the transition from psoriasis to PsA.

## Results

### Epidermis-specific SPRY1-deficient mice spontaneously develop psoriasis-like skin lesions and arthritis.

**Figure 1:**
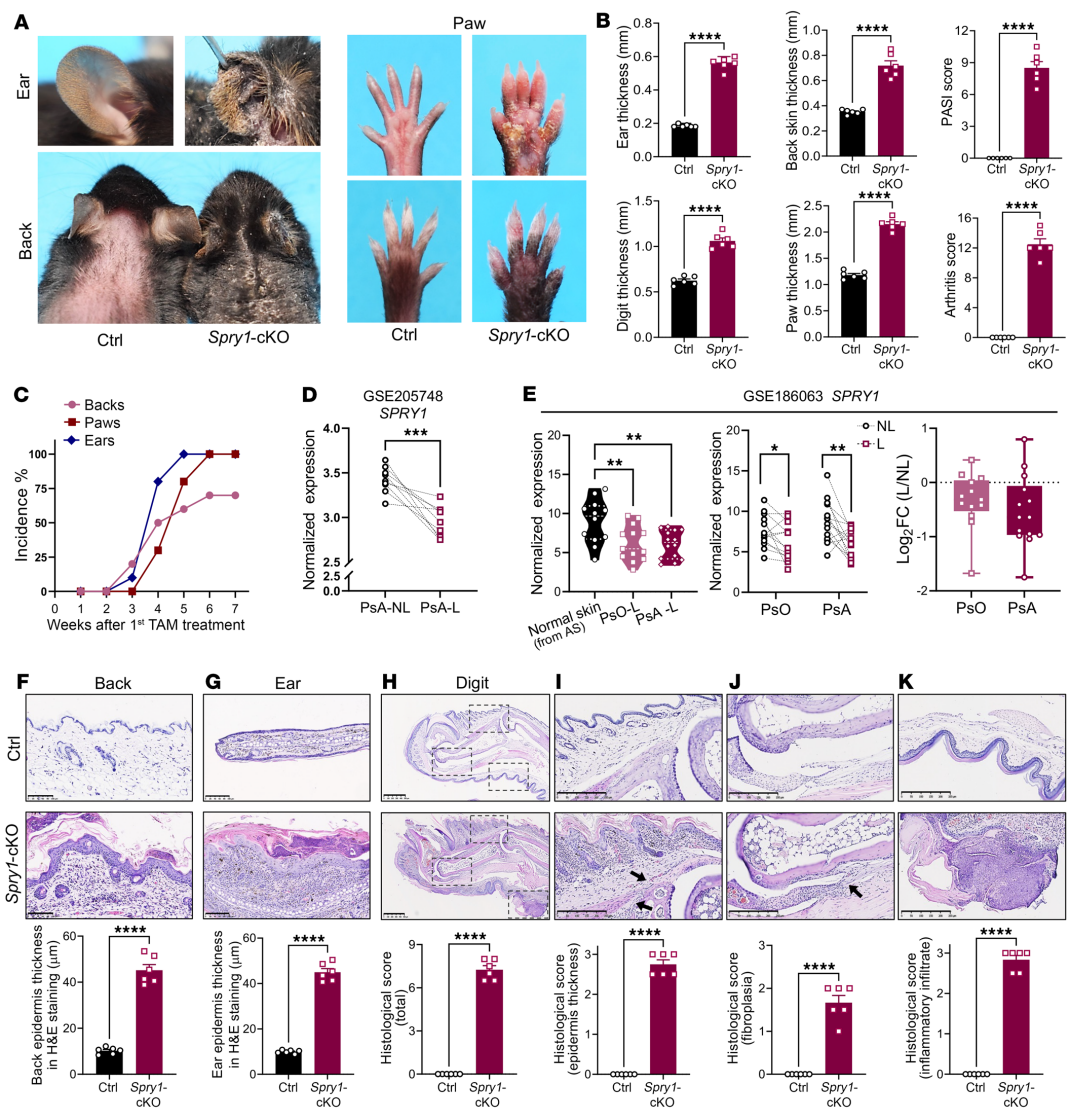
Epidermis-specific SPRY1-deficient mice spontaneously develop psoriasis-like skin lesions and arthritis. (**A**) Macroscopic views of the ears, shaved back skin, and paws from control and *Spry1*-cKO mice. (**B**) Ear and back skin thickness; PASI scores of back skin; digit, and paw thickness; and arthritis scores of control and *Spry1*-cKO mice (*n* = 6). (**C**) Incidence of inflammation in ears, back skin, and paws of control and *Spry1*-cKO mice after tamoxifen treatment (*n* = 20). (**D**) *SPRY1* gene expression in lesional and nonlesional skin from patients with PsA from the NCBI’s GEO database (GSE205748). (**E**) *SPRY1* gene expression in lesional and nonlesional skin from patients with psoriasis or PsA, and normal skin from patients with ankylosing spondylitis from the GEO database (GSE186063). (**F** and **G**) Representative H&E staining of the back skin and ears of control and *Spry1*-cKO mice. Lower panels show quantification of epidermis thickness respectively (*n* = 6). Scale bar: 100 μm. (**H**–**K**) Representative H&E staining of the digits from paws of control and *Spry1*-cKO mice (**H**), scale bar: 500 μm. Boxed areas magnified in (**I**–**K**) are the following: epidermal hyperplasia (**I**), fibroplasia (**J**), and inflammatory infiltrate (**K**), black arrows indicate enthesitis, scale bar: 250 μm. Lower panels show histological scores (*n* = 6). Data are shown as mean ± SEM. *P* values were determined using 2-tailed unpaired Student’s *t* test (**B**, the right panel of **E**, and **F**–**K**), 2-tailed paired Student’s *t* test (**D** and the middle panel of **E**), and 1-way ANOVA (the left panel of **E**). *P < 0.05, **P < 0.01, ***P < 0.001, ****P < 0.0001.

First, we confirmed that SPRY1 was specifically downregulated in the lesional skin of psoriasis but not in atopic dermatitis, another inflammatory skin disease (Supplemental Figure 1, A and B; supplemental material available online with this article; https://doi.org/10.1172/JCI186135DS1). To gain a better understanding of how epidermal SPRY1 negatively regulates the development of psoriasis, we generated *K14^CreERT^Spry1^fl/fl^* mice by crossing mice with loxP-flanked *Spry1* alleles (*Spry1^fl/fl^*) with keratin 14-Cre/ERT (*K14-CreERT*) transgenic mice. Tamoxifen was used to induce Cre-mediated recombination and *Spry1* deletion in keratinocytes. At 3 weeks of age, tamoxifen was administered to *K14^CreERT^Spry1^fl/fl^* (referred to as *Spry1*-cKO) and *Spry1^fl/fl^* (referred to as control/ctrl) mice for 5 consecutive weeks (4 days per week, 100 mg/kg body weight per day). The epidermis-specific deletion of SPRY1 was confirmed in *Spry1*-cKO mice (Supplemental Figure 1, C–F). Interestingly, 3 weeks after the first tamoxifen induction, some *Spry1*-cKO mice developed diffuse erythematous and scaly plaques on the back and ears (closely resembling the skin lesions of human psoriasis). However, they did not exhibit any signs of arthritis. Four weeks after the first tamoxifen induction, scaly skin irritations and symmetrical swelling of the digits in the paws were observed, strongly reminiscent of PsA. Additionally, more mice exhibited a psoriasis-like skin phenotype on the back and ears. Six weeks after the first tamoxifen induction, almost 100% of *Spry1*-cKO mice developed arthritis in paws (dactylitis) and psoriasis-like skin inflammation on their ears and paws, while 70% of *Spry1*-cKO mice showed psoriasis-like dermatitis on the back (Figure 1, A–C, and Supplemental Figure 1G). Enlarged skin-draining lymph nodes and popliteal lymph nodes were also observed in *Spry1*-cKO mice (Supplemental Figure 1H).

To determine whether SPRY1 expression in the skin is more strongly correlated with PsA than with psoriasis, we analyzed *SPRY1* gene expression using GSE205748 and GSE186063 in the NCBI’s Gene Expression Omnibus (GEO) in lesional and nonlesional skin from patients with psoriasis or PsA, as well as in normal skin from patients with ankylosing spondylitis (21, 22). We found that the expression of the *SPRY1* gene was more significantly downregulated in the lesional skin of PsA (Figure 1, D and E).

The histology of the back skin, ears, and digits and nails of paws from *Spry1*-cKO mice showed typical features of psoriasis and PsA, such as epidermal hyperplasia, hyperkeratosis with parakeratosis, microabscesses accumulated on the surface of the thickened epidermis, increased and dilated blood vessels, extensive inflammatory infiltration in the dermis and periarticular areas, fibroplasia, enthesitis, and inflammatory infiltration beneath the nail matrix, nail bed, and hyponychium (Figure 1, F–K, and Supplemental Figure 1, I and J). Moreover, the keratinocytes from *Spry1*-cKO mice exhibited an increased proportion of Ki67 positivity and a decreased proportion of apoptosis, suggesting epidermal hyperproliferation and impaired apoptosis (Supplemental Figure 1, K–N).

**Figure 2:**
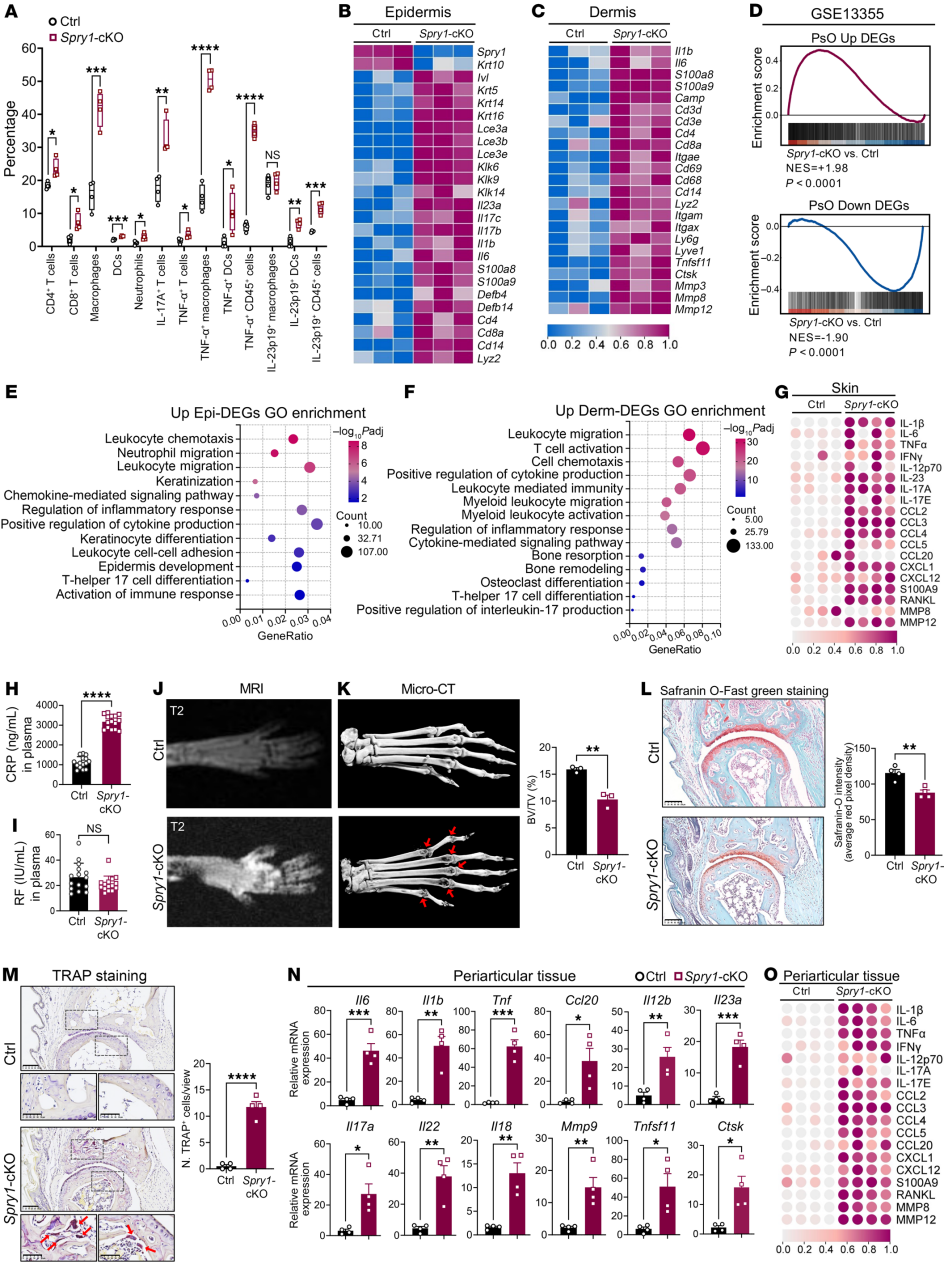
Skin and joint inflammation in *Spry1*-cKO mice exhibit hallmarks of psoriasis and psoriatic arthritis. (**A**) Percentages of immune cell subsets in ear skin from control and *Spry1*-cKO mice determined by flow cytometry (*n* = 4). (**B** and **C**) Heatmap of selected genes based on RNA-Seq data from epidermis (**B**) and dermis (**C**) of control and *Spry1*-cKO mice (adjusted *P* < 0.05 and |log_2_FC| > 0.5, *n* = 3). (**D**) GSEA of upregulated or downregulated DEGs in human psoriatic skin (GSE13355) on genes of mouse skin (epidermis plus dermis) ranked by log_2_FC between *Spry1*-cKO and control mice. (**E** and **F**) GO pathway enrichment of upregulated DEGs (*Spry1*-cKO vs. control) in epidermis (**E**) and dermis (**F**) (adjusted *P* < 0.05). (**G**) Heatmap of selected cytokines in ear skin from control and *Spry1*-cKO mice detected by Luminex assays (*n* = 4). (**H** and **I**) ELISA quantification of CRP (**H**) and rheumatoid factor (RF) (**I**) in plasma from control and *Spry1*-cKO mice (*n* = 15). (**J** and **K**) T2-weighted MRI (**J**) and micro-CT (**K**) images of paws from control and *Spry1*-cKO mice. Red arrows indicate bone erosions; right, quantification of bone volume/total volume (BV/TV) in erosion areas (*n* = 3). (**L**) Safranin O-Fast green staining of articular cartilage in paws of control and *Spry1*-cKO mice. Scale bar: 100 μm. Right, quantification of Safranin-O intensity (*n* = 4). (**M**) TRAP staining of osteoclasts in interphalangeal joints of paws from control and *Spry1*-cKO mice (scale bar: 100 μm); boxed areas are magnified below (scale bar: 50 μm); red arrows indicate TRAP^+^ osteoclasts. Right, quantification of TRAP^+^ osteoclasts (*n* = 4). (**N**) Relative mRNA expression of genes associated with PsA in periarticular tissue from control and *Spry1*-cKO mice (*n* = 4). (**O**) Heatmap of selected cytokines in periarticular tissue from control and *Spry1*-cKO mice detected by Luminex assays (*n* = 4). Data are shown as mean ± SEM. *P* values determined using 2-tailed unpaired Student’s *t* test (**A**, **H**, **I**, and **K**–**N**). *P < 0.05, **P < 0.01, ***P < 0.001, ****P < 0.0001.

Next, we investigated immune infiltration in the skin of mouse ears by flow cytometry. The percentages of CD4^+^ T cells, CD8^+^ T cells, IL-17A^+^ T cells, F4/80^+^ macrophages, CD11c^+^ F4/80^–^ DCs, CD11b^+^ Ly6G^+^ neutrophils, TNF-α^+^ macrophages or DCs or total CD45^+^ cells, and IL-23p19^+^ DCs or total CD45^+^ cells were all increased in the ear skin of *Spry1*-cKO mice (Figure 2A and Supplemental Figure 2A). Consistent results were also observed in immunofluorescence staining of these immune cell markers (Supplemental Figure 2B). In addition, the epidermis of *Spry1*-cKO mice exhibited abnormal epidermal differentiation and epidermal barrier dysfunction as evidenced by the upregulation of K14 and downregulation of filaggrin and loricrin, and the expression of the endothelial marker CD31 and the antimicrobial peptide cathelicidin (LL-37) was upregulated in the skin of *Spry1*-cKO mice (Supplemental Figure 2B).

We then performed bulk RNA-Seq of the epidermis and dermis separately from *Spry1*-cKO and control mice. Principal component analysis showed distinct gene expression patterns among the 4 groups: *Spry1*-cKO epidermis, *Spry1*-cKO dermis, control epidermis, and control dermis (Supplemental Figure 2C). Among all differentially expressed genes (DEGs) in the epidermis and dermis (*Spry1*-cKO vs. control, adjusted *P* < 0.05 and |log_2_FC| > 0.5), we observed significant similarities in hallmark profiles of psoriasis-related gene expression patterns between skin lesions of *Spry1*-cKO mice and patients with psoriasis (Figure 2, B and C). Specifically, the deletion of *Spry1* in the epidermis of *Spry1*-cKO mice was confirmed again, and these epidermal/dermal DEGs were associated with epidermal differentiation and epidermal barrier (*Ivl, K10, K14, Lce3a, Lce3b*, etc.), antimicrobial defense (*S100a8, S100a9, Camp*, etc.), inflammation and immune infiltration (*Klk6, Il23a, Il17b, Il17c, Il1b, Il6, Cd4, Cd8a, Cd14, Cd68, Itgax, Ly6g*, etc.) (23). Notably, some dermal DEGs (*Tnfsf11, Ctsk, Mmp3, Mmp8*, etc.) were associated with osteoclast differentiation and activation, which is linked to arthritis (24). Moreover, Gene Set Enrichment Analysis (GSEA) showed significant overlap in the transcriptional signatures of skin lesions between *Spry1*-cKO mice (epidermis plus dermis) and 58 patients with psoriasis (GEO GSE13355) (25) (Figure 2D). The Gene Ontology pathway enrichment analysis of upregulated epidermal and dermal DEGs highlighted that the pathways involved in the development of psoriasis and PsA were activated in *Spry1*-cKO skin lesions, including keratinization, Th17 cell differentiation, leukocyte chemotaxis, cytokine production, cytokine-mediated signaling pathways, bone resorption, and osteoclast differentiation (Figure 2, E and F). The Luminex assays confirmed that proinflammatory cytokines involved in the pathogenesis of psoriasis and PsA, including IL-1β, TNF-α, IL-23, IL-17A, CCL2, CCL3, CXCL1, S100A9, RANKL, and MMP12, were highly elevated in *Spry1*-cKO skin (Figure 2G and Supplemental Figure 2D).

To distinguish the similarity of the *Spry1*-cKO joint phenotype with PsA from rheumatoid arthritis, we examined C-reactive protein (CRP) and rheumatoid factor levels in *Spry1*-cKO mice plasma. As expected, the plasma CRP level was highly elevated, while the rheumatoid factor level remained unaltered (Figure 2, H and I). Furthermore, the paws of *Spry1*-cKO mice showed increased expression of K14 and CD45 (Supplemental Figure 3, A and B). MRI and micro-computed tomography (micro-CT) showed evident soft tissue swelling and bone erosions in the paws of *Spry1*-cKO mice (Figure 2, J and K). Additionally, cervicothoracic spinal curvature (kyphosis) and reduced bone density (indicating bone erosions) in the sacroiliac joints and pubic symphysis developed in the *Spry1*-cKO mice (Supplemental Figure 3, C–E). Safranin O-Fast green staining and tartrate-resistant acid phosphatase (TRAP) staining showed a decrease in Safranin O-Fast intensity and an increase in TRAP^+^ osteoclast numbers in the interphalangeal joints of the paws of *Spry1*-cKO mice, indicating cartilage damage and bone erosions (Figure 2, L and M). To further identify psoriatic and arthritic characteristics in paws of *Spry1*-cKO mice, we isolated periarticular tissue, analyzed the expression of relevant proinflammatory cytokines, and found that these proinflammatory cytokines were significantly upregulated (Figure 2, N and O, and Supplemental Figure 3F). However, because of the limited detection range of the Luminex assays, only some of these cytokines were detected and found to be elevated in the plasma of *Spry1*-cKO mice (Supplemental Figure 3G). Collectively, these phenotypical, histological, immunological, and molecular changes observed in the skin and joints of *Spry1*-cKO mice exhibited hallmarks of psoriasis and PsA. Moreover, SPRY1 deficiency in the epidermis was sufficient to drive both skin and joint inflammation, suggesting that SPRY1 plays a critical role in skin-joint crosstalk during the development of PsA.

### Excessive CXCL10 secreted by SPRY1-deficient keratinocytes promotes PsA-like inflammation.

Given that epidermal SPRY1 deficiency is strongly linked to PsA (Figure 1E), and that the typical PsA-like phenotype (skin and joint inflammation of the paws) was 100% prevalent in *Spry1*-cKO mice (Figure 1C), we next focused on dissecting the underlying mechanisms of skin-joint crosstalk in the pathogenesis of PsA.

**Figure 3:**
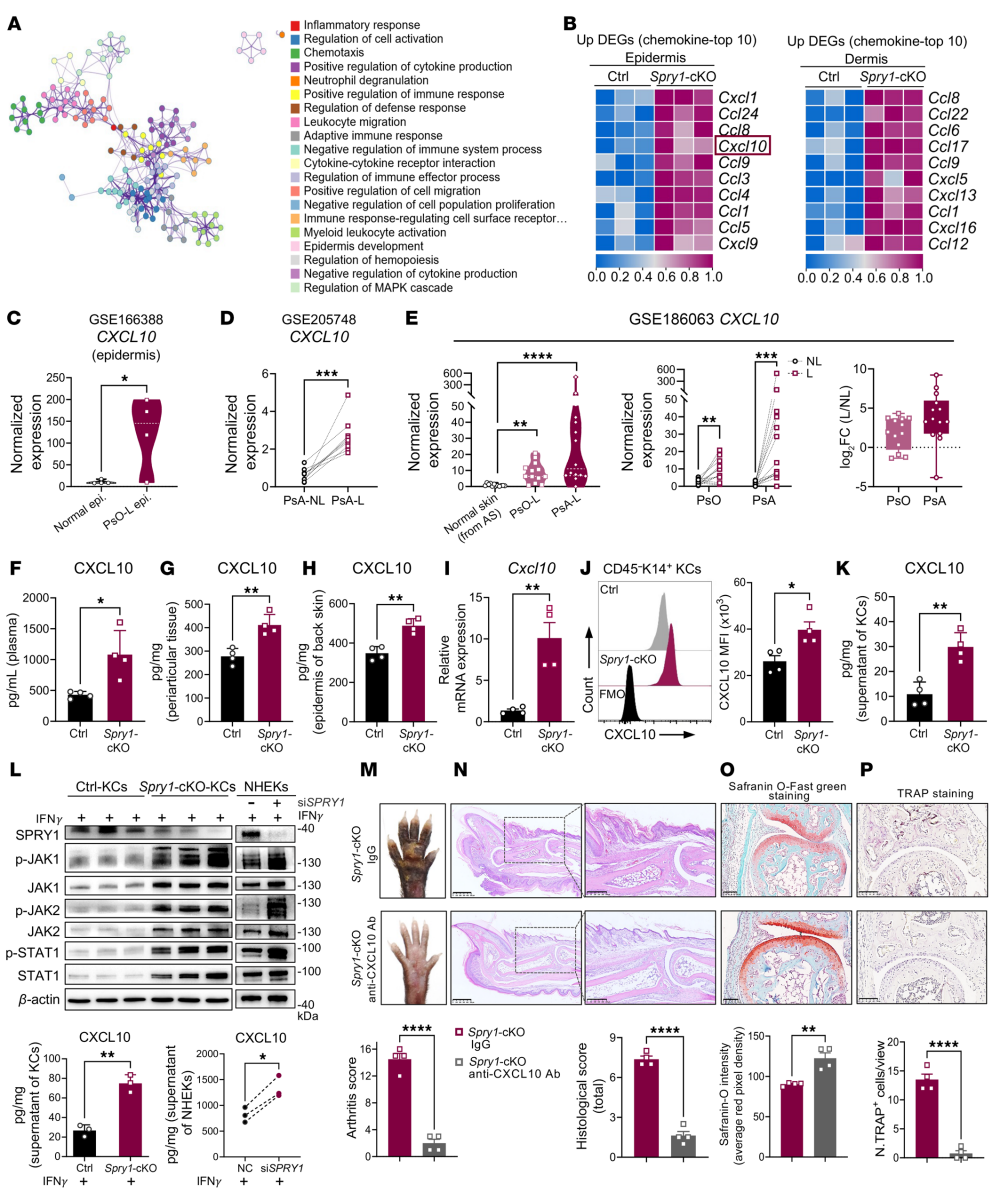
Excessive CXCL10 secreted by SPRY1-deficient keratinocytes promotes psoriatic arthritis–like inflammation. (**A**) Network of representative terms of all epidermal DEGs and all dermal DEGs (*Spry1*-cKO vs. control) commonly enriched by Metascape (*P* < 0.01). (**B**) Heatmap of top 10 upregulated chemokine genes in epidermis and dermis of *Spry1*-cKO mice (adjusted *P* < 0.05 and |log_2_FC| > 0.5, *n* = 3). (**C**–**E**) *CXCL10* gene expression in human datasets: normal vs lesional epidermis (psoriasis, GSE166388); lesional vs nonlesional skin (PsA, GSE205748); and comparison across psoriasis, PsA, and ankylosing spondylitis (GSE186063). (**F**–**H**) ELISA quantification of CXCL10 in plasma, periarticular tissue, and epidermis of back skin from control and *Spry1*-cKO mice (*n* = 4). (**I**) Relative mRNA expression of *Cxcl10* in mouse keratinocytes (*n* = 4). (**J**) Flow cytometric histograms and quantification of CXCL10 MFI of CD45^–^K14^+^ mouse keratinocytes (*n* = 4). (**K**) ELISA quantification of CXCL10 in supernatant of mouse keratinocytes (*n* = 4). (**L**) Immunoblotting analysis of protein levels associated with JAK1/2/STAT1 pathway in keratinocytes (left) or NHEKs transfected with siSPRY1 (right), both treated with 50 ng/mL recombinant IFN-γ for 24 hours (*n* = 3). ELISA quantification of *CXCL10* in the supernatant of keratinocytes (*n* = 3). (**M**–**P**) Representative macroscopic views (**M**), H&E staining (scale bar: left, 500 μm; right, 250 μm) (**N**), Safranin O-Fast green staining (scale bar: 100 μm) (**O**), and TRAP staining (scale bar: 100 μm) (**P**) of paws from age- and sex-matched *Spry1*-cKO mice treated with anti-CXCL10 antibody and isotype antibody (IgG). Quantification shown below (*n* = 4). Data are shown as mean ± SEM. *P* values determined using 2-tailed unpaired Student’s *t* test (**C**, right panel of **E** and **F**–**K**, lower left panel of **L**, and **M**–**P**), 2-tailed paired Student’s *t* test (**D**, middle panel of **E**, lower right panel of **L**), and 1-way ANOVA (left panel of **E**). *P < 0.05, **P < 0.01, ***P < 0.001, ****P < 0.0001.

Considering the loss of epidermal SPRY1 as an initiation factor, we identified the most significantly activated pathways enriched in both epidermis and dermis of *Spry1*-cKO mice by Metascape (Figure 3A and Supplemental Figure 4A). Chemotaxis and cell migration were identified as important pathways that could bridge the crosstalk between skin and joints (Figure 3A). Therefore, we listed the top 10 upregulated chemokines in the epidermis or dermis of *Spry1*-cKO mice (Figure 3B). Notably, the expression of *CXCL10*, also known as IFN-γ–induced protein (IP-10), was highly upregulated only in the epidermis of *Spry1*-cKO mice. CXCL10 has been found elevated in the serum and synovial fluid of patients with PsA, and it is known to predict the future development of PsA in patients with psoriasis (26–29). However, the cellular and molecular mechanisms by which CXCL10 contributes to the pathogenesis of PsA, especially the transition from psoriasis to PsA, remain incompletely understood. We ascertained that CXCL10 levels were elevated in the serum and lesional epidermis of patients with psoriasis (GEO GSE166388) (30) (Figure 3C and Supplemental Figure 4, B and C), and *CXCL10* was more significantly upregulated in the lesional skin of PsA compared with psoriasis (GEO GSE205748 and GSE186063) (Figure 3, D and E). Meanwhile, it was confirmed that CXCL10 was elevated in the plasma, periarticular tissue, and epidermis of *Spry1*-cKO mice (Figure 3, F–H), with a notable increase in keratinocytes (Figure 3, I–K, and Supplemental Figure 4D). Importantly, we further confirmed that keratinocytes were the dominant source of CXCL10 in the skin of *Spry1*-cKO mice (Supplemental Figure 4E), as it can also be expressed in other cells, such as T cells, neutrophils, monocytes, macrophages, endothelial cells, and fibroblasts (31).

CXCL10 secretion is primarily mediated by the IFN-γ–induced activation of the JAK 1/2/STAT1 signaling pathway (32). SPRY1 is a well-known negative regulator of RTK/Ras-ERK pathway signaling (18) and has been reported to inhibit JAK2 (33). To determine whether SPRY1 can regulate CXCL10 secretion in keratinocytes through the JAK1/2/STAT1 signaling pathway, we examined protein levels of phosphorylated JAK1 (p-JAK1), JAK1, phosphorylated-JAK2 (p-JAK2), JAK2, phosphorylated-STAT1 (p-STAT1), and STAT1 in primary cultured epidermal keratinocytes from control and *Spry1*-cKO mice, and in primary cultured normal human epidermal keratinocytes treated with or without si*SPRY1* under IFN-γ stimulation. As expected, the activation of the JAK1/2/STAT1 signaling pathway was significantly enhanced, resulting in elevated CXCL10 secretion in SPRY1-deficient keratinocytes (Figure 3L), and after inhibition of the JAK1/2/STAT1 signaling pathway with JAK inhibitors tofacitinib and upadacitinib, the elevated secretion of CXCL10 was reversed (Supplemental Figure 4F).

To further validate the pathogenic effects of CXCL10, we injected CXCL10-neutralizing antibody into the peritoneal cavity and paws of *Spry1*-cKO mice during tamoxifen induction. Blocking CXCL10 rescued PsA-like phenotypes (skin and joint inflammation), as indicated by attenuated epidermal hyperplasia, inflammatory infiltration, enthesitis, cartilage damage, and bone erosions (Figure 3, M–P, and Supplemental Figure 4, G-K). On the other hand, CXCL1 was the top 1 upregulated chemokine in the epidermal transcriptome of *Spry1*-cKO mice (Figure 3B), known to potently recruit neutrophils (34). Consistently, CXCL1 was elevated in the plasma, periarticular tissue, and ears of *Spry1*-cKO mice (Supplemental Figure 5A). However, CXCL1 blockade did not significantly attenuate PsA-like inflammation, although a slight reduction in scaling was observed (Supplemental Figure 5, B and C).

Taken together, these results indicate that excessive CXCL10 secreted by SPRY1-deficient keratinocytes through enhanced activation of JAK1/2/STAT1 signaling is crucial for the development of PsA-like inflammation in *Spry1*-cKO mice.

### The pathogenic role of keratinocyte-derived CXCL10 in PsA-like inflammation is independent of CXCR3 and TLR4.

**Figure 4:**
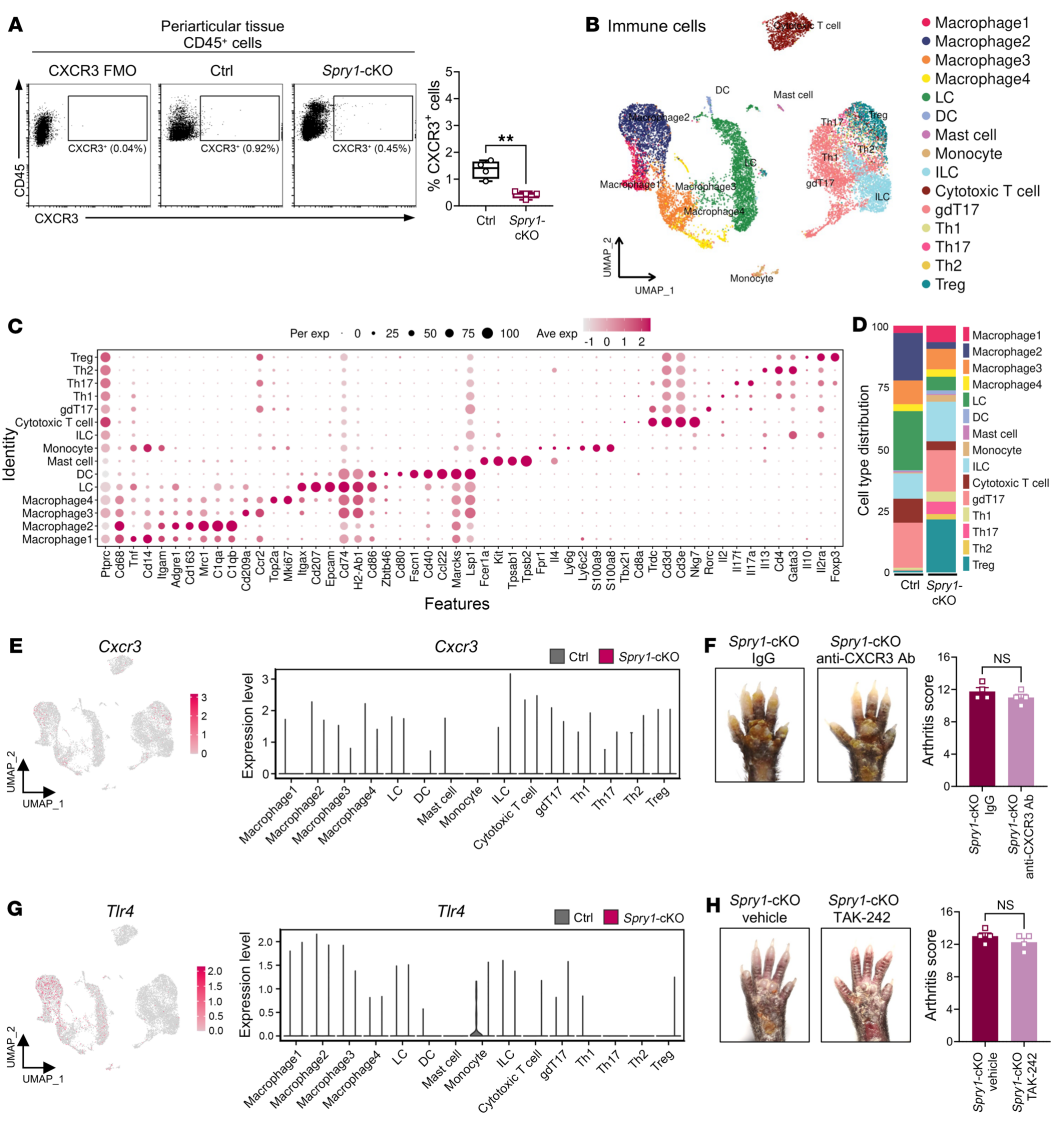
The pathogenic role of keratinocyte-derived CXCL10 in psoriatic arthritis–like inflammation is independent of CXCR3 and TLR4. (**A**) Flow cytometric plots (left) and percentages (right) of CD45^+^CXCR3^+^ cells in the periarticular tissue of control and *Spry1*-cKO mice (*n* = 4). (**B**) UMAP plots of immune cells from the periarticular tissue of control and *Spry1*-cKO mice by scRNA-Seq, showing 15 clusters (*n* = 3). (**C**) Dot plots showing the scaled expression of selected marker genes for all immune cell types defined in **B**. (**D**) Bar plots showing the distribution of all immune cell types. (**E**) UMAP plots (left) and violin plots (right) showing *Cxcr3* expression in all immune cell types. (**F**) Representative macroscopic views (left) and arthritis scores (right) of the paws from age- and sex-matched *Spry1*-cKO mice treated with anti-CXCR3 antibody and isotype antibody (IgG) (*n* = 4). (**G**) UMAP plots (left) and violin plots (right) showing *Tlr4* expression in all immune cell types. (**H**) Representative macroscopic views (left) and arthritis scores (right) of the paws from age- and sex-matched *Spry1*-cKO mice treated with TAK-242 (an inhibitor of TLR4 signaling) and vehicle (*n* = 4). Data are shown as mean ± SEM. *P* values were determined using 2-tailed unpaired Student’s *t* test (**A**, **F**, and **H**). ** *P* < 0.01.

The target immune cells of CXCL10 were examined using flow cytometry. However, the population of CXCR3-positive cells, which is the classic receptor of CXCL10, was limited in the periarticular tissue of *Spry1*-cKO mice, even less than that of control mice (Figure 4A).

To further investigate the periarticular immune microenvironment in paws, single-cell RNA-Seq (scRNA-Seq) of the periarticular tissue from *Spry1*-cKO and control mice was performed. To obtain enough immune cells for analysis, we pooled 4 mice to create 1 sample for the control group (12 control mice in total for *n* = 3). We then sorted CD45^+^ and CD45^–^ cells and mixed them at a ratio of 3:1 to enrich immune cells, and then generated single-cell transcriptomic profiles using 10x Genomics on the Illumina platform (Supplemental Figure 6, A and B). A total of 37,524 cells were detected, with an average of 74,396 reads and 1,638 genes per cell. After quality control, 24,616 cells were retained for subsequent analysis. The *Spry1*-cKO group had a mean of 3,387 cells per mouse, and the control group had a mean of 4,818 cells per mouse.

Unbiased clustering analysis identified multiple clusters of periarticular cells from *Spry1*-cKO mice, including Langerhans cells (*Cd207, Epcam*, and *Itgax*), DCs (*Fscn1, Lsp1*, and *Cd40*), macrophages (*Cd68, Cd163*, and *Mrc1*), monocytes (*Ly6c2* and *Cd14*), T cells (*Cd3e* and *Cd3d*), smooth muscle cells (*Acta2*), endothelial cells (*Vwf* and *Pecam1*), keratinocytes (*Krt5* and *Kr15*), and fibroblasts (*Pdgrfra* and *Col1a2*) (Supplemental Figure 6, C and D). The subclustering of *Prprtc*^+^ immune cells revealed heterogeneous subsets with distinct characteristics, such as Treg, Th2, Th17, Th1, and γδT17 (gdT17) in T cells (Figure 4, B–D). Consistently, *Cxcr3* expression at single-cell resolution remained at a relatively low level in periarticular immune cells from both *Spry1*-cKO and control mice (Figure 4E). Furthermore, blocking CXCR3 with its neutralizing antibody did not improve the PsA-like inflammation in *Spry1*-cKO mice, indicating that CXCR3 has limited involvement in this pathological process (Figure 4F and Supplemental Figure 6E).

Several studies have reported that TLR4 can act as a receptor for CXCL10 in specific cells with distinct biological functions, such as pancreatic β-cells, CD4^+^ T cells, and alveolar macrophages (35–37). Therefore, we examined the expression and function of TLR4 in the mouse periarticular tissue and found that *Tlr4* expression was slightly lower in macrophages and monocytes of *Spry1*-cKO mice compared with the control group (Figure 4G). Inhibition of TLR4 with TAK-242 did not ameliorate the PsA-like inflammation in *Spry1*-cKO mice (Figure 4H and Supplemental Figure 6F). These results suggest that the pathogenic role of keratinocyte-derived CXCL10 in PsA-like inflammation is independent of CXCR3 and TLR4.

### TNF-α is a key pathogenic downstream mediator in PsA-like inflammation.

In light of the findings above, the cellular and molecular mechanisms underlying the development of PsA-like inflammation in *Spry1*-cKO mice downstream of CXCL10 need to be further elucidated. TNF-α inhibitors, including adalimumab, infliximab, and etanercept, have been widely used to treat PsA for their remarkable efficacy in improving skin and musculoskeletal symptoms (3, 38). Therefore, TNF-α can be regarded as a crucial downstream effector cytokine in PsA (39).

We next analyzed the expression of *Tnf* from scRNA-Seq data. *Tnf* was abundantly expressed in macrophages, Langerhans cells, monocytes, and Th1 cells. Macrophages were the primary source of *Tnf* in the periarticular tissue of *Spry1*-cKO mice, with higher levels compared with control mice (Supplemental Figure 7A). To confirm the key pathogenic role of TNF-α, a TNF-α–neutralizing antibody was injected into the peritoneal cavity and paws of *Spry1*-cKO mice during tamoxifen induction. Notably, inhibition of TNF-α resulted in a dramatic remission of PsA-like inflammation in the skin and joints (Supplemental Figure 7, B–I). More importantly, TNF-α levels were significantly downregulated in the plasma, periarticular tissue, and ears of *Spry1*-cKO mice after CXCL10 blockade (Supplemental Figure 7J). Taken together, these results indicate that TNF-α is a key pathogenic mediator downstream of CXCL10 in PsA-like inflammation of *Spry1*-cKO mice.

### Periarticular CD14^hi^ macrophages play a predominant proinflammatory role in the development of PsA-like inflammation.

**Figure 5:**
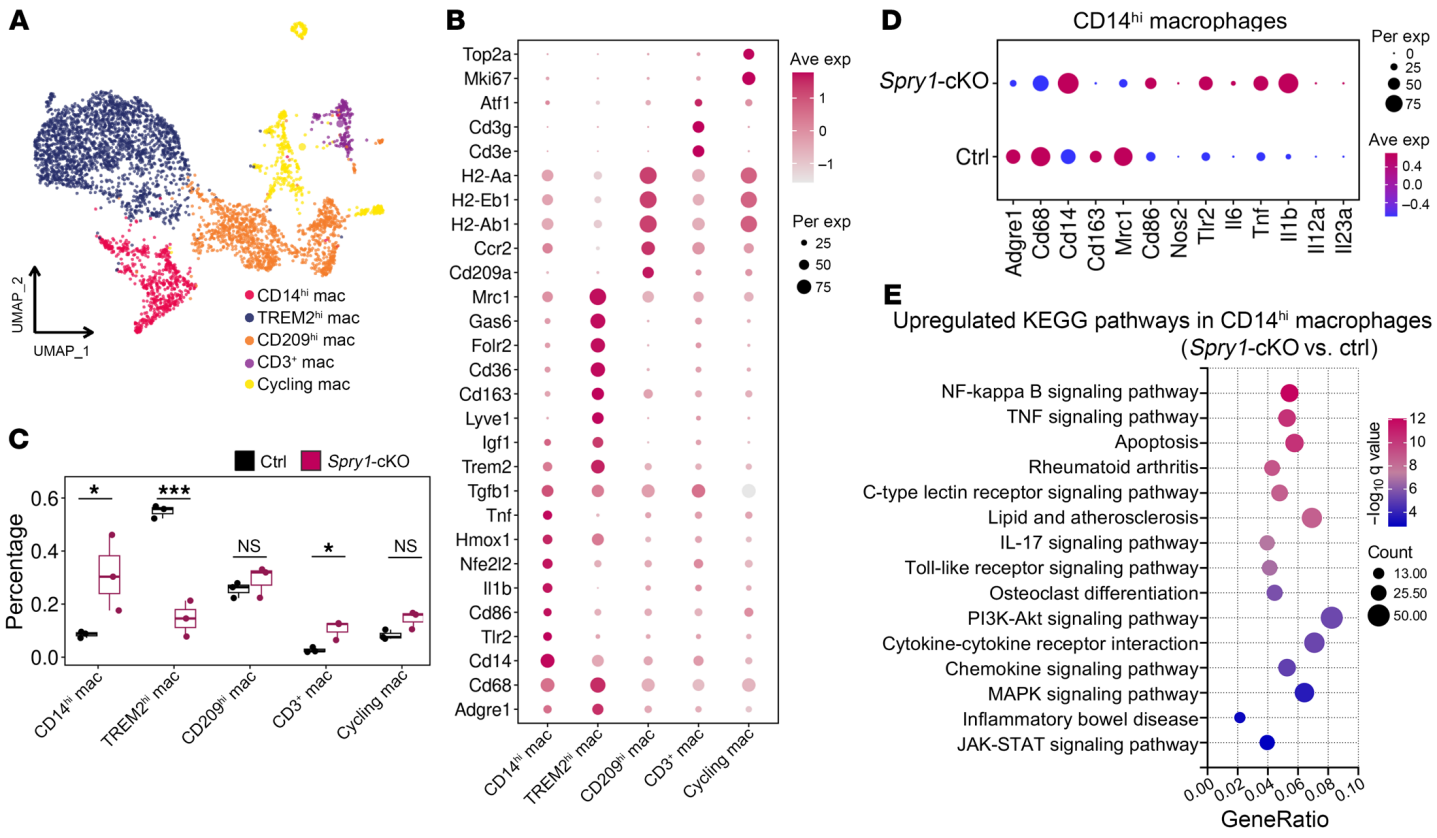
Periarticular CD14^hi^ macrophages play a predominant proinflammatory role in the development of psoriatic arthritis–like inflammation. (**A**) UMAP plots of macrophages from the periarticular tissue of control and *Spry1*-cKO mice by scRNA-Seq, showing 5 clusters (*n* = 3). (**B**) Dot plots showing scaled expression of selected marker genes for macrophage subsets defined in **A**. (**C**) Comparison of the proportions of periarticular macrophage subsets between control and *Spry1*-cKO mice. (**D**) Dot plots showing the scaled expression of genes linked to activation and polarization in periarticular CD14^hi^ macrophages from control and *Spry1*-cKO mice. (**E**) KEGG pathway enrichment of upregulated DEGs (*Spry1*-cKO vs. control) in periarticular CD14^hi^ macrophages (adjusted *P* < 0.01). *P* values were determined using 2-tailed unpaired Student’s *t* test (**C**). *P < 0.05, ***P < 0.001.

Based on the above findings, macrophages were the main source of *Tnf* in the periarticular tissue of *Spry1*-cKO mice (Figure 4C and Supplemental Figure 7A). We further confirmed the crucial pathogenic role of macrophages in the development of PsA-like inflammation by injecting clodronate liposomes (for macrophage depletion) into the peritoneal cavity and paws of *Spry1*-cKO mice, which ameliorated PsA-like inflammation (Supplemental Figure 8). Five macrophage subsets were identified after subclustering, including CD14^hi^ macrophages (*Cd14, Cd86, Tlr2, Il1b,* and *Tnf*), TREM2^hi^ macrophages (*Trem2, Lyve1,* *Cd163, Folr2*, and *Mrc1*), CD209^hi^ macrophages (*Cd209a, Ccr2, H2-Ab1,* and *H2-Aa*), CD3^+^ macrophages (*Cd3e* and *Cd3g*), and cycling macrophages (*Mki67* and *Top2a*) (Figure 5, A and B). Since the CD14^hi^ subset was the largest and most significantly increased periarticular macrophage population in *Spry1*-cKO mice compared with control mice (Figure 5C) and presented with upregulated M1-like proinflammatory markers (*Cd86, Nos2, Il6, Il1b,* and *Tnf*) (Figure 5D), we performed Kyoto Encyclopedia of Genes and Genomes (KEGG) pathway enrichment analysis of upregulated DEGs (*Spry1*-cKO vs. control) in CD14^hi^ macrophages. Importantly, the NF-κB signaling pathway, TNF signaling pathway, rheumatoid arthritis, IL-17 signaling pathway, osteoclast differentiation, cytokine-cytokine receptor interaction, and chemokine signaling pathway were highly activated in periarticular CD14^hi^ macrophages of *Spry1*-cKO mice (Figure 5E).

**Figure 6:**
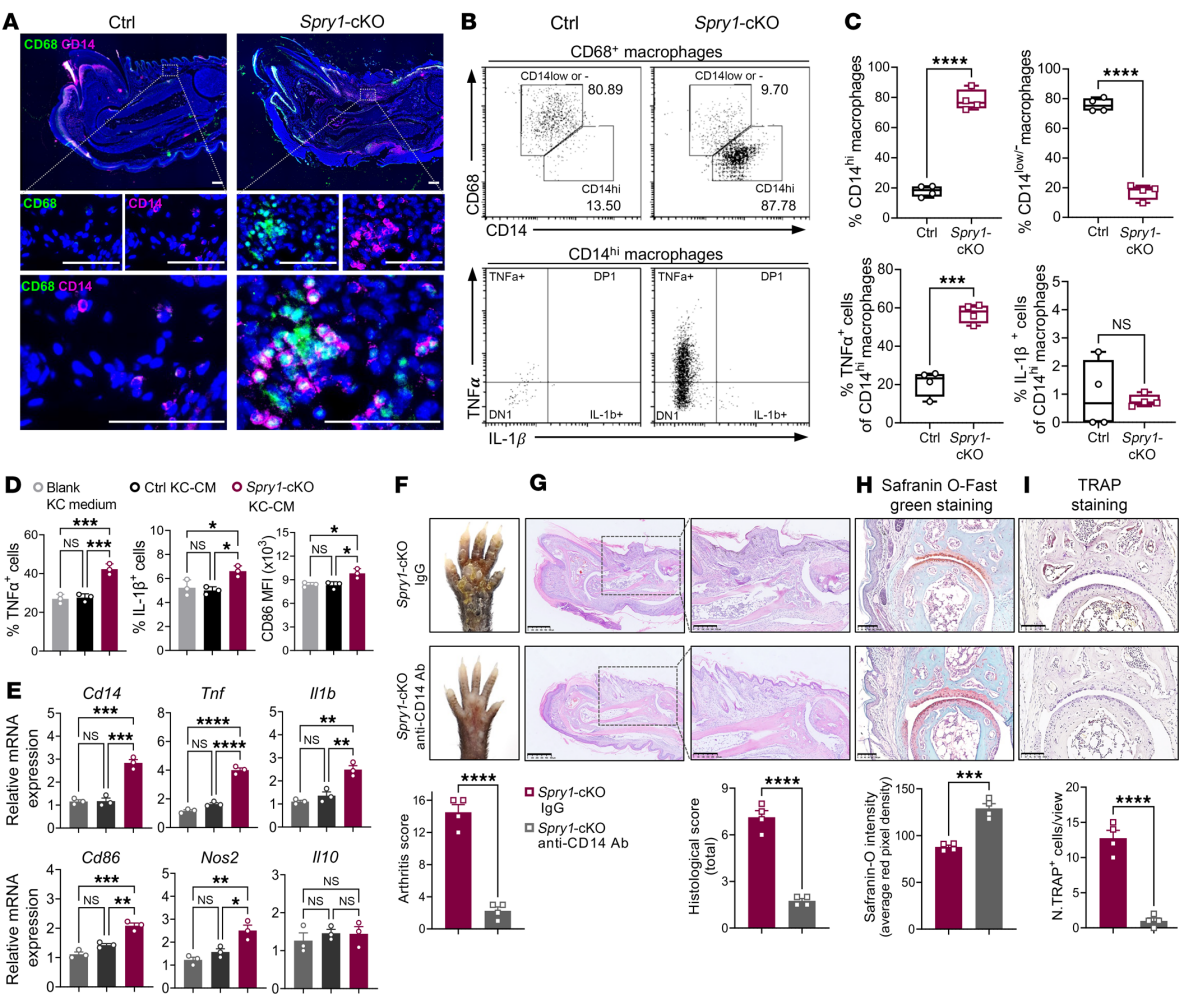
Periarticular CD14^hi^ macrophages aggravate psoriatic arthritis–like inflammation by producing TNF-α. (**A**) Immunofluorescence staining of CD68 and CD14 in the digits from paws of control and *Spry1*-cKO mice. Boxed areas are magnified below. Scale bar: 50 μm. (**B** and **C**) Flow cytometric plots (**B**) and frequencies (**C**) of CD68^+^CD14^hi^ macrophages, TNF-α^+^CD68^+^CD14^hi^ macrophages, IL-1β^+^CD68^+^CD14^hi^ macrophages, and CD68^+^CD14^lo/–^ macrophages in the periarticular tissue from control and *Spry1*-cKO mice (*n* = 4). (**D**) Flow cytometric analysis of TNF-α, IL-1β, and CD86 expression in RAW264.7 cells treated with blank keratinocyte medium, control KC-CM, and *Spry1*-cKO KC-CM for 24 hours, followed by incubation with 50 ng/mL LPS and brefeldin A (a protein transport inhibitor) for 6 hours (*n* = 3). (**E**) Relative mRNA expression of genes associated with macrophage activation and polarization in RAW264.7 cells treated with blank KC medium, control KC-CM, and *Spry1*-cKO KC-CM for 24 hours (*n* = 3). (**F**–**I**) Representative macroscopic views (**F**), H&E staining (left scale bar: 500 μm; right scale bar: 250 μm) (**G**), Safranin O-Fast green staining (scale bar: 100 μm) (**H**), and TRAP staining (scale bar: 100 μm) (**I**) of the paws from age- and sex-matched *Spry1*-cKO mice treated with anti-CD14 antibody and isotype antibody (IgG). Lower panels show quantification of arthritis scores, total histological scores, Safranin-O intensity, and TRAP^+^ osteoclasts, respectively (*n* = 4). Data are shown as mean ± SEM. *P* values were determined using 2-tailed unpaired Student’s *t* test (**C** and **F**–**I**) and 1-way ANOVA (**D** and **E**). *P < 0.05, **P < 0.01, ***P < 0.001, ****P < 0.0001.

Immunofluorescence staining and flow cytometric analysis of CD68^+^CD14^+/hi^ macrophages in the periarticular tissue of *Spry1*-cKO and control mice showed consistent results with the scRNA-Seq data, except for IL-1β expression (Figure 6, A–C, and Supplemental Figure 9A). Given that RAW264.7 cells (mouse macrophage cell line) express CD68 and CD14 (Supplemental Figure 9B), we then incubated RAW264.7 cells with blank keratinocyte medium, control keratinocyte-conditioned medium (KC-CM), or *Spry1*-cKO KC-CM for 24 hours to determine whether the mediators secreted by keratinocytes would affect CD14^+/hi^ macrophages in vitro. As expected, protein levels of TNF-α, IL-1β, and CD86, as well as mRNA levels of *Cd14, Tnf, Il1b, Cd86*, and *Nos2*, were upregulated in RAW264.7 cells after *Spry1*-cKO KC-CM incubation, with the most pronounced upregulation of TNF-α (Figure 6, D and E, and Supplemental Figure 9, C and D). To further confirm the critical role of periarticular CD14^hi^ macrophages, we injected anti-CD14 antibody (atibuclimab) into the peritoneal cavity and paws of *Spry1*-cKO mice during tamoxifen induction. Of note, blocking CD14 significantly alleviated PsA-like skin and joint inflammation, including reduced epidermal hyperplasia, inflammatory infiltration, enthesitis, cartilage damage, and bone erosions (Figure 6, F–I, and Supplemental Figure 9, E–G). Overall, these results demonstrated that periarticular CD14^hi^ macrophages play a predominant pathogenic role in the development of PsA-like inflammation by producing the key proinflammatory cytokine TNF-α.

### Keratinocyte-derived CXCL10 binds to CD14 and mediates a proinflammatory response in periarticular CD14^hi^ macrophages.

**Figure 7:**
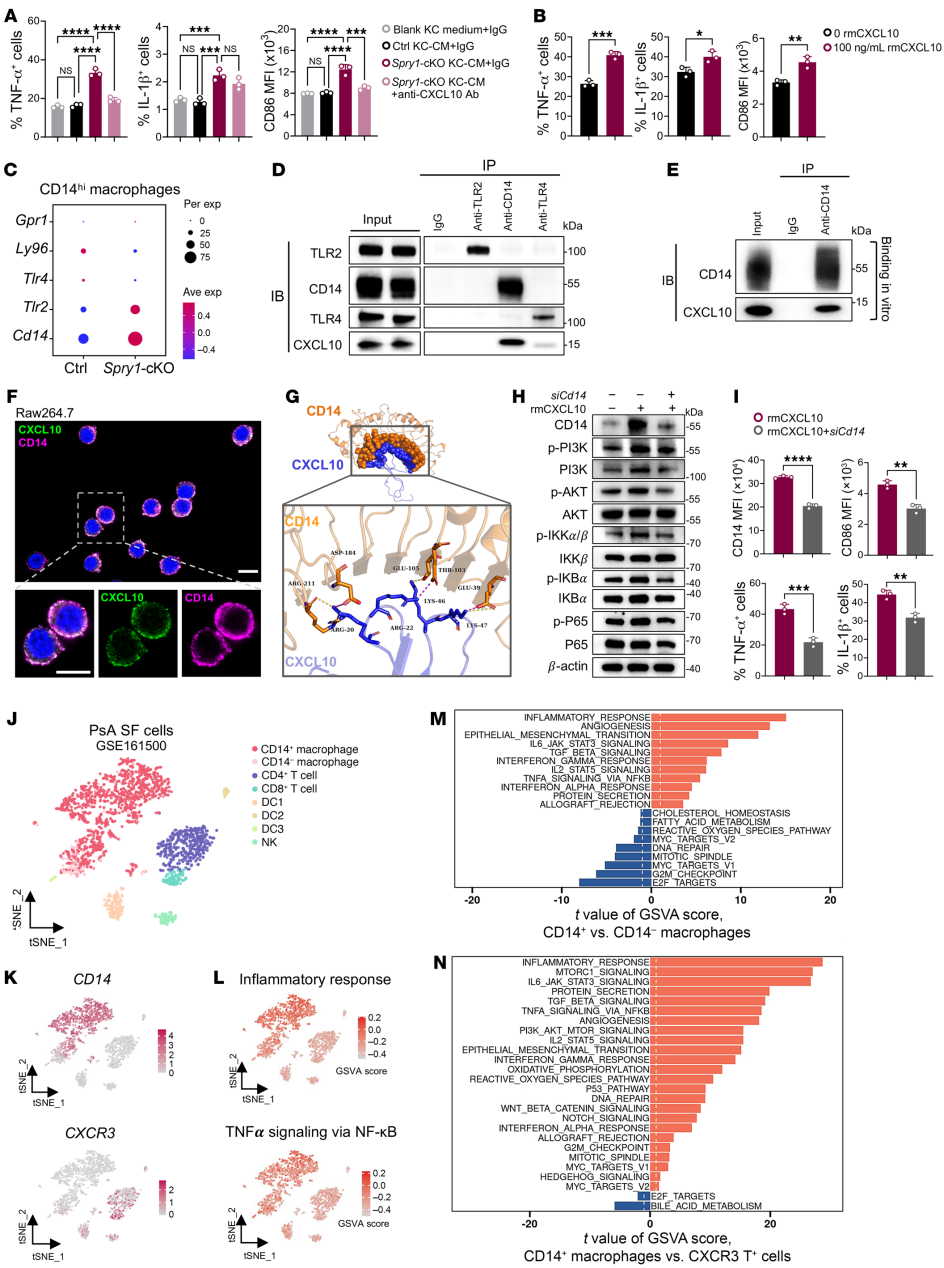
Keratinocyte-derived CXCL10 binds to CD14 and mediates the proinflammatory response in periarticular CD14^hi^ macrophages. (**A** and **B**) Flow cytometric analysis of TNF-α, IL-1β, and CD86 expression in RAW264.7 cells treated with blank keratinocyte medium plus IgG, control KC-CM plus IgG, *Spry1*-cKO KC-CM plus IgG, and *Spry1*-cKO KC-CM plus 2 μg/mL anti-CXCL10 antibody for 24 hours (**A**), or treated with or without 100 ng/mL recombinant murine CXCL10 for 24 hours (**B**), followed by incubation with 50 ng/mL LPS and BFA for 6 hours (*n* = 3). (**C**) Dot plots showing scaled expression (scRNA-Seq) of potential CXCL10 receptor genes mouse periarticular CD14^hi^ macrophages. (**D**) Co-immunoprecipitation of TLR2, TLR4, CD14, and CXCL10 in RAW264.7 cells after recombinant murine CXCL10 treatment. (**E**) Immunoprecipitation of recombinant murine CD14 and CXCL10 proteins. (**F**) Confocal images of CD14 and CXCL10 colocalization in RAW264.7 cells after treatment with recombinant murine CXCL10. Boxed areas are magnified below. Scale bar: 100 μm. (**G**) Optimized binding mode with the lowest binding energy generated by HDOCK, and key residues for interaction between mouse CXCL10 and CD14. Boxed areas are magnified below. Hydrogen bonds are shown as yellow dashed lines and salt bridges as red dashed lines. (**H**) Immunoblotting analysis of protein levels associated with PI3K/AKT and NF-κB signaling pathways in RAW264.7 cells treated with NC or si*Cd14*, followed by recombinant murine CXCL10 stimulation. (**I**) Flow cytometric analysis of CD14, CD86, TNF-α, and IL-1β expression in RAW264.7 cells treated with NC or si*Cd14*, followed by recombinant murine CXCL10 stimulation and subsequent incubation with LPS and BFA (*n* = 3). (**J**) TSNE plots of 8 immune cell clusters in synovial fluid (SF) from PsA patients (GSE161500). (**K**) TSNE plots showing *CD14* and *CXCR3* expression across clusters defined in **J**. (**L**) TSNE plots showing single cell–level enrichment of inflammatory response and TNF-α signaling via NF-κB signatures in all immune clusters defined in **J** by GSVA. (**M** and **N**) GSVA for pathway enrichment of CD14^+^ versus CD14^–^ macrophages (**M**) and CD14^+^ macrophages versus CXCR3^+^ T cells (**N**) in PsA SF (GSE161500). Data are shown as mean ± SEM. *P* values determined using 1-way ANOVA (**A**) and 2-tailed unpaired Student’s *t* test (**B** and **I**). *P < 0.05, **P < 0.01, ***P < 0.001, ****P < 0.0001.

Excessive CXCL10 secreted by SPRY1-deficient keratinocytes has been identified as an upstream factor in the development of PsA-like inflammation in *Spry1*-cKO mice. In addition, *Spry1*-cKO KC-CM promoted the proinflammatory response of RAW264.7 cells (Figure 6, D and E), which was attenuated by anti-CXCL10 antibody (Figure 7A and Supplemental Figure 10, A and B), suggesting that CXCL10 may have a direct effect on CD14^hi^ macrophages. Subsequently, we stimulated RAW264.7 cells with recombinant murine CXCL10 in vitro. The key proinflammatory molecules, including TNF-α, IL-1β, and CD86, were all markedly increased after stimulation (Figure 7B and Supplemental Figure 10C). Given the inability of CXCR3 and TLR4 blockade to attenuate PsA-like inflammation (Figure 4, F and H), CXCL10 may mediate the pathogenic effect by binding to other unknown receptors.

CD14 is a well-known coreceptor that synergistically promotes inflammatory responses to LPS along with TLR4-MD2 complex (40, 41). The expression of the *Cd14* gene in periarticular CD14^hi^ macrophages of *Spry1*-cKO mice was highly upregulated (Figure 7C and Supplemental Figure 10D). Therefore, we reasoned that keratinocyte-derived CXCL10 may bind to CD14 and mediate a proinflammatory response in periarticular CD14^hi^ macrophages. Immunoprecipitation of CXCL10 with antibodies against candidate receptors (CD14, TLR2, and TLR4) was performed in RAW264.7 cells after treatment with 100 ng/mL recombinant murine CXCL10 for 1 hour (mimicking exogenous CXCL10) (Figure 7D). The results revealed a strong interaction between CD14 and CXCL10. Direct binding was further confirmed using a binding assay of purified recombinant murine CD14 and CXCL10 (Figure 7E). Confocal images showed the colocalization of CD14 and CXCL10 in RAW264.7 cells (Figure 7F and Supplemental Figure 10E). To help determine the sites in CD14 and CXCL10 involved in this binding event, we modeled the 3D structure of the CD14-CXCL10 complex using the HDOCK protein-docking server (Figure 7G and Supplemental Figure 10, F and G). The optimized binding mode with the lowest binding energy (–230.46 kcal/mol) and the highest confidence (0.8333) identified several putative bindings, such as Arg211 in CD14 and Arg20 in CXCL10 bound by a hydrogen bond, and Asp184 in CD14 and Arg20 in CXCL10 bound by a salt bridge (Figure 7G and Supplemental Figure 10G).

The production of TNF-α in M1 macrophages is induced by the activation of the NF-κB signaling pathway (42). Additionally, CXCL10 can activate the PI3K/AKT signaling pathway (35), which in turn triggers the activation of NF-κB by enhancing the transcriptional activity of the p65 subunit (43). Notably, the NF-κB, PI3K/AKT, and TNF signaling pathways were all highly activated in periarticular CD14^hi^ macrophages of *Spry1*-cKO mice (Figure 5E). Therefore, we investigated the activation of PI3K/AKT and NF-κB pathways in RAW264.7 cells treated with negative control or si*Cd14*, followed by stimulation with 100 ng/mL recombinant murine CXCL10 for 24 hours. The results showed that enhanced activation of PI3K/AKT and NF-κB signaling pathways by CXCL10 was attenuated after CD14 knockdown (Figure 7H). The downregulated expression of CD14, CD86, TNF-α, and IL-1β was further confirmed by flow cytometry (Figure 7I and Supplemental Figure 10H). These results suggest that keratinocyte-derived CXCL10 enhances the activation of PI3K/AKT and NF-κB signaling pathways in periarticular CD14^hi^ macrophages of *Spry1*-cKO mice by binding to CD14, thereby inducing massive TNF-α production and driving PsA-like inflammation. The CXCL10-CD14 binding bridges the crosstalk between the skin and joints.

To support these findings with human data, we analyzed the scRNA-Seq dataset (GEO GSE161500) of synovial fluid cells from patients with PsA (44). Importantly, the synovial fluid of patients with PsA was predominantly composed of CD14^+^ macrophages (Figure 7J). *CD14* was predominantly expressed by macrophages, and *CXCR3* was predominantly expressed by T cells, but the abundance of *CD14* was substantially higher than that of *CXCR3* (Figure 7K). Gene set variation analysis (GSVA) indicated that the inflammatory response and TNF-α signaling through the NF-κB pathway were more enriched in *CD14*-expressing cells, especially CD14^+^ macrophages (Figure 7L). A comparison between CD14^+^ and CD14^–^ macrophages revealed that CD14^+^ macrophages had a higher abundance of inflammation-related pathways, such as inflammatory response, angiogenesis, IL-6/JAK/STAT signaling, IFN-γ response, TNF-α signaling through NF-κB, and protein secretion pathways. On the other hand, CD14^–^ macrophages were more enriched in metabolism- and cell cycle–related pathways (Figure 7M). Furthermore, the pathways related to inflammation were also more prevalent in CD14^+^ macrophages than in CXCR3^+^ T cells (Figure 7N). These findings support the notion that CD14^+^ macrophages have a crucial proinflammatory impact on the periarticular environment (synovial fluid) in patients with PsA, and CD14-mediated signaling pathways are more influential than CXCR3-mediated signaling pathways, as both are downstream of CXCL10 signaling pathways.

## Discussion

The prevention or delay of PsA development in patients with psoriasis is currently a topic of interest in rheumatology (3, 45). However, the pathophysiological relationship between the skin and joints in the progression from psoriasis to PsA remains unclear. Here, we generated epidermis-specific SPRY1-deficient (*Spry1*-cKO) mice and, surprisingly, observed that these mice spontaneously developed psoriasis-like skin lesions and arthritis. Notably, the skin inflammation occurred earlier than the joint inflammation in the paws. These findings indicate that *Spry1*-cKO mice are suitable mouse models for studying PsA, particularly for dissecting the mechanisms involved in the progression from psoriasis to PsA. Using *Spry1*-cKO mice, we discovered that the absence of SPRY1 in keratinocytes leads to an overproduction of CXCL10. This, in turn, promotes the proinflammatory response of periarticular CD14^hi^ macrophages by binding to CD14 instead of CXCR3, which bridges the skin-joint crosstalk and ultimately triggers PsA.

Mouse models of PsA are limited because of the challenge of establishing experimental arthritis models that recapitulate both psoriasiform dermatitis and arthritis. Therefore, most acknowledged mouse models are genetically engineered with spontaneously developed phenotypes, including mice with *JunB/c-Jun* deletion in keratinocytes (46), *K5.Stat3C:F759* transgenic mice (47), and *Klk6*^+^ transgenic mice (48). However, these studies did not investigate the specific link between skin inflammation and arthritis. In *Spry1*-cKO mice, we observed that the inflammation was less frequent in the back skin (70%) compared with the paws (skin and joints, 100%). Furthermore, analysis of a GEO dataset (GSE186063) revealed that SPRY1 gene expression was significantly downregulated in the PsA lesional skin compared with psoriasis lesional skin and ankylosing spondylitis normal skin, which suggests that SPRY1 downregulation in the skin is more strongly associated with PsA than with psoriasis. The higher incidence of typical PsA-like phenotypes in *Spry1*-cKO mice may be explained by the aforementioned findings, further validating them as PsA mouse models. In addition, abnormalities of the spine, sacroiliac joints, pubic symphysis, and nails (cervicothoracic kyphosis, reduced bone density, and inflammatory infiltration) were observed, but the paw joints were most severely affected (dactylitis), probably because the paw joints are close to the skin compared with other joints; another reason may be the continuous physical stress with movement in paw joints, a condition similar to “deep Koebner” (47). On the other hand, dactylitis is a characteristic musculoskeletal lesion of PsA, usually occurring in the early stages (49). Thus, *Spry1*-cKO mice are suitable for studying the onset of PsA.

Keratinocytes have an important role in interorgan communications by secreting soluble mediators, such as IL-1β, IL-6, TNF-α, CXCL1, CXCL10, and CCL20 (9, 50). CXCL10 is a widely reported biomarker for PsA and could predict future PsA development in patients with psoriasis (26–29). This study highlights the critical pathogenic role of excessive keratinocyte-derived CXCL10 in PsA. *CXCL10* gene expression was found to be significantly upregulated in the epidermis/keratinocytes of *Spry1*-cKO mice as well as in the PsA lesional skin compared with psoriasis lesional skin and ankylosing spondylitis normal skin (GEO GSE186063), indicating a stronger correlation between CXCL10 upregulation and PsA. Concomitantly, CXCL10 blockade effectively attenuated the PsA-like phenotypes of *Spry1*-cKO mice. To more convincingly demonstrate the function of keratinocyte-derived CXCL10, it would be preferable to specifically block CXCL10 in keratinocytes in vivo; for example, by crossing with *Cxcl10^fl/fl^* mice.

CXCL10 expression is primarily mediated by the activation of the JAK1/2/STAT1 signaling pathway induced by IFN-γ (32). Our results revealed a connection between SPRY1 and CXCL10 via the JAK1/2/STAT1 signaling pathway in keratinocytes. The deficiency of SPRY1 intensifies the activation of IFN-γ–induced JAK1/2/STAT1 signaling pathway, leading to excessive secretion of CXCL10. Additionally, these results support the effectiveness and safety of tofacitinib and upadacitinib in treating PsA (51, 52), as these JAK inhibitors have been shown to decrease the elevated CXCL10 secretion of SPRY1-deficient keratinocytes. CXCL10 carries out its biological functions mostly through 2 known receptors: CXCR3 and TLR4 (35–37, 53–55). However, our study does not support the pathogenic role of CXCR3 and TLR4 in CXCL10-induced PsA-like inflammation. This led us to search for the mediators of the proinflammatory effects downstream of CXCL10.

TNF-α is a key downstream effector cytokine across diverse areas of PsA (39, 56), and TNF-α inhibitors have been widely used for the treatment of PsA (3, 38, 57), and they have shown efficacy in the prevention of PsA in patients with psoriasis (58). Concomitantly, TNF-α inhibition resulted in marked remission of PsA-like inflammation in *Spry1*-cKO mice. These findings suggest that periarticular CD14^hi^ macrophages, the main source of TNF-α, have a crucial proinflammatory effect in the development of PsA-like inflammation.

CD14 is primarily present as a surface marker on monocytes/macrophages, and it is a multifaceted receptor that plays an important role in recognizing and responding to various pathogen-associated molecular patterns, which are necessary for regulating innate immunity (59). CD14 acts as a coreceptor for presenting LPS to TLR4, leading to downstream signal transduction and the release of proinflammatory cytokine (40, 41). Considering that periarticular CD14^hi^ macrophages in *Spry1*-cKO mice express higher levels of CD14 and exert proinflammatory effects, and that CXCL10 plays a pathogenic role independent of CXCR3 and TLR4, it is plausible that CXCL10 binds to CD14 to mediate downstream effects. This hypothesis is supported by the data obtained from immunoprecipitation in CD14-expressing RAW264.7 macrophages, in vitro binding assays, confocal visualization of the colocalization, and docking model prediction by HDOCK. Furthermore, CXCL10 binding to CD14 enhances the activation of PI3K/AKT/NF-κB signaling pathways, leading to TNF-α production, and inhibiting CD14 decreases TNF-α release and ameliorates PsA-like inflammation. Importantly, we confirmed that CD14^+^ macrophages play a predominant proinflammatory role in human PsA by analyzing an scRNA-Seq dataset (GEO GSE161500) of synovial fluid cells from patients with PsA. The CD14-mediated signaling pathway was more significant than the CXCR3-mediated signaling pathway, as both are downstream signaling pathways of CXCL10. Recent studies have demonstrated that CD14^+^ myeloid populations are enriched in PsA joints and produce proinflammatory proteins, such as TNF-α, IL-1β, osteopontin, and CCL2 (60, 61).

Osteoclasts are specialized cells derived from the monocyte/macrophage hematopoietic lineage that resorb bone, and TRAP typifies the osteoclast lineage (24). Enhanced differentiation and activation of osteoclasts lead to bone erosions in inflammatory arthritis (24, 62). Typically, TRAP^+^ multinucleated osteoclasts are visible on the endosteal, which is the inner surface of the bone. Interestingly, an increased number of TRAP^+^ multinucleated cells was observed on the periosteum (outer surface of the bone) near the paw joints of *Spry1*-cKO mice (Supplemental Figure 11A); moreover, these periosteal TRAP^+^ cells were significantly reduced by macrophage depletion, CD14 blockade, and CXCL10 blockade (Supplemental Figure 11, B–D). Therefore, we hypothesized that periosteal TRAP^+^ cells may differentiate from periarticular CD14^hi^ macrophages in *Spry1*-cKO mice and contribute to articular bone damage as osteoclasts. By analyzing the expression of RANK and CD115 (osteoclast precursor markers) (63, 64) with flow cytometry and transcriptional pattern of osteoclast differentiation in scRNA-Seq data, we found that periarticular CD14^hi^ macrophages of *Spry1*-cKO mice are more prone to differentiate into osteoclasts (Supplemental Figure 11, E–I). However, a reporter system such as crossing with fluorescent protein knock-in mice will be helpful to validate the osteoclast differentiation from periarticular CD14^hi^ macrophages (65).

To investigate whether the increased periarticular CD14^hi^ macrophages in *Spry1*-cKO mice originated from circulating or tissue-resident cells, we performed parabiosis experiments. Parabiosis surgery was attempted at various time points during TAM induction, but the *Spry1*-cKO mice in the pairs were too weak to survive beyond 2 weeks, which compromised the validity of the parabiosis experiments (66). Alternatively, we adoptively transferred bone marrow–derived macrophages from *Spry1*-cKO mice (CD45.2) after recombinant murine CXCL10 stimulation to congenic WT CD45.1 mice by tail vein injection (Supplemental Figure 12). Four weeks after the first transfer, no signs of skin or joint inflammation were observed in the recipient mice. Furthermore, no donor bone marrow–derived macrophages were detected in the periarticular tissue of recipient mice, indicating periarticular CD14^hi^ macrophages are maintained independent of circulating cells and are likely tissue-resident. However, these experiments do not reveal the specific migration history of these cells, specifically, whether they directly migrated from the skin (67). Genetic labeling, a tamoxifen-inducible system, and time-lapse imaging may be helpful for further exploration.

We acknowledge that some limitations to this study deserve future exploration. First, further elucidation of the mechanisms underlying the preference of CXCL10 binding to CD14 rather than CXCR3 in the periarticular tissue of PsA is required. Second, as mentioned above, it will be helpful to specifically block CXCL10 in keratinocytes in vivo and to establish a reporter system to track the migration or differentiation of periarticular CD14^hi^ macrophages. Third, the above findings need to be confirmed in human PsA samples, despite the challenges in acquiring them.

In conclusion, this study reveals that the alterations in the epidermis are sufficient to drive joint inflammation. Excessive CXCL10 secreted by SPRY1-deficient keratinocytes fuels the proinflammatory response of periarticular CD14^hi^ macrophages by binding to CD14, which bridges the skin-joint crosstalk and ultimately triggers PsA. This keratinocyte SPRY1/CXCL10/periarticular CD14^hi^ macrophage/TNF-α axis provides insights into potential therapeutic targets for early intervention to prevent this progression. Clinical trials have been carried out on antibodies targeting CXCL10 or CD14 in other inflammatory diseases, including rheumatoid arthritis (68), amyotrophic lateral sclerosis (69), and COVID-19 (70). Based on our findings, it is feasible to extend these trials to PsA.

## Methods

### Sex as a biological variable.

Our study examined male and female humans and animals, and similar findings were reported for both sexes.

### Study participants.

Skin samples were obtained from patients with psoriasis or atopic dermatitis, as well as from healthy donors undergoing plastic surgery. Serum samples were collected from healthy donors and patients with psoriasis. Signed consent forms were obtained from all participants. Participant information is detailed in Supplemental Table 1.

### Mice.

All mice used in this study were on C57BL/6 genetic background. *Spry1^fl/fl^* mice were purchased from Cyagen Biosciences Inc. Keratin 14-Cre transgenic mice (STOCK Tg(KRT14-Cre/ERT)20Efu/J, *K14^CreERT^*, strain 005107) (71) were purchased from The Jackson Laboratory. C57BL/6 mice were purchased from Shanghai SLAC Laboratory Animal Co., Ltd. *Spry1^fl/fl^* mice were crossed with *K14^CreERT^* mice to generate *K14^CreERT^Spry1^fl/fl^* mice. All mice were bred and maintained under specific pathogen–free conditions in accordance with the *Guide for the Care and Use of Laboratory Animals* (National Academies Press, 2011). Both males and females were used in this study, and no significant sex-dependent differences were found in the reported experiments. Mice were randomly assigned to experimental groups, except in experiments that required specific genotypes. Sex- and age-matched mice between 3 and10 weeks of age were used.

### Flow cytometry and FACS.

Cell suspensions were prepared as described in the Supplemental Methods. For surface marker staining, cells were stained with Zombie UV Fixable Viability Kit (BioLegend) for 15 minutes, blocked with Fc receptor blocker TruStain FcX (BioLegend) for 10 minutes, and were then incubated with mixed surface marker antibodies for 30 minutes at 4°C in the dark. For intracellular cytokine staining, cells were first stimulated with Cell Activation Cocktail (containing brefeldin A, BioLegend) or 2 μg/mL resiquimod (Sigma-Aldrich) in the presence of brefeldin A (GolgiPlug, BD Biosciences) for 6 hours. After stimulation, cells were stained with surface marker antibodies for 30 minutes at 4°C in the dark, followed by fixation and permeabilization with BD Cytofix/Cytoperm (BD Biosciences) or Foxp3/Transcription Factor Staining Buffer Set (eBioscience), and were then incubated with cytokine antibodies for 30 minutes at 4°C in the dark. All antibodies used for flow cytometry and FACS are listed in Supplemental Table 2. Flow cytometry was performed using a CytoFlex LX (Beckman Coulter) flow cytometer, and FACS was performed using a BD FACSAria III cell sorter (BD Biosciences). Data were analyzed with FlowJo v10 and CytExpert software.

### MRI and micro-CT.

For MRI analysis, mice were under isoflurane anesthesia (1.5%–2.0% in oxygen), with body temperature (37°C) and respiratory rate (40–55/min) monitored and maintained throughout the experiment. Mice received MRI radiography of hind limbs using a 4.7 T spectrometer (Bruker) with an active shielding gradient (20 G/cm, 80 μs rise time). Multi-slice T2-weighted images were obtained with 1 mm slice thickness and 128 × 128 data matrix.

For micro-CT analysis, mice were euthanized and fixed in 4% PFA for 48 hours, then scanned using the SkyScan 1276 instrument (Bruker-MicroCT) with the following parameters: 80 kV, 450 μA, 45 μm. Micro-CT images were reconstructed using CTVOX v3.3 and analyzed using CTAN v1.07 (both Bruker). Cervicothoracic kyphosis was evaluated by taking point-to-point measurements in the anterior-posterior and dorsal-ventral aspects (48). Bone volume fraction (bone volume/total volume [BV/TV]) was used to assess the bone density in the paw joints, sacroiliac joints, and pubic symphysis.

### Bulk RNA-Seq.

The epidermis and dermis were separated after digestion of mouse back skin tissue with 5% dispase overnight at 4°C. Bulk RNA-Seq was then performed on total RNA from the epidermis and dermis separately. Libraries were prepared using a TruSeq PE Cluster Kit v3-cBot-HS (Illumina), quantified on an Agilent Bioanalyzer 2100 system, and sequenced on the Illumina NovaSeq platform, and 150 bp paired-end reads were generated according to the manufacturer’s instructions. After quality control, the clean reads were aligned to the reference genome using Hisat2 v2.0.5, and mapped read counts were estimated by FeatureCounts v1.5.0-p3. Differential expression analysis was performed by the DESeq2 R package (1.20.0). *P* values were adjusted using the Benjamini-Hochberg method. Adjusted *P* less than 0.05 and |log_2_ fold change| greater than 0.5 were set as the threshold to define statistical significance. Gene Ontology and KEGG pathway analysis of DEGs was implemented by the clusterProfiler R package. An adjusted *P* less than 0.05 was considered significant. Integrated enrichment analysis of the epidermal and dermal DEGs was performed using the Metascape web tool (https://metascape.org/gp/index.html#/main/step1). For cross-species analysis, case-insensitive gene symbol matching was performed using the RNA-Seq dataset of 64 normal skin samples and 58 psoriatic skin samples (GEO GSE13355). GSEA was performed using the “fgsea” function from the “fgsea” R package (version 1.26.0). Novogene Bioinformatics Technology Co. performed the RNA-Seq and assisted in data analysis.

### ScRNA-Seq and data analysis.

Cell suspensions of periarticular tissue from *Spry1*-cKO and control mice 6 weeks after the first tamoxifen induction were prepared as described in the Supplemental Methods. To obtain sufficient immune cells for analysis, 4 mice were pooled as 1 sample for the control group (12 control mice in total for *n* = 3), and then CD45^+^ and CD45^–^ cells were sorted and mixed at a ratio of 3:1 in both groups. The cells were loaded on a 10X Chromium Controller (10X Genomics) according to the manufacturer’s protocol to generate gel-bead in emulsions, which were used to generate barcoded, full-length cDNA through reverse transcription reactions. Barcoded scRNA-Seq libraries were prepared using the Chromium Single Cell 3′ Reagent Kit. After final preparation and QC were performed, the libraries were sequenced on a HiSeq X Ten platform (Illumina), and 150 bp paired-end reads were generated.

### Immunoprecipitation.

For identification of CXCL10 protein partners, RAW264.7 cells were serum-starved for 5 hours and then treated with 100 ng/mL recombinant murine CXCL10 for 1 hour. After washing 3 times with PBS, cells were lysed in RIPA buffer (Beyotime) supplemented with protease and phosphatase inhibitor cocktail (Thermo Fisher Scientific). The lysates were incubated with anti-TLR2 antibody (Abcam, ab209217, 1:30), anti-CD14 antibody (Abcam, ab221678, 1:30), anti-TLR4 antibody (Proteintech, 66350-1-Ig, 1:100), or IgG overnight at 4°C under rotation and then incubated with protein-A/G magnetic beads (MedChemExpress) overnight at 4°C under rotation. The immunoprecipitates were washed 5 times with wash buffer, eluted with 1× SDS loading buffer, and then subjected to immunoblotting.

### Protein-protein binding assays.

A 5 μM volume of recombinant murine CD14 (BioLegend) was incubated with 5 μM recombinant murine CXCL10 (PeproTech) in a total volume of 400 μL equilibrium buffer (containing 20 mM Tris-HCl, pH 7.5, 250 mM NaCl) (72) for 30 minutes at room temperature. After incubation, proteins were immunoprecipitated by incubation (at 4°C under rotation) with anti-CD14 antibodies (Abcam, ab221678,1:30) overnight and then with protein A/G–magnetic beads (MedChemExpress) overnight. The immunoprecipitates were washed 5 times with wash buffer and eluted with 1× SDS loading buffer. Precipitated CD14 and CXCL10 were detected by immunoblotting.

### CXCL10 and CD14 colocalization.

RAW264.7 cells were seeded in the confocal dish (ibidi GmbH) and serum-starved for 5 hours before treatment with 100 ng/mL recombinant murine CXCL10 for 1 hour. After washing 3 times with PBS, cells were fixed in 4% PFA for 15 minutes at room temperature, permeabilized with 0.1% Triton X-100 for 10 minutes, and blocked with 3% BSA and 5% goat serum for 1 hour at room temperature. Subsequently, cells were incubated with anti-CXCL10 antibody (Bioss, bs-1502R, 1:100) and anti-CD14 antibody (Abcam, ab221678, 1:1,000) overnight at 4°C and then incubated with corresponding Alexa Fluor 488/555–conjugated secondary antibodies (1:1,000, Thermo Fisher Scientific) for 2 hours at room temperature in the dark, followed by counterstaining with DAPI (Roche) for 15 minutes. After washing in PBS, cells were mounted with antifade mounting medium (Solarbio). Images were captured using LAS X software (Leica) on a confocal microscope (Leica STELLARIS 5).

### Molecular docking.

CXCL10-CD14 complex predictions were performed by Phadcalc. Briefly, the crystal structures of CXCL10 (2R3Z) and CD14 (1WWL) were obtained from the RCSB Protein Data Bank. CXCL10 and CD14 docking was performed using the HDOCK server (http://hdock.phys.hust.edu.cn/) (73). Molecular docking and conformation scoring were performed using the empirically based iterative scoring function ITScorePP, and the top 10 conformations with strong binding ability were further analyzed for credibility using the confidence score (greater than 0.7 indicating a reliable docking score and a high likelihood of molecular binding). The conformation with the first docking score and confidence score was selected for subsequent analysis: 3D modeling was conducted in PyMOL software (v2.4); 2D interaction analysis of binding interface residues, interaction types, and distances was determined using Maestro (detailed information is provided in Supplemental Table 4).

### Supplemental material.

Expanded methods, supplemental figures, and supplemental tables are available in the supplemental material.

### Statistics.

Data presented in this study are from 1 representative experiment of at least 3 independent experiments. Data are presented as mean ± SEM. Statistical analysis was performed using GraphPad Prism 9.0 software or R. Unpaired or paired 2-tailed Student’s *t* tests were used to compare differences between 2 independent groups. One-way ANOVA followed by Tukey’s multiple-comparison test was used to compare differences among multiple groups. Details of specific comparisons are stated in the figure legends. *P* values less than 0.05 were considered statistically significant.

### Study approval.

For human studies, written informed consent from the donors was obtained. Human samples were used in full agreement with the approval of the Human Research Ethics Committee of the Second Affiliated Hospital, Zhejiang University School of Medicine (2020-101). Animal procedures were performed according to NIH guidelines and were approved by the Animal Ethics Committee of the Second Affiliated Hospital of Zhejiang University School of Medicine (2019-086).

### Data availability.

All data associated with this study are present in this article or in the supplemental materials, and values for all data points in graphs are reported in the Supporting Data Values file. The raw bulk RNA-Seq and scRNA-Seq data of mice generated in our study have been deposited in the NCBI’s GEO database under accession numbers GSE289141 and GSE289142. Materials will be made available upon request.

## Author contributions

FX conceptualized the study, designed and performed experiments, analyzed and interpreted data, and wrote the original manuscript. YZC and XYY designed and performed experiments, analyzed data, and helped write the manuscript. YXZ, XBC, HZ, ZYW, and YZ performed experiments and analyzed data. YL and YYL provided advice on methodologies and helped perform experiments. LRY, NCF, SQC, and XYC helped perform experiments and acquire data. MZ provided reagents and shared key methodologies. YY provided intellectual expertise and helped conceptualize the study. XYM conceptualized and supervised the study, analyzed and interpreted results, acquired the funding, and edited the manuscript. All the authors reviewed and contributed to editing the manuscript before submission.

## Supplementary Material

Supplemental data

Unedited blot and gel images

Supporting data values

## Version 1 06/05/2025

In-Press Preview

## Version 2 08/01/2025

Electronic publication

## References

[1] Armstrong AW, Read C (2020). Pathophysiology, clinical presentation, and treatment of psoriasis: a review. JAMA.

[2] Michalek IM (2017). A systematic review of worldwide epidemiology of psoriasis. J Eur Acad Dermatol Venereol.

[3] Scher JU (2019). Preventing psoriatic arthritis: focusing on patients with psoriasis at increased risk of transition. Nat Rev Rheumatol.

[4] Ritchlin CT (2017). Psoriatic arthritis. N Engl J Med.

[5] Scotti L (2018). Prevalence and incidence of psoriatic arthritis: a systematic review and meta-analysis. Semin Arthritis Rheum.

[6] FitzGerald O (2021). Psoriatic arthritis. Nat Rev Dis Primers.

[7] Acosta Felquer ML (2022). Treating the skin with biologics in patients with psoriasis decreases the incidence of psoriatic arthritis. Ann Rheum Dis.

[8] Alinaghi F (2019). Prevalence of psoriatic arthritis in patients with psoriasis: A systematic review and meta-analysis of observational and clinical studies. J Am Acad Dermatol.

[9] Dainichi T (2018). The epithelial immune microenvironment (EIME) in atopic dermatitis and psoriasis. Nat Immunol.

[10] Nestle FO (2009). Skin immune sentinels in health and disease. Nat Rev Immunol.

[11] Kabashima K (2019). The immunological anatomy of the skin. Nat Rev Immunol.

[12] Griffiths CEM (2021). Psoriasis. Lancet.

[13] van de Kerkhof PC (2022). From empirical to pathogenesis-based treatments for psoriasis. J Invest Dermatol.

[14] Najm A (2023). Phenotypic heterogeneity in psoriatic arthritis: towards tissue pathology-based therapy. Nat Rev Rheumatol.

[15] Ross FP, Christiano AM (2006). Nothing but skin and bone. J Clin Invest.

[16] Liang W (2022). Skin chronological aging drives age-related bone loss via secretion of cystatin-A. Nat Aging.

[17] Uluckan O (2016). Chronic skin inflammation leads to bone loss by IL-17-mediated inhibition of Wnt signaling in osteoblasts. Sci Transl Med.

[18] Hanafusa H (2002). Sprouty1 and Sprouty2 provide a control mechanism for the Ras/MAPK signalling pathway. Nat Cell Biol.

[19] Wang P (2018). The role of Sprouty1 in the proliferation, differentiation and apoptosis of epidermal keratinocytes. Cell Prolif.

[20] Zhou Y (2022). Sprouty1 exerts a preventive effect on the initiation of psoriasis by inhibiting innate immune antimicrobial peptide cathelicidin and immunocytes. Cell Prolif.

[21] Deng J (2022). Multi-omics integration reveals a core network involved in host defence and hyperkeratinization in psoriasis. Clin Transl Med.

[22] Johnsson H (2023). Cutaneous lesions in psoriatic arthritis are enriched in chemokine transcriptomic pathways. Arthritis Res Ther.

[23] Quaranta M (2014). Intraindividual genome expression analysis reveals a specific molecular signature of psoriasis and eczema. Sci Transl Med.

[24] Boyle WJ (2003). Osteoclast differentiation and activation. Nature.

[25] Nair RP (2009). Genome-wide scan reveals association of psoriasis with IL-23 and NF-kappaB pathways. Nat Genet.

[26] Abji F (2016). Brief Report: CXCL10 is a possible biomarker for the development of psoriatic arthritis among patients with psoriasis. Arthritis Rheumatol.

[27] Abji F (2020). Declining levels of serum chemokine (C-X-C motif) ligand 10 over time are associated with new onset of psoriatic arthritis in patients with psoriasis: a new biomarker?. Br J Dermatol.

[28] Mulder MLM (2021). Clinical, laboratory, and genetic markers for the development or presence of psoriatic arthritis in psoriasis patients: a systematic review. Arthritis Res Ther.

[29] Penkava F (2020). Single-cell sequencing reveals clonal expansions of pro-inflammatory synovial CD8 T cells expressing tissue-homing receptors in psoriatic arthritis. Nat Commun.

[30] Qiu X (2021). ULK1 inhibition as a targeted therapeutic strategy for psoriasis by regulating keratinocytes and their crosstalk with neutrophils. Front Immunol.

[31] Antonelli A (2014). Chemokine (C-X-C motif) ligand (CXCL)10 in autoimmune diseases. Autoimmun Rev.

[32] Liu M (2011). CXCL10/IP-10 in infectious diseases pathogenesis and potential therapeutic implications. Cytokine Growth Factor Rev.

[33] Sathyanarayana P (2012). Spry1 as a novel regulator of erythropoiesis, EPO/EPOR target, and suppressor of JAK2. Blood.

[34] De Filippo K (2013). Mast cell and macrophage chemokines CXCL1/CXCL2 control the early stage of neutrophil recruitment during tissue inflammation. Blood.

[35] Schulthess FT (2009). CXCL10 impairs beta cell function and viability in diabetes through TLR4 signaling. Cell Metab.

[36] Shang C (2022). CXCL10 conditions alveolar macrophages within the premetastatic niche to promote metastasis. Cancer Lett.

[37] Lee JH (2017). Pathogenic roles of CXCL10 signaling through CXCR3 and TLR4 in macrophages and T cells: relevance for arthritis. Arthritis Res Ther.

[38] Schett G (2022). Psoriatic arthritis from a mechanistic perspective. Nat Rev Rheumatol.

[39] Schett G (2021). Reframing immune-mediated inflammatory diseases through signature cytokine hubs. N Engl J Med.

[40] Wright SD (1990). CD14, a receptor for complexes of lipopolysaccharide (LPS) and LPS binding protein. Science.

[41] Ryu JK (2017). Reconstruction of LPS transfer cascade reveals structural determinants within LBP, CD14, and TLR4-MD2 for efficient LPS recognition and transfer. Immunity.

[42] Sica A, Mantovani A (2012). Macrophage plasticity and polarization: in vivo veritas. J Clin Invest.

[43] Liu R (2020). PI3K/AKT pathway as a key link modulates the multidrug resistance of cancers. Cell Death Dis.

[44] Abji F (2020). Proteinase-mediated macrophage signaling in psoriatic arthritis. Front Immunol.

[45] Koehm M, Behrens F (2023). Association between biological immunotherapy for psoriasis and time to incident inflammatory arthritis: limitations and opportunities. RMD Open.

[46] Zenz R (2005). Psoriasis-like skin disease and arthritis caused by inducible epidermal deletion of Jun proteins. Nature.

[47] Yamamoto M (2015). Psoriatic inflammation facilitates the onset of arthritis in a mouse model. J Invest Dermatol.

[48] Billi AC (2020). KLK6 expression in skin induces PAR1-mediated psoriasiform dermatitis and inflammatory joint disease. J Clin Invest.

[49] McGonagle D (2019). Pathophysiology, assessment and treatment of psoriatic dactylitis. Nat Rev Rheumatol.

[50] Lowes MA (2014). Immunology of psoriasis. Annu Rev Immunol.

[51] Mease P (2017). Tofacitinib or adalimumab versus placebo for psoriatic arthritis. N Engl J Med.

[52] McInnes IB (2021). Trial of upadacitinib and adalimumab for psoriatic arthritis. N Engl J Med.

[53] Soejima K, Rollins BJ (2001). A functional IFN-gamma-inducible protein-10/CXCL10-specific receptor expressed by epithelial and endothelial cells that is neither CXCR3 nor glycosaminoglycan. J Immunol.

[54] Luster AD (1995). The IP-10 chemokine binds to a specific cell surface heparan sulfate site shared with platelet factor 4 and inhibits endothelial cell proliferation. J Exp Med.

[55] Margulieux KR (2016). CXCL10 acts as a bifunctional antimicrobial molecule against Bacillus anthracis. mBio.

[56] Bravo A, Kavanaugh A (2019). Bedside to bench: defining the immunopathogenesis of psoriatic arthritis. Nat Rev Rheumatol.

[57] Van den Bosch F, Coates L (2018). Clinical management of psoriatic arthritis. Lancet.

[58] Singla S (2023). Association between biological immunotherapy for psoriasis and time to incident inflammatory arthritis: a retrospective cohort study. Lancet Rheumatol.

[59] Pugin J (1994). CD14 is a pattern recognition receptor. Immunity.

[60] Yager N (2021). Ex vivo mass cytometry analysis reveals a profound myeloid proinflammatory signature in psoriatic arthritis synovial fluid. Ann Rheum Dis.

[61] Floudas A (2022). Distinct stromal and immune cell interactions shape the pathogenesis of rheumatoid and psoriatic arthritis. Ann Rheum Dis.

[62] Jung YK (2019). Osteoclasts in the inflammatory arthritis: implications for pathologic osteolysis. Immune Netw.

[63] Patel C (2022). A new method of bone stromal cell characterization by flow cytometry. Curr Protoc.

[64] Muto A (2011). Lineage-committed osteoclast precursors circulate in blood and settle down into bone. J Bone Miner Res.

[65] Hasegawa T (2019). Identification of a novel arthritis-associated osteoclast precursor macrophage regulated by FoxM1. Nat Immunol.

[66] Wang H (2022). Parabiosis in mice to study tissue residency of immune cells. Curr Protoc.

[67] Masopust D, Soerens AG (2019). Tissue-resident T cells and other resident leukocytes. Annu Rev Immunol.

[68] Yellin M (2012). A phase II, randomized, double-blind, placebo-controlled study evaluating the efficacy and safety of MDX-1100, a fully human anti-CXCL10 monoclonal antibody, in combination with methotrexate in patients with rheumatoid arthritis. Arthritis Rheum.

[69] Gelevski D (2023). Safety and activity of anti-CD14 antibody IC14 (atibuclimab) in ALS: Experience with expanded access protocol. Muscle Nerve.

[70] Mabrey FL (2023). Phase 2, randomized, double-blind, placebo-controlled multi-center trial of the clinical and biological effects of anti-CD14 treatment in hospitalized patients with COVID-19 pneumonia. EBioMedicine.

[71] Vasioukhin V (1999). The magical touch: genome targeting in epidermal stem cells induced by tamoxifen application to mouse skin. Proc Natl Acad Sci U S A.

[72] Louche A (2017). Protein-protein interactions: pull-down assays. Methods Mol Biol.

[73] Yan Y (2017). HDOCK: a web server for protein-protein and protein-DNA/RNA docking based on a hybrid strategy. Nucleic Acids Res.

