# Supplementary material for: CXCL10 secreted by SPRY1-deficient epidermal keratinocytes fuels joint inflammation in psoriatic arthritis via CD14 signaling
Source: J Clin Invest. 2025 Jun 5;135(15):e186135. doi: 10.1172/JCI186135 (PMC12321389; doi:10.1172/JCI186135)
Supplement: Supplemental data [file jci-135-186135-s039.pdf]

Supplemental Methods

Cell culture

To isolate primary mouse keratinocytes, the back skin tissue was cut into small pieces and digested with 5% dispase (Gibco) overnight at 4°C. The epidermal sheet was then separated and further digested with 0.25% trypsin (Thermo Fisher Scientific) for 15 min at 37°C, with gentle shaking, and then neutralized with fetal bovine serum (FBS) (Gibco). The cell suspensions were pipetted, filtered with a 70-μm cell strainer, and centrifuged to pellet the cells, which were then cultured in keratinocyte culture medium (Lonza), 100 U/ml penicillin and 100 mg/mL streptomycin sulfate (Gibco), at 37°C in a humidified atmosphere containing 5% CO_2. The medium was refreshed every 2~3 days. To ensure SPRY1 knockout, the keratinocytes were cultured in the presence of 2.5 nM 4-Hydroxytamoxifen (Sigma-Aldrich) in the keratinocyte culture medium for three consecutive days.

Primary normal human epidermal keratinocytes (NHEKs) and psoriatic human epidermal keratinocytes (PHEKs) were isolated from the skin tissue of healthy donors and psoriasis patients, respectively, using a procedure similar to that described above for mouse skin tissue.

The mouse macrophage cell line RAW264.7 was a kind gift from Dr. Zhao-Ming Ye (Orthopedics Research Institute of Zhejiang University, Hangzhou, Zhejiang, China). RAW264.7 cells were cultured in DMEM (Gibco) supplemented with 10% FBS (Gibco) at 37°C in a humidified atmosphere containing 5% CO_2.

Animal models and in vivo interventions

For induction of Cre recombinase, 100 mg/kg body weight tamoxifen (MedChemExpress) (20 mg/mL solution in corn oil) was administrated to *K14*^{CreERT}*Spry1*$^{f/f}$ mice by oral gavage at three weeks of age for five consecutive weeks (once a day, four days a week). As control, littermates with the respective floxed alleles but without Cre recombinase expression (*Spry1*$^{f/f}$ mice) were treated in the same manner. Clinical measurements and scores were evaluated 6 weeks after the first tamoxifen induction. Ear, back skin, digit, and paw thickness were measured using a caliper gauge. The severity of back skin inflammation was assessed using the Psoriasis Area and Severity Index (PASI) (1): Erythema, scaling, and thickening were independently scored on a scale of 0 to 4: 0, none; 1, mild; 2, moderate; 3, severe; 4, very severe, and the cumulative score (maximum of 12) was determined per mouse. The severity of arthritis was assessed in each of the four paws using a score ranging from 0 to 4 (2): 0, no swelling; 1, mild swelling and erythema; 2, moderate swelling and erythema; 3, severe swelling and erythema; 4, maximal inflammation with joint rigidity, and the cumulative score of four paws (maximum of 16) was determined per mouse.

For in vivo blockade of CXCL10, CXCR3, TNFα, CD14, or CXCL1, anti-mouse CXCL10-neutralizing antibody (R&D Systems), *InVivo*MAb anti-mouse CXCR3 antibody (BioxCell), *InVivo*MAb anti-mouse TNFα antibody (BioxCell), anti-CD14 antibody/Atibuclimab (MedChemExpress), or anti-CXCL1-neutralizing antibody (Invitrogen) was injected into the peritoneal cavity (100 μg) and paws (25 μg for each paw) of *Spry1* cKO mice every other day for three consecutive weeks starting from the

third week of tamoxifen induction. For the control group, sex- and age-matched *Spry1* cKO mice were treated with the corresponding isotype control antibody in the same manner. For in vivo inhibition of TLR4, TAK-242/Resatorvid (MedChemExpress) was injected into the peritoneal cavity (3 mg/kg) and paws (0.75 mg/kg) of *Spry1* cKO mice every other day for three consecutive weeks starting from the third week of tamoxifen induction, and for the control group, sex- and age-matched *Spry1* cKO mice were treated with an equivalent volume of vehicle (PBS containing 1% DMSO). For macrophage depletion, sex- and age-matched *Spry1* cKO mice were injected with clodronate liposomes (LIPOSOMA) or vehicle into the peritoneal cavity (150 μL) and paws (20 μL for each paw) twice a week for three consecutive weeks starting from the third week of tamoxifen induction.

Adoptive transfer of bone marrow-derived macrophages (BMDMs)

BMDMs were generated as previously described (3). Briefly, bone marrow cells were isolated from *Spry1* cKO mice (CD45.2) 6 weeks after the first tamoxifen induction by flushing bone marrow with PBS containing 1% penicillin/streptomycin. The cells were cultured in DMEM supplemented with 10% FBS, 1% penicillin/streptomycin, and 30 ng/mL recombinant murine M-CSF at 37°C 5% CO2. The culture medium was replaced every 2 days for 6 days. On day 7, BMDMs were stimulated with 100 ng/mL recombinant murine CXCL10 for 24h, then collected using a cell scraper and dispersed in PBS. BMDMs (5×10^6 cells/100 μL/mouse) or PBS (100 μL) were transferred into congenic wild-type CD45.1 mice via tail-vein injection once a week for four

consecutive weeks.

Tissue processing

Mice were asphyxiated with CO_2 and the hairs on the ears and back were removed. Ears, back skin (with subcutaneous fat removed), and hind paws were excised. The periarticular tissue of paws was isolated by gently removing all phalanges. The epidermis of back skin was separated after digestion with 5% dispase overnight at 4°C. For histological analysis, the tissue was fixed in 4% paraformaldehyde (PFA). For mRNA and protein analysis, the tissue was ground with a tissue grinder (Jingxin, Shanghai) and lysed with TRIzol reagent (Takara) or RIPA buffer (Beyotime) supplemented with protease and phosphatase inhibitor cocktail (Thermo Fisher Scientific). For cell suspensions, ears (with inner and outer surfaces separated by forceps) and back skin tissue were minced and digested in RPMI 1640 medium (Gibco) supplemented with 1 mg/mL collagenase IV (Sigma), 0.2 mg/mL DNase I (Roche), and 2% FBS for 60 min at 37°C with gentle shaking; periarticular tissue was minced and digested in RPMI 1640 medium (Gibco) supplemented with 1.5 mg/mL collagenase IV (Sigma), 0.5 mg/mL DNase I (Roche), and 2% FBS for 90 min at 37°C with gentle shaking; epidermis was digested in DMEM (Gibco) supplemented with 0.25% trypsin (Gibco) for 15 min at 37°C with gentle shaking. After digestion, the samples were filtered with a 70 μm cell strainer, and the cell pellet was collected after centrifugation at 450 x g for 5 min followed by resuspension in the corresponding medium.

Histopathology and immunohistochemistry

Skin samples were fixed in 4% paraformaldehyde (PFA) and embedded in paraffin; paw samples were decalcified in 10% EDTA (pH 7.2) at 4°C for 4 weeks (renewed every other day) before embedding, and 4.5 μm sections were stained with hematoxylin and eosin (H&E) for histological analysis. The sections were scanned with a NanoZoomer slide scanner (HAMAMATSU Photonics, Japan) and analyzed with NDP.view2 software. Interfollicular epidermal thickness in H&E-stained sections was quantified. To assess the severity of psoriatic arthritis in the paw/digit sections, the following histopathological scoring was used: epidermal thickness, inflammatory infiltrate, fibroplasia was independently scored on a scale of 0 to 3: 0, normal; 1, mild; 2, moderate; 3, severe, and the cumulative score (maximum of 9) was determined.

For immunohistochemistry, 4.5 μm sections were deparaffinized and washed in phosphate-buffered saline (PBS). Antigen retrieval was performed by heating the sections in 10 mM sodium citrate buffer (pH = 6.0), followed by incubation with 3% hydrogen peroxide for 10 min and blocking with 1% bovine serum albumin (BSA)/ 5% goat serum for 1 h at room temperature. Sections were then incubated with primary antibodies (listed in Supplemental Table 2) overnight at 4°C and subsequently incubated with horseradish peroxidase (HRP)-conjugated secondary antibodies for 1 h at room temperature in the dark, followed by application of 3,3'-diaminobenzidine (DAB) substrate (Vector Laboratories). Images were captured using a Hamamatsu NanoZoomer scanner and analyzed with NDP.view2 and ImageJ software.

Immunofluorescence

For immunofluorescent staining of mouse skin and paw tissue, 4.5 μm paraffin sections were deparaffinized, antigen retrieved, and blocked as described above. Human skin tissue was embedded in optimal cutting temperature (OCT) and sectioned at 10 μm using a cryostat (Leica). Cryosections were fixed in ice-cold acetone for 15 min, permeabilized with 0.1% Triton X-100 for 10 min, and blocked with 3% BSA and 5% goat serum for 1 h at room temperature. Subsequently, sections were incubated with primary antibodies (listed in Supplemental Table 2) overnight at 4°C, and then incubated with corresponding Alexa 488/555-conjugated secondary antibodies (1:1000, Thermo) for 2 h at room temperature in the dark, followed by counterstaining with 4',6-diamidino-2-phenylindole (DAPI) (Roche) for 15 min. After washing in PBS, the sections were mounted with an antifade mounting medium (Solarbio). Images were captured using LAS X software (Leica) on a fluorescence microscope (Leica DM5500B) or a confocal microscope (Leica SP8).

TRAP staining

4.5 μm paraffin paw/digit sections or RAW 264.7 cells after induction of osteoclast differentiation (4% PFA fixed for 15 min) were stained with TRAP using TRAP kit (Sigma) according to the manufacturer's protocol by incubation for 1 h in a 37°C water bath protected from light. Images were captured using a Hamamatsu NanoZoomer scanner or a light microscope (OLYMPUS CKX31). The number of TRAP-positive, multinucleated (≥3 nuclei) osteoclasts was quantified.

Safranin O-Fast green staining

4.5 μm paraffin paw/digit sections were stained with safranin O/fast green using the modified Safranin O and Fast Green Stain Kit (For Bone) (Solarbio) according to the manufacturer's protocol. Cartilage is stained orange to red, nuclei are stained black, and the background is stained bluish-green. Images were captured using a Hamamatsu NanoZoomer scanner and NDP.view2 software. Safranin O intensity (red) was quantified using ImageJ software.

ELISA and Luminex Multi-Analyte Assays

Mouse tissue lysates were prepared as described above. Mouse plasma was collected after centrifugation of blood from the retro-orbital plexuses for 15 min at 3000 x rpm at 4°C. Human serum samples were collected from healthy individuals and patients with psoriasis. For cell supernatants, when the cell confluence reached 80%, the medium was changed with or without corresponding stimulations, and the supernatant was collected after 24 h. Protein concentrations were quantified using the Pierce BCA Protein Assay Kit (Thermo Fisher Scientific). ELISA kits and the R&D Luminex Multi-Analyte Assay (LXSAMSM) were used to determine the concentration of cytokines in different samples according to the manufacturer's instructions. All detailed product information is listed in Supplemental Table 2.

RNA extraction and real-time PCR

Total RNA was isolated from tissue and cells using TRIzol reagent (Takara), quantified using a NanoDrop spectrophotometer, and reverse transcribed using a reverse transcription reagent kit (Takara) according to the manufacturer's protocol. Real-time quantitative PCR was performed using SYBR Green Master Mix (ROX) (Roche) on an Applied Biosystems 7500 system. Primers were purchased from Tsingke Biotechnology Co., Ltd. (Beijing, China), sequences are listed in Supplemental Table 3. Each sample was run in triplicate, and the relative quantification of gene expression was normalized to β-actin levels and analyzed using qBase Plus software (Bio-Rad).

Single-cell RNA sequencing and data analysis

The initial data were processed using Cell Ranger (version 6.1.2). Subsequent data analysis followed the Seurat pipeline using the "Seurat" R package (version 4.3.0.1) (4). In the secondary filtering process conducted with Seurat, a screening protocol was implemented to identify and exclude low-quality cells and genes. Cells with fewer than 500 unique molecular identifiers (UMIs) or with over 15% of transcripts derived from mitochondria were considered low-quality cells and were discarded. For the calculation of principal components, the following criterion was adopted: (1) their collective contribution to cumulative variance exceeded 90%, (2) the individual contributions to total variance remaining were less than 5%, and (3) the variance discrepancy between consecutive components were less than 0.1%. These components were subsequently used for anchor selection and data integration, further dimensionality reduction, and clustering. Cell annotation was performed using a manual annotation approach. Data

visualization was performed using the "ggplot2" R package (version 3.4.3). KEGG enrichment analysis was performed using the "enrichKEGG" function of the "ClusterProfiler" R package (version 4.8.3)(5), specifically targeting differentially expressed genes (pvalueCutoff = 0.05). Gene Set Enrichment Analysis (GSEA) was performed using the "GSEA" function. To facilitate the computation of the GSVA, gene expression data was extracted from the Seurat object using the "GetAssayData" function. The "msigdbr" package in R (version 7.5.1) was used to extract hallmark gene sets. GSVA scores were calculated using the "GSVA" R package (version 1.48.3)(6), with the "gsva" function used to determine enrichment scores for individual cells. Subsequent differential analysis between groups was performed using the "limma" R package (version 3.56.2)(7). Visualization of pathways in dimensionality-reduced spaces was performed using the "FeaturePlot" function of the "Seurat" R package (version 4.3.0.1). LC-Biotechnology Co., Ltd. (Hangzhou, China) performed the single-cell RNA sequencing and assisted in data analysis.

RNA interference

Small interfering RNA (siRNA) targeting *SPRY1* or *Cd14* and negative control siRNA (NC) were purchased from Genomeditech Co., Ltd. (Shanghai, China). For *SPRY1* knockdown, NHEKs were transfected with *SPRY1* siRNA (sense: CCGUCAGAGAUUAGACUAUG; anti-sense: UCAUAGUCUAAUCUCUGACGG) or NC siRNA at a concentration of 50 nM using Lipofectamine 3000 transfection reagent (Thermo Fisher Scientific). For *Cd14* knockdown, RAW264.7 cells were

transfected with *Cd14* siRNA (sense: GGCUGAAGCAGGUACCUAA; anti-sense: UUAGGUACCUGCUUCAGCC) or NC siRNA at a concentration of 50 nM using Cell Available Lipid Nanoparticles (CALNP™) RNAi transfection reagent (D-Nano Therapeutics, Beijing, China).

Immunoblotting

Cells and tissue were lysed in RIPA buffer (Beyotime) supplemented with protease and phosphatase inhibitor cocktail (Thermo Fisher Scientific). Protein lysates were centrifuged at 12,000 × g for 15 min and supernatants were collected for concentration determination with the Pierce BCA Protein Assay Kit (Thermo Fisher Scientific). Equivalent amounts of protein were separated on 7.5% or 10% SDS–PAGE and transferred to NC membranes (Millipore). Then the membranes were blocked with blocking buffer for 1 h, and incubated with primary antibodies (the detailed antibody information is listed in Supplemental Table 2) at 4°C overnight. Membranes were washed with Tris-buffered saline with Tween 20 (TBST) buffer and further incubated with corresponding HRP-conjugated secondary antibodies at room temperature for 2 h. Blots were detected using ECL substrate (Thermo Fisher Scientific) and the ChemiDoc MP Imaging System (Bio-Rad) or the Amersham ImageQuant 800 Western blot imaging system (Cytiva).

Supplemental Figures and Figure Legends

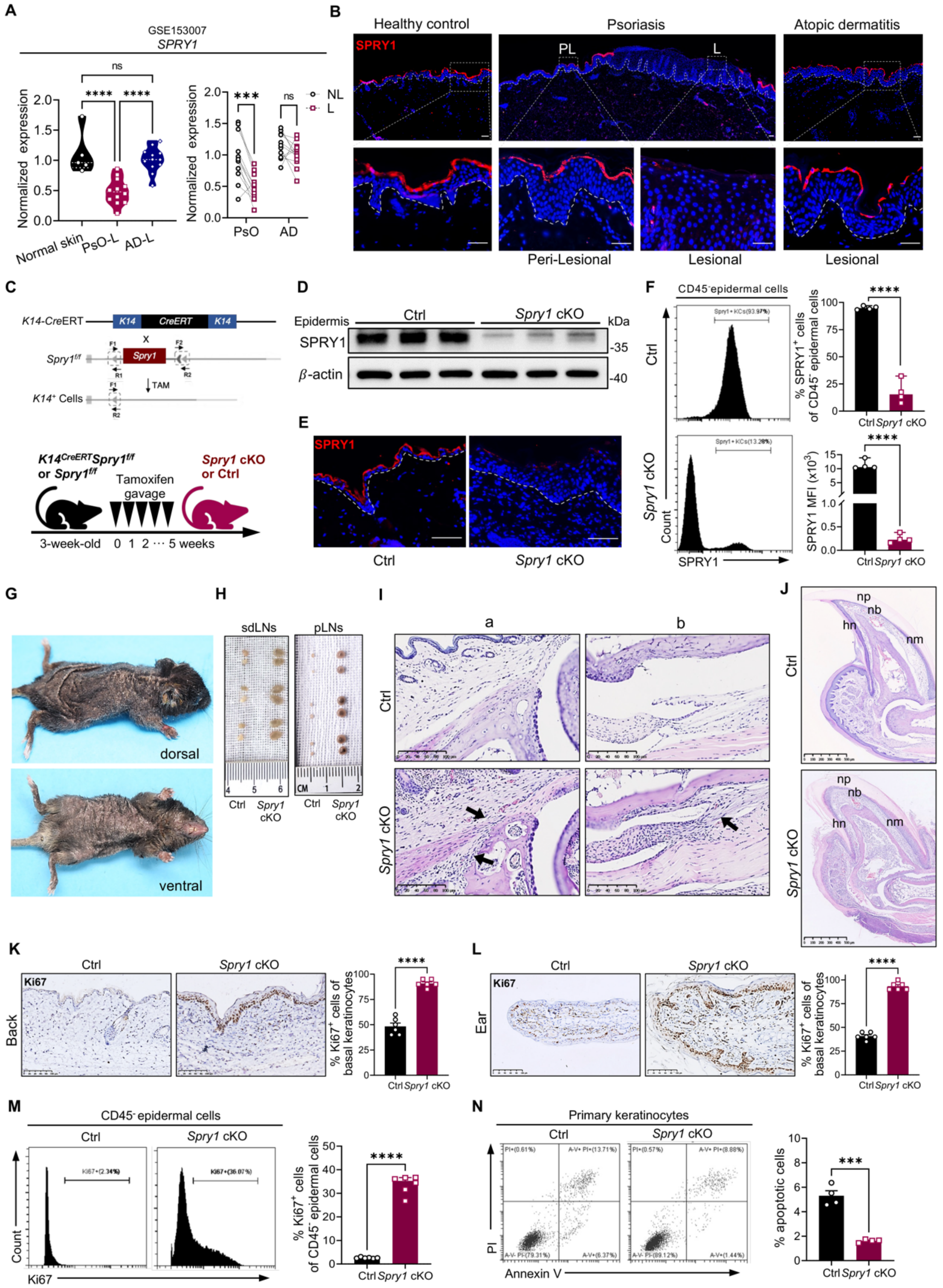

Supplemental Figure 1. SPRY1 deficiency in epidermal keratinocytes is a signature of psoriasis. (**A**) *SPRY1* gene expression in lesional and non-lesional skin from patients with psoriasis or atopic dermatitis (AD), and normal skin from the GEO database (GSE153007). Left, comparison between normal skin, lesional skin of psoriasis, and lesional skin of AD; right, comparison between lesional and non-lesional skin of psoriasis or AD. (**B**) Immunofluorescence staining of SPRY1 in the skin of psoriasis (lesion and peri-lesion), AD

(leison), and healthy control. Boxed areas are magnified below. Scale bar, 50 μm. (**C**) Scheme of SPRY1 deletion in *K14*^{CreERT} *Spry1*$^{f/f}$ mice after tamoxifen gavage for 5 consecutive weeks (0.1 mg/g body weight per day, 4 days per week). (**D**) Immunoblotting analysis of SPRY1 expression in the epidermis of control and *Spry1* cKO mice (n = 3). (**E**) Immunofluorescence staining of SPRY1 in the skin of control and *Spry1* cKO mice. (**F**) Flow cytometric histograms (left) and quantification (right) of SPRY1^{+} cells and SPRY1 MFI in CD45^{-} epidermal cells from control and *Spry1* cKO mice (n = 4). (**G**) Dorsal and ventral views of a *Spry1* cKO mouse of the indicated genotypes. (**H**) Macroscopic views of skin-draining lymph nodes (sdLNs) and popliteal lymph nodes (pLNs) in control and *Spry1* cKO mice (n = 3). (**I**) Magnified views of (**a**) and (**b**) in **Figure 1H**. Black arrows indicate enthesitis. Scale bar, 100 μm. (**J**) Representative H&E staining of the nails from paws of control and *Spry1* cKO mice. Scale bar, 500 μm. Nail plate (np), nail matrix (nm), nail bed (nb), and hyponychium (hn). (**K** and **L**) Immunohistochemical staining (left) and quantification (right) of Ki67^{+} cells in the back skin (**K**) and ears (**L**) of control and *Spry1* cKO mice (n = 6). (**M**) Flow cytometric histograms (left) and quantification (right) of Ki67^{+} cells in CD45^{-} epidermal cells from control and *Spry1* cKO mice (n = 6). (**N**) Flow cytometric plots (left) and quantification (right) of annexin V^{+}PI^{-} apoptotic cells in primary mouse keratinocytes from control and *Spry1* cKO mice (n = 4). Error bars indicate mean ± SEM. P values were determined using two-tailed unpaired Student's t-test (**F** and **K-N**), two-tailed paired Student's t-test (the right panel of **A**), and One-way ANOVA (the left panel of **A**).

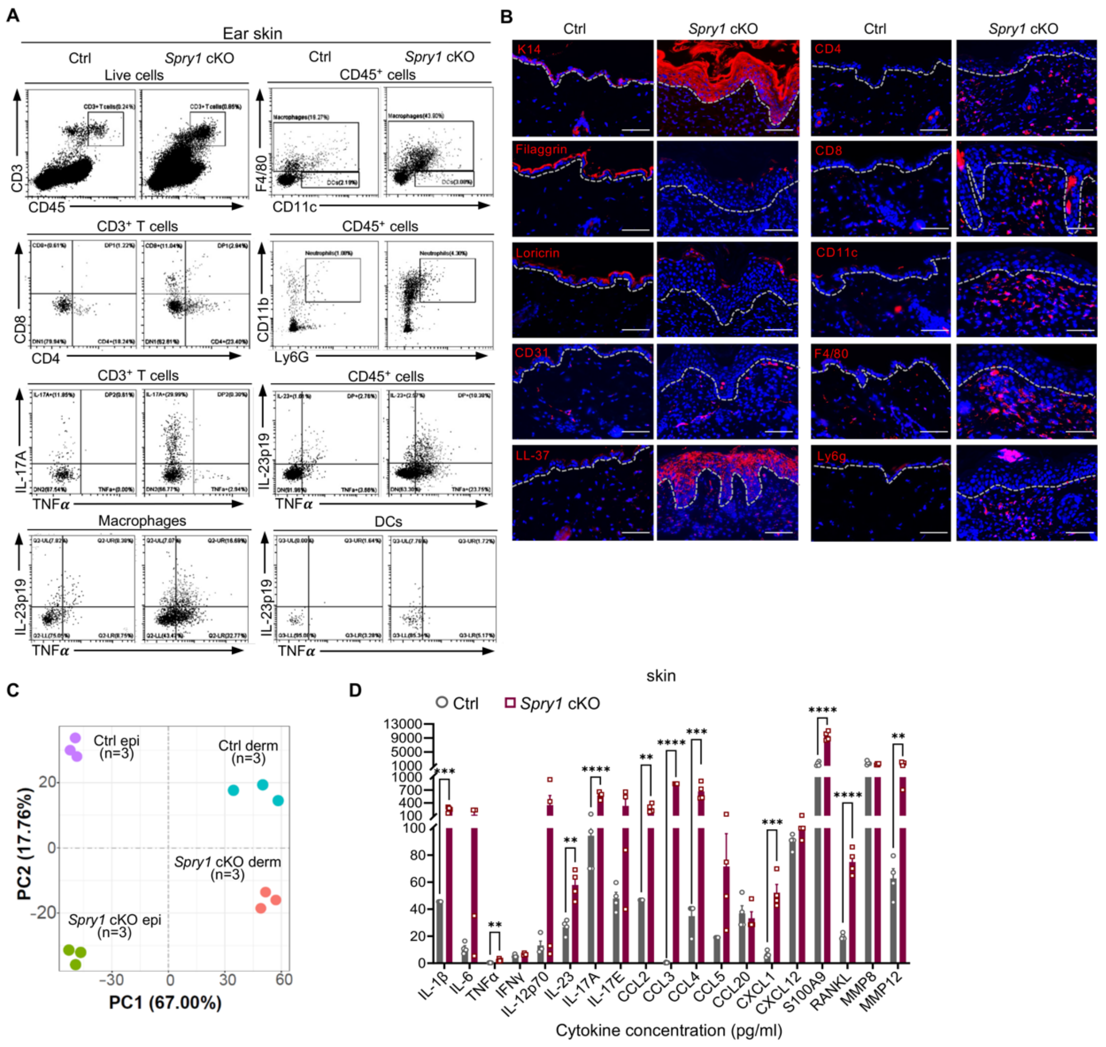

Supplemental Figure 2. Skin inflammation in *Spry1* cKO mice shows features consistent with psoriasis. (**A**) Flow cytometric plots of different immune cell subsets in the ear skin from control and *Spry1* cKO mice (n = 4). (**B**) Immunofluorescence staining of psoriasis-associated epidermal differentiation and inflammatory infiltration markers in the skin of control and *Spry1* cKO mice. Dashed lines demarcate the epidermis/dermis boundary. Scale bar, 50 μm. (**C**) Principal component analysis (PCA) plots of bulk RNA-seq data from the epidermis and dermis of control and *Spry1* cKO mice (n = 3). (**D**) Quantification of selected cytokines in the ear skin from control and *Spry1* cKO mice detected by Luminex assays (n = 4). Error bars indicate mean ± SEM. P values were determined using two-tailed unpaired Student's t-test (**D**).

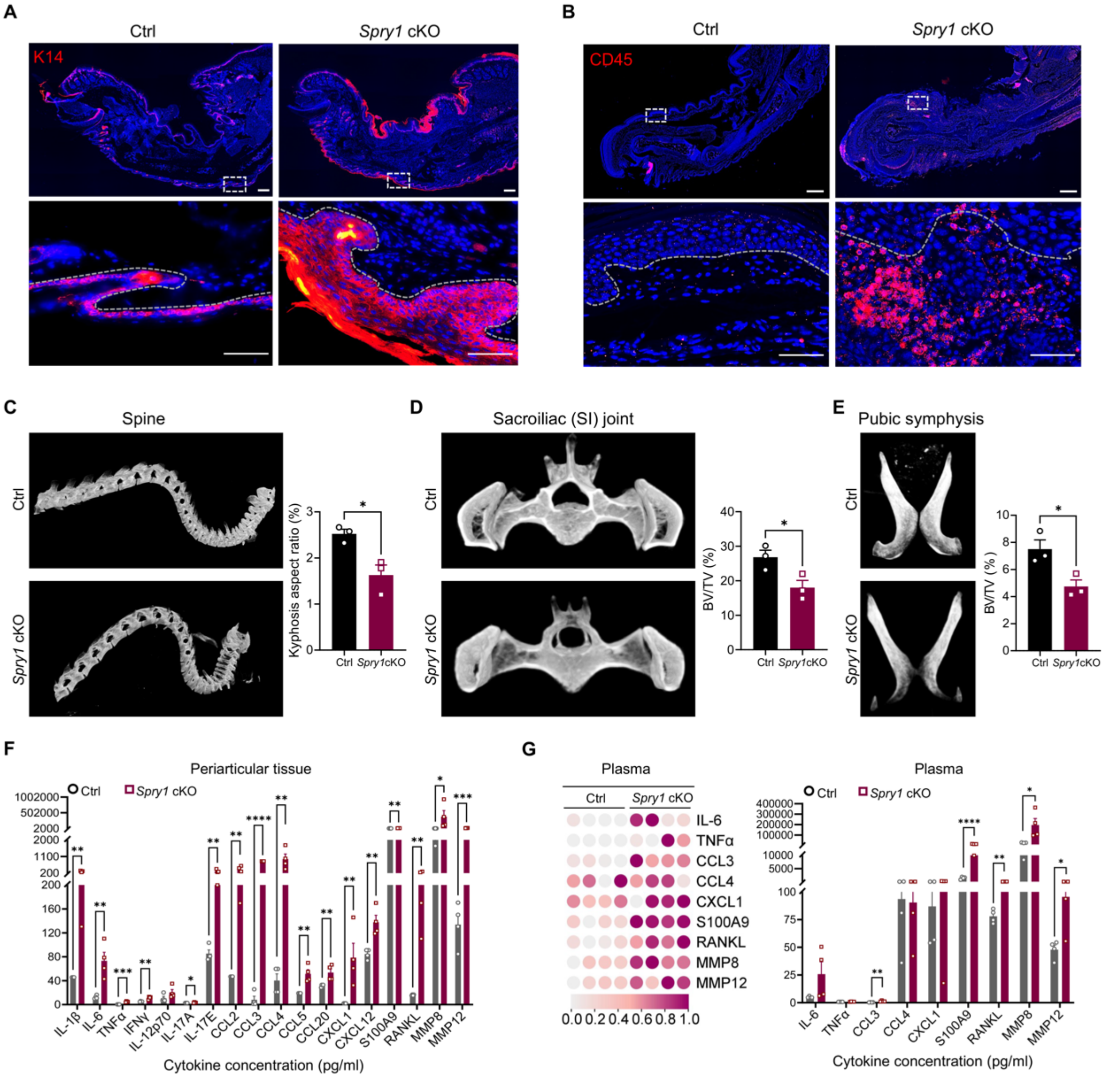

Supplemental Figure 3. Joint disease in *Spry1* cKO mice shows features consistent with psoriatic arthritis. (**A** and **B**) Immunofluorescence staining of K14 (**A**) and CD45 (**B**) in the digits of paws from control and *Spry1* cKO mice. Boxed areas are magnified below; dashed lines demarcate the epidermis/dermis boundary. Scale bar, 50 µm. (**C-E**) Micro-CT images (left) and quantification (right) of cervicothoracic kyphosis (spine) (**C**), sacroiliac (SI) joint (**D**), and pubic symphysis (**E**) in control and *Spry1* cKO mice (n = 3). (**F**) Quantification of selected cytokines in the periarticular tissue from control and *Spry1* cKO mice detected by Luminex assays (n = 4). (**G**) Heatmap (left) and quantification (right) of selected cytokines in the plasma from control and *Spry1* cKO mice detected by Luminex assays (n = 4). Error bars indicate mean ± SEM. P values were determined using two-tailed unpaired Student's t-test (**C-G**).

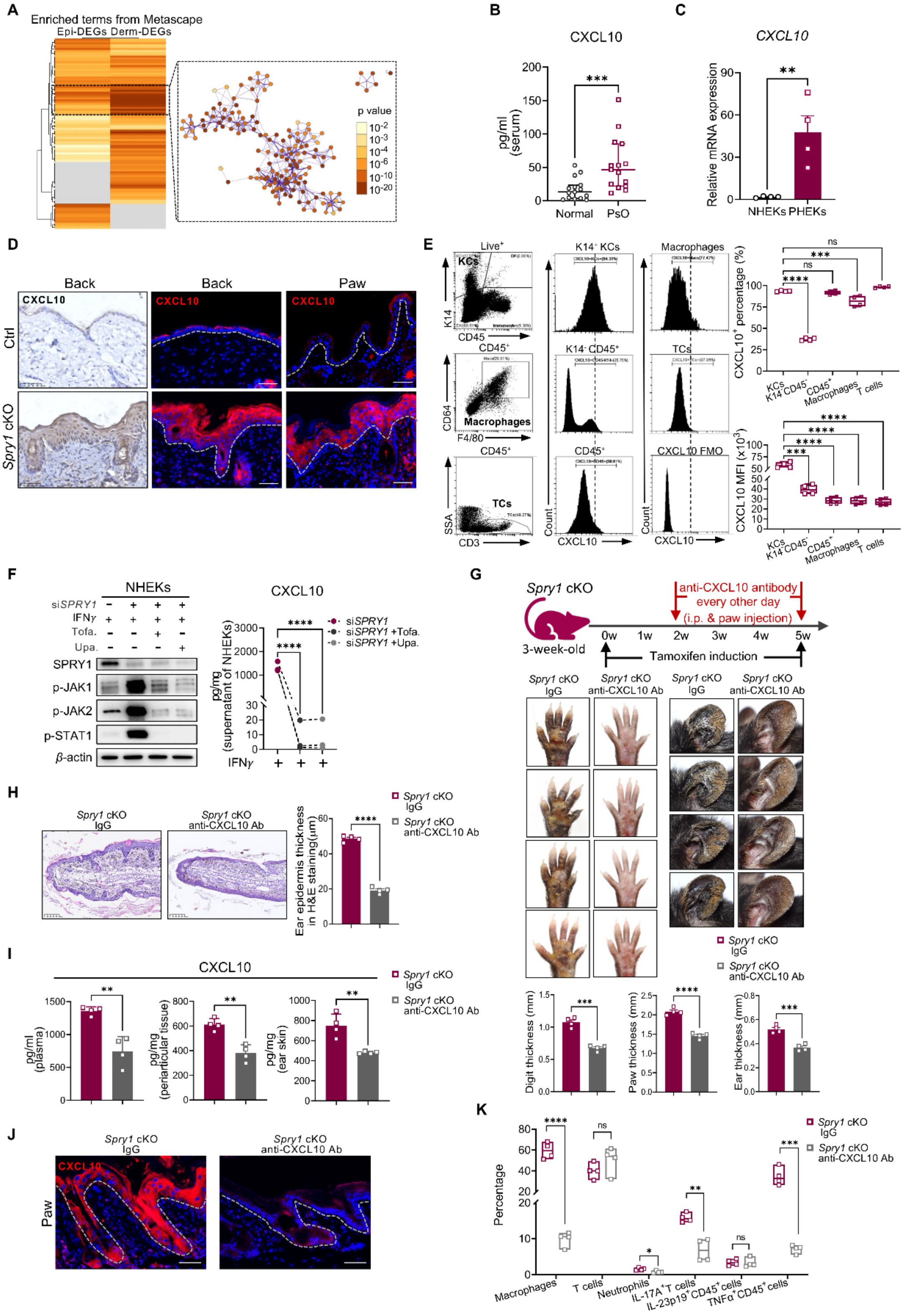

Supplemental Figure 4. Keratinocyte-derived CXCL10 plays a crucial role in triggering psoriatic arthritis-like inflammation. (**A**) Heatmap (left) shows the top 100 commonly enriched terms of all epidermal DEGs and all dermal DEGs (*Spry1* cKO vs control) ($p < 0.01$); the network (right) shows terms with the best p-values of both sides. Each term is colored by p-value. (**B**) ELISA quantification of CXCL10 in the serum of healthy controls and patients

with psoriasis (n=15). (**C**) Relative mRNA expression of *CXCL10* in the primary normal human epidermal keratinocytes (NHEKs) and psoriatic human epidermal keratinocytes (PHEKs) (n = 4). (**D**) Immunohistochemical (left) and immunofluorescence (middle and right) staining of CXCL10 in the skin of back and paw from control and *Spry1* cKO mice. Dashed lines demarcate the epidermis/dermis boundary. Scale bar, 50 μm. (**E**) Flow cytometric analysis of CXCL10 expression in skin cells from *Spry1* cKO mice after stimulation with 50ng/mL recombinant mouse IFNγ and BFA for 6h. Representative plots (left) and quantification (right) of CXCL10$^+$ cell percentages and CXCL10 MFI in CD45$^-$K14$^+$ keratinocytes (KCs), CD45$^-$K14$^-$ cells (including fibroblasts and endothelial cells), total CD45$^+$ cells, macrophages, and T cells (n = 4). (**F**) Immunoblotting analysis of protein levels associated with JAK1/2-STAT1 pathway in NHEKs with NC or si*SPRY1* treatment followed by incubation with 50ng/mL recombinant human IFNγ together with 10 μM Tofacitinib or Upadacitinib for 24h. Right, ELISA quantification of CXCL10 in the supernatant of NHEKs (n = 3). (**G**) Scheme of the treatment with anti-CXCL10 antibody or isotype antibody (IgG) for age- and sex-matched *Spry1* cKO mice, and macroscopic views of the paw and ear of each mouse from each group. Lower panels show quantification of digit, paw, and ear thickness (n = 4). (**H**) Representative H&E staining of the ears from *Spry1* cKO mice treated with anti-CXCL10 antibody and IgG. Right, quantification of ear epidermis thickness (n = 4). Scale bar, 100 μm. (**I**) ELISA quantification of CXCL10 in the plasma, periarticular tissue, and ears from *Spry1* cKO mice treated with anti-CXCL10 antibody and IgG (n = 4). (**J**) Immunofluorescence staining of CXCL10 in the paw skin from *Spry1* cKO mice treated with anti-CXCL10 antibody and IgG. Dashed lines demarcate the epidermis/dermis boundary. Scale bar, 50 μm. (**K**) Percentages of different immune cell subsets in the periarticular tissue from *Spry1* cKO mice treated with anti-CXCL10 antibody and IgG analyzed by flow cytometry (n = 4). Error bars indicate mean ± SEM. P values were determined using two-tailed unpaired Student's t-test (**C, D, G-I,** and **K**) and One-way ANOVA (**B** and **F**).

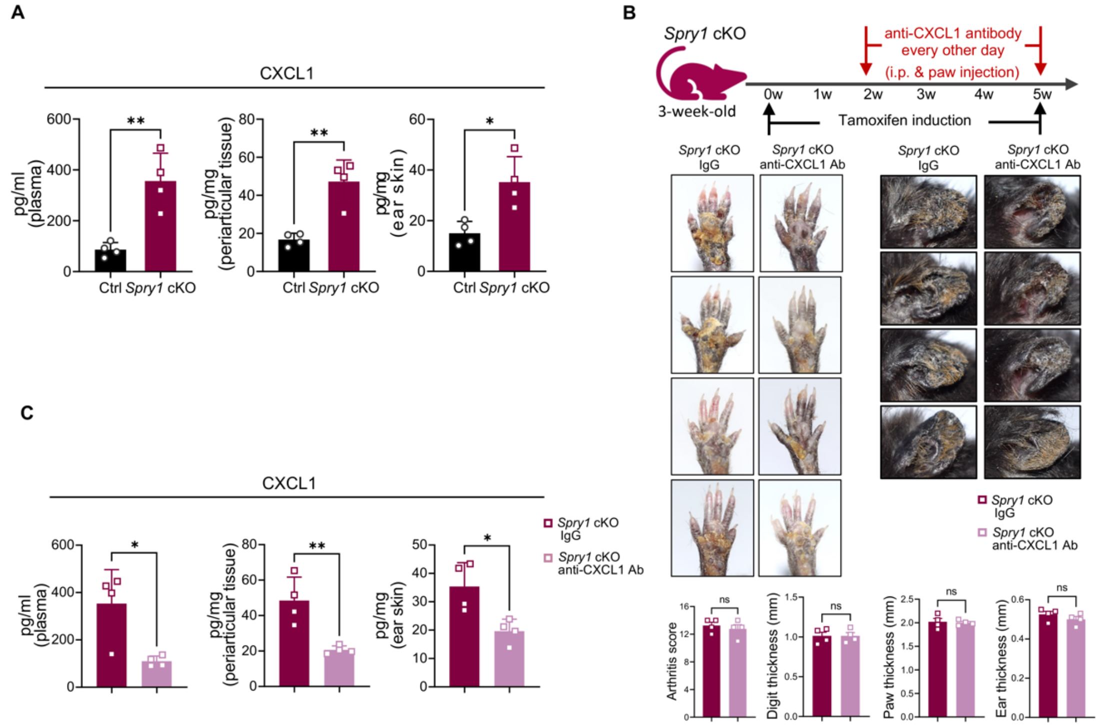

Supplemental Figure 5. CXCL1 does not play a central role in triggering psoriatic arthritis-like inflammation. (**A**) ELISA quantification of CXCL1 in the plasma, periarticular tissue, and ears from control and *Spry1* cKO mice (n = 4). (**B**) Scheme of the treatment with anti-CXCL1 antibody or isotype antibody (IgG) for age- and sex-matched *Spry1* cKO mice, and macroscopic views of the paw and ear of each mouse from each group. Lower panels show quantification of arthritis scores, and thickness of the digit, paw, and ear (n = 4). (**C**) ELISA quantification of CXCL1 in the plasma, periarticular tissue, and ears from *Spry1* cKO mice treated with anti-CXCL1 antibody and IgG (n = 4). P values were determined using two-tailed unpaired Student's t-test (**A-C**).

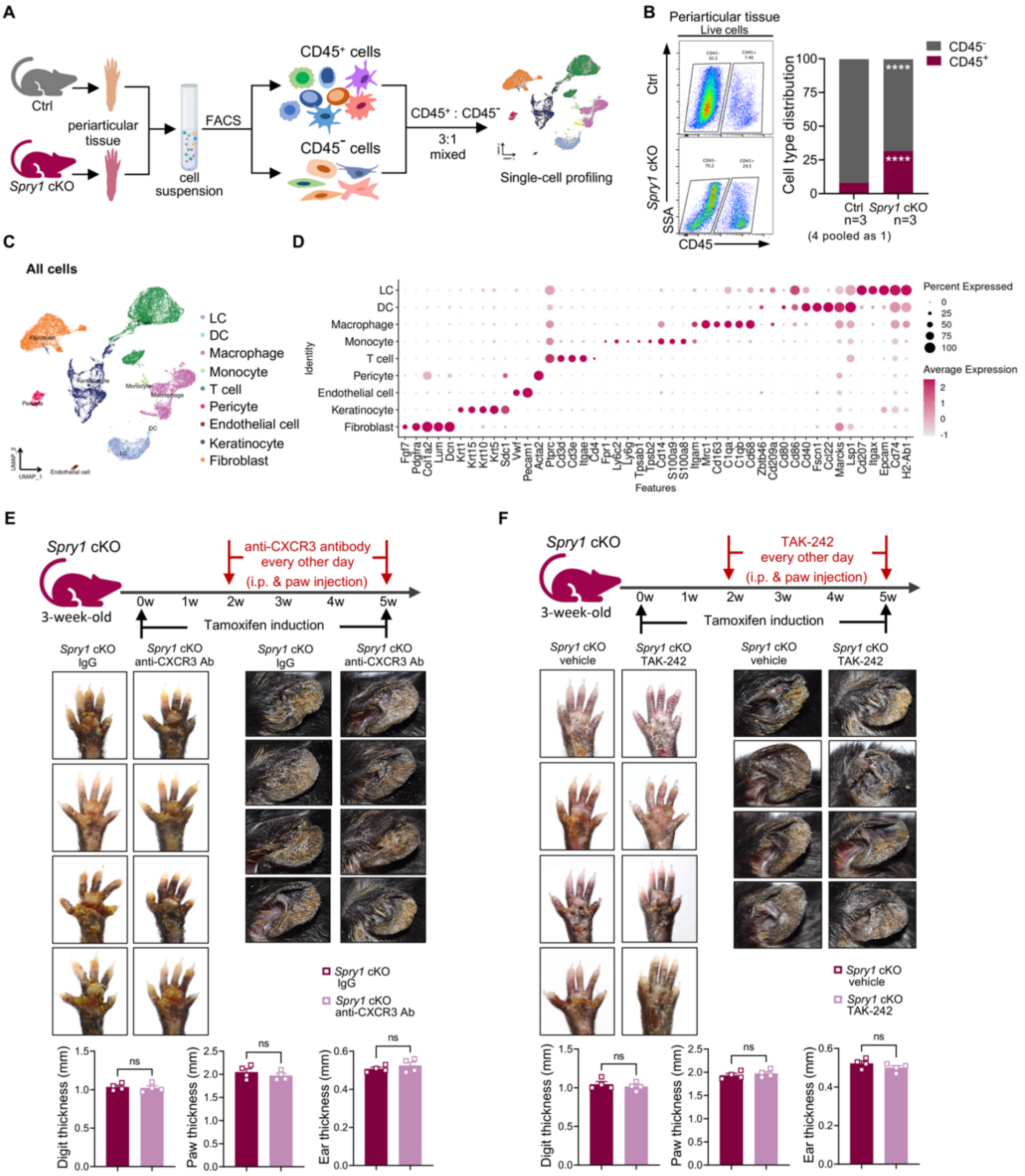

Supplemental Figure 6. The pathogenic role of keratinocyte-derived CXCL10 in psoriatic arthritis-like inflammation is independent of CXCR3 and TLR4. (**A**) Scheme of the experimental design for scRNA-Seq of periarticular tissue from control and *Spry1* cKO mice (n = 3). CD45$^+$ immune cells and CD45$^-$ non-immune cells were sorted by fluorescence-activated cell sorting (FACS) and then mixed at a ratio of 3:1 to generate single-cell transcriptomic profiles. To obtain a sufficient amount of cells, 4 control mice were pooled as 1 mouse. (**B**) Flow cytometric plots (left) and frequencies (right) of CD45$^+$ immune cells in the periarticular tissue from control and *Spry1* cKO mice using FACS applied in (**A**). (**C**) UMAP plots of all periarticular cells (immune and non-immune cells) from control and *Spry1* cKO mice by scRNA-Seq, showing 9 clusters (n = 3). (**D**) Dot plots showing the scaled expression of selected marker genes for all cell types defined in (**C**). (**E** and **F**) Scheme of the treatment with anti-CXCR3 antibody or isotype antibody (IgG) (**E**), and the treatment with TAK-242 or vehicle (**F**) for age- and sex-matched *Spry1* cKO mice, and macroscopic views of the paw and

ear of each mouse from each group. Lower panels show quantification of arthritis scores, and thickness of digit, paw, and ear (n = 4). Error bars indicate mean ± SEM. P values were determined using two-tailed unpaired Student's t-test (**B, E**, and **F**).

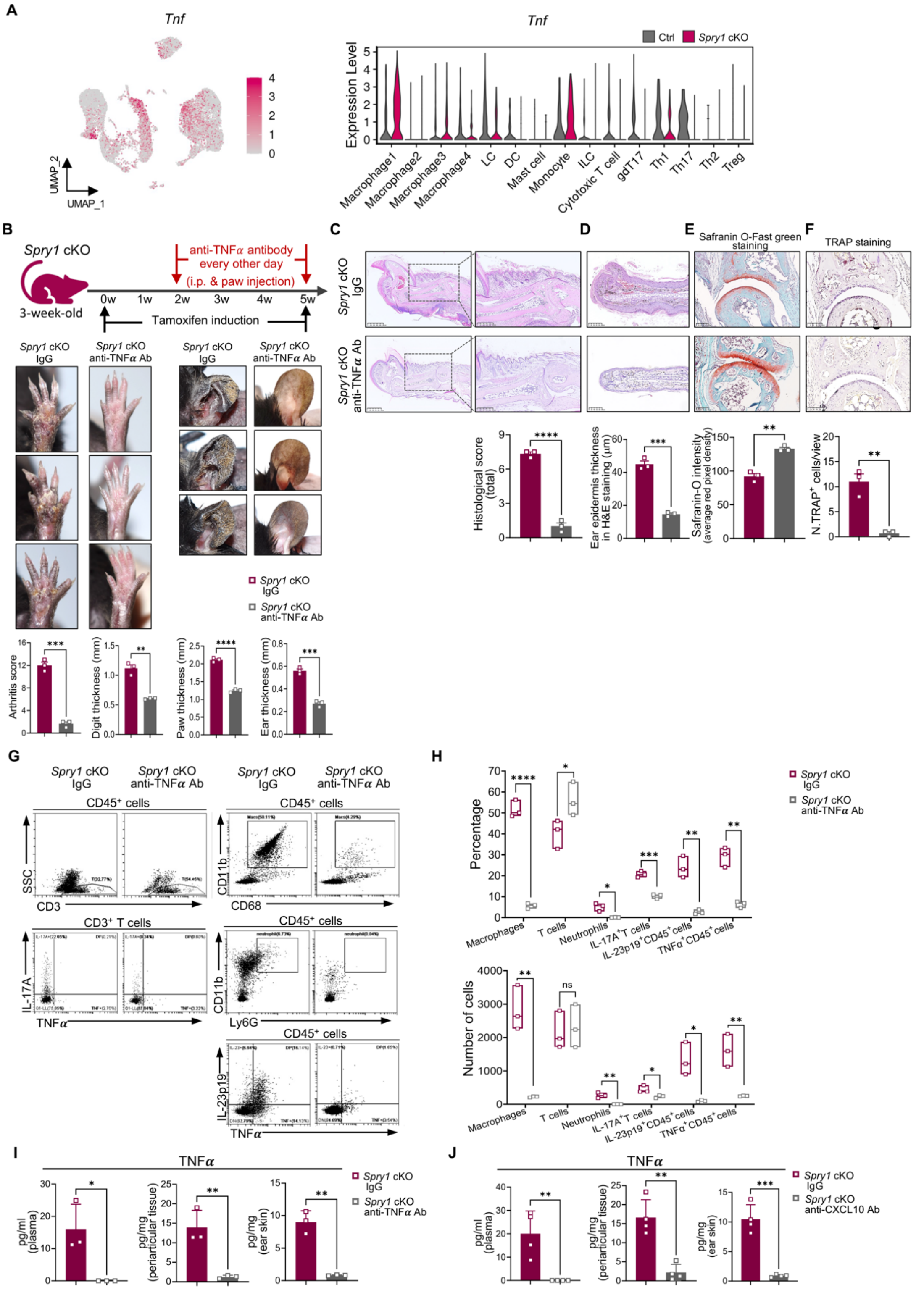

Supplemental Figure 7. TNF*α* is a key pathogenic downstream mediator in psoriatic arthritis-like inflammation. (**A**) UMAP plots (left) and violin plots (right) showing *Tnf* expression in all immune cell types of the periarticular tissue from control and *Spry1* cKO mice defined in **Figure 4, B** and **C**. (**B**) Scheme of the treatment with anti-TNF*α* antibody or isotype antibody (IgG) for age- and sex-matched *Spry1* cKO mice, and macroscopic views of the paw and ear of each mouse from each group. Lower panels show quantification of arthritis scores,

and thickness of the digit, paw, and ear (n = 3). (**C-F**) Representative H&E staining of the paws (left scale bar, 500 μm; right scale bar, 250 μm) (**C**), H&E staining of the ears (scale bar, 100 μm) (**D**), Safranin O-Fast green staining (scale bar, 100 μm) (**E**) and TRAP staining (scale bar, 100 μm) (**F**) of the paws from *Spry1* cKO mice treated with anti-TNFα antibody and IgG. Lower panels show quantification of total histological scores, ear epidermis thickness, Safranin-O intensity, and TRAP^{+} osteoclasts respectively (n = 3). (**G** and **H**) Flow cytometric plots (**G**) and quantification (**H**) of different immune cell subsets in the periarticular tissue from *Spry1* cKO mice treated with anti-TNFα antibody and IgG (n = 3). (**I** and **J**) ELISA quantification of TNFα in the plasma, periarticular tissue, and ears from *Spry1* cKO mice treated with anti-TNFα antibody and IgG (n = 3) (**I**), and from *Spry1* cKO mice treated with anti-CXCL10 antibody and IgG (n = 4) (**J**). Error bars indicate mean ± SEM. P values were determined using two-tailed unpaired Student's t-test (**B-F and H-J)**.

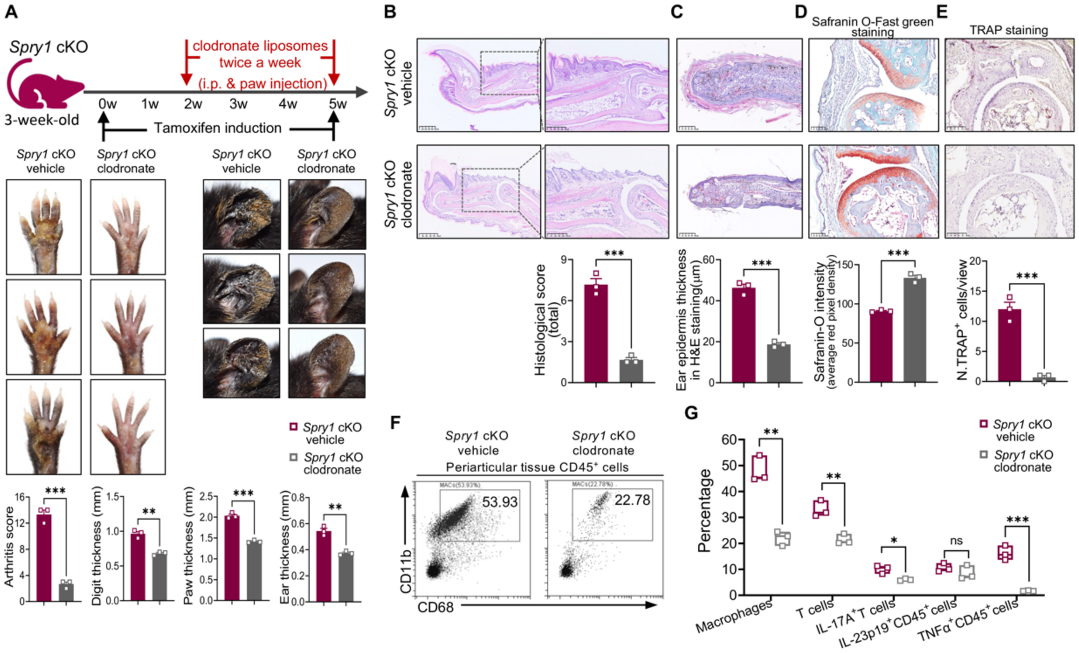

Supplemental Figure 8. Periarticular macrophages are essential for the development of psoriatic arthritis-like inflammation. (**A**) Scheme of the treatment with clodronate liposomes (for macrophage depletion) or vehicle for age- and sex-matched *Spry1* cKO mice, and macroscopic views of the paw and ear of each mouse from each group. Lower panels show quantification of arthritis scores, and thickness of the digit, paw, and ear (n = 3). (**B-E**) Representative H&E staining of the paws (left scale bar, 500 μm; right scale bar, 250 μm) (**B**), H&E staining of the ears (scale bar, 100 μm) (**C**), Safranin O-Fast green staining (scale bar, 100 μm) (**D**) and TRAP staining (scale bar, 100 μm) (**E**) of the paws from *Spry1* cKO mice treated with clodronate liposomes or vehicle. Lower panels show quantification of total histological scores, ear epidermis thickness, Safranin-O intensity, and $TRAP^+$ osteoclasts respectively (n = 3). (**F**) Flow cytometric plots of $CD11b^+CD68^+$ macrophages in the periarticular tissue from *Spry1* cKO mice treated with clodronate liposomes and vehicle. (**G**) Percentages of different immune cell subsets in the periarticular tissue from *Spry1* cKO mice treated with clodronate liposomes and vehicle analyzed by flow cytometry (n = 3). Error bars indicate mean ± SEM. P values were determined using two-tailed unpaired Student's t-test (**A-E**, and **G**).

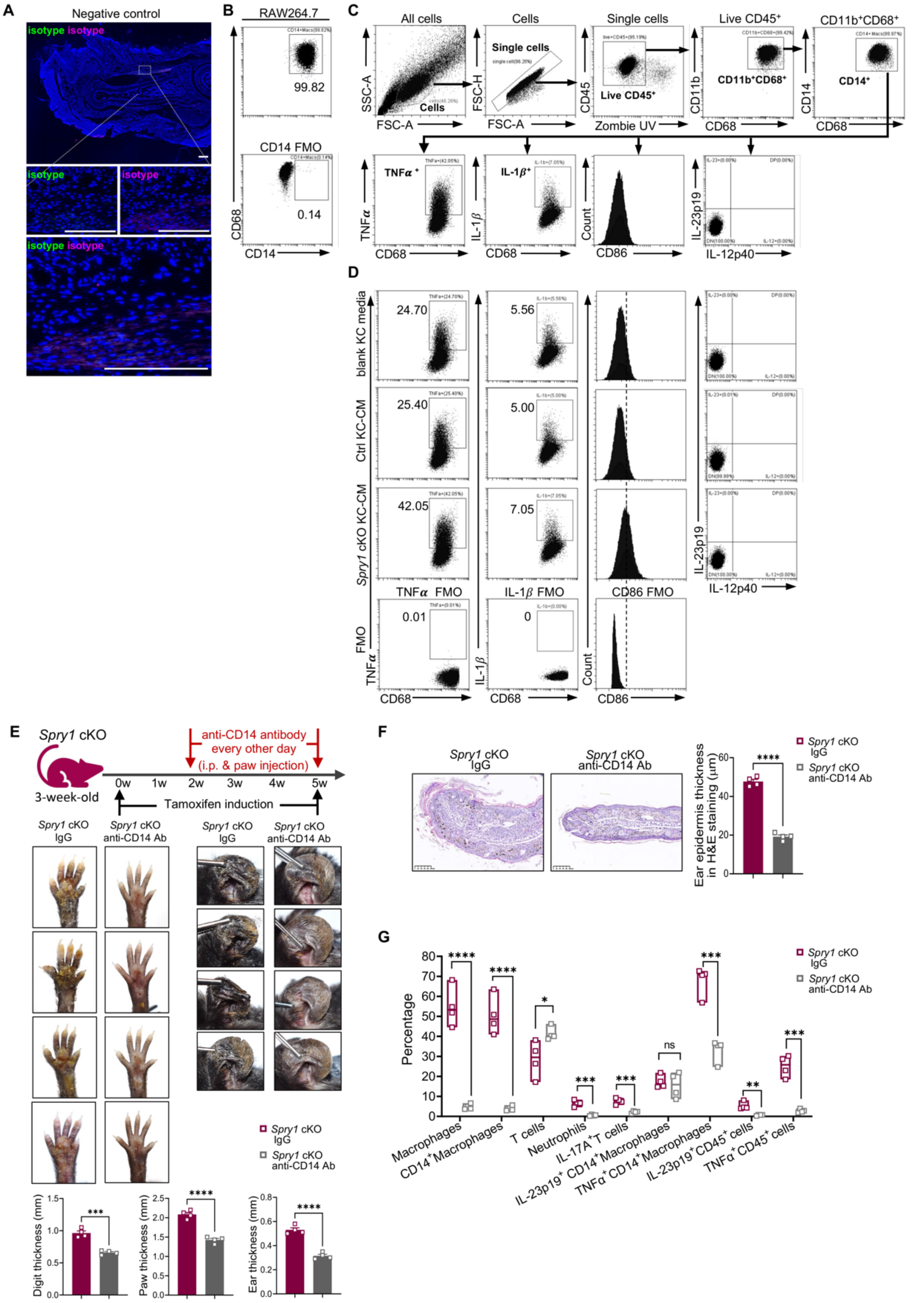

Supplemental Figure 9. Periarticular $CD14^{hi}$ macrophages play a crucial pro-inflammatory role in triggering psoriatic arthritis-like inflammation by producing TNF*α*. (**A**) Isotype controls for immunofluorescence staining of CD68 (green) and CD14 (magenta) in the digits from paws of *Spry1* cKO mice in **Figure 6A**. Boxed areas are magnified below. Scale bar, 50 μm. (**B**) Flow cytometric plots of CD68 and CD14 expression in RAW264.7 cells. (**C**) Gating strategy used for flow cytometric analysis of in vitro experiments with RAW264.7 cells.

(**D**) Flow cytometric plots of TNF***α***, IL-1*β,* CD86, IL-23p19, and IL-12p40 expression in RAW264.7 cells treated with blank keratinocyte medium, control KC-CM, and *Spry1* cKO KC-CM for 24h, followed by stimulation with 50ng/mL LPS and BFA for 6h (n = 3). (**E**) Scheme of the treatment with anti-CD14 antibody or isotype antibody (IgG) for age- and sex-matched *Spry1* cKO mice, and macroscopic views of the paw and ear of each mouse from each group. Lower panels show quantification of digit, paw, and ear thickness (n = 4). (**F**) Representative H&E staining of the ears from *Spry1* cKO mice treated with anti-CD14 antibody and IgG. The right panel shows ear epidermis thickness quantification (n = 4). Scale bar, 100 μm. (**G**) Percentages of different immune cell subsets in the periarticular tissue from *Spry1* cKO mice treated with anti-CD14 antibody and IgG analyzed by flow cytometry (n = 4). Error bars indicate mean ± SEM. P values were determined using two-tailed unpaired Student's t-test (**E-G**).

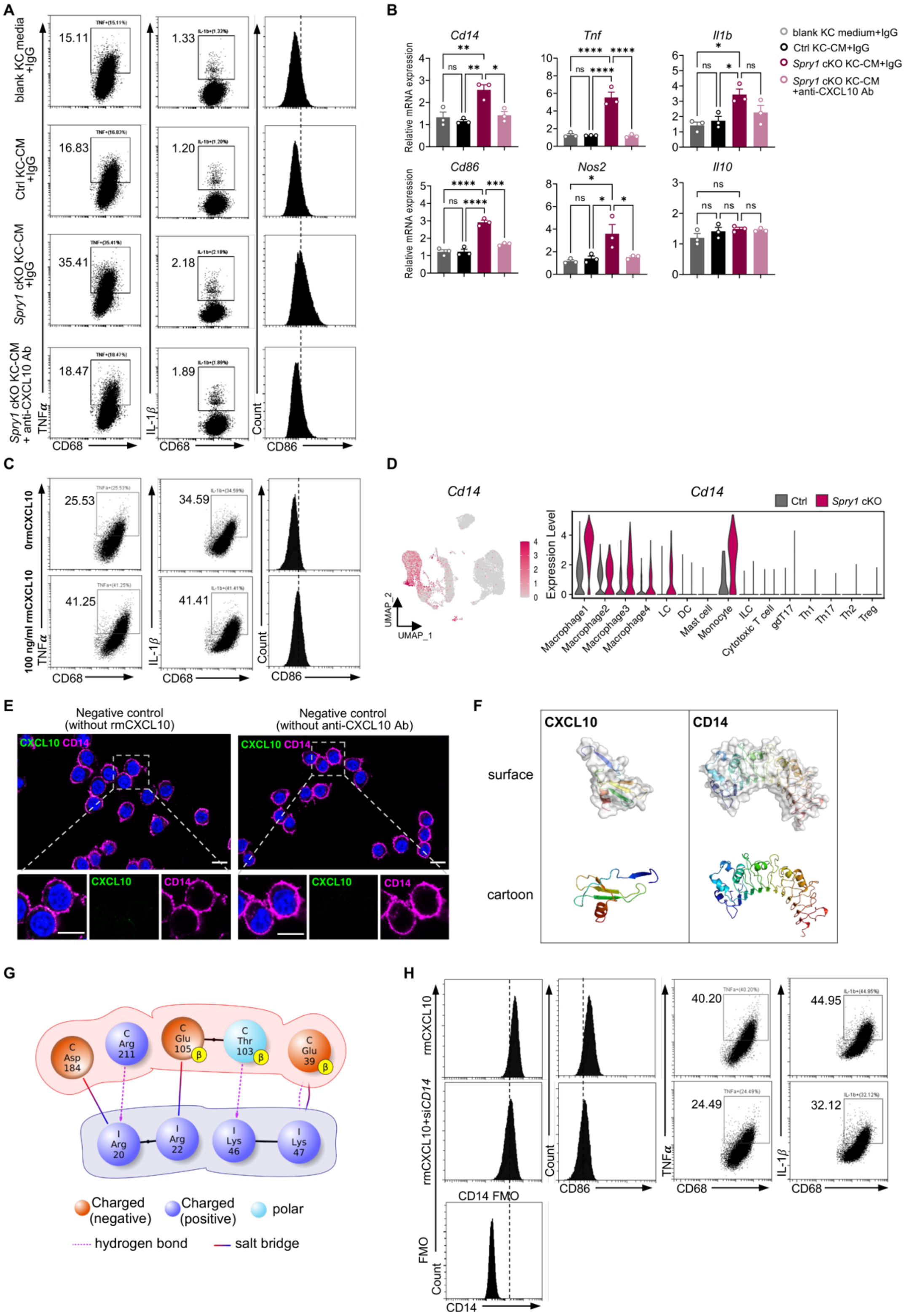

Supplemental Figure 10. CXCL10 binds to CD14 and mediates the pro-inflammatory response of periarticular CD14^{hi} macrophages.

(**A**) Flow cytometric plots of TNFα, IL-1β, and CD86 expression in RAW264.7 cells treated with blank keratinocyte medium+ IgG, control KC-CM+ IgG, *Spry1* cKO KC-CM+ IgG, and *Spry1* cKO KC-CM+ 2μg/mL anti-CXCL10 antibody for 24h, followed by incubation with

50ng/mL LPS and BFA for 6h (n = 3). (**B**) Relative mRNA expression of genes associated with macrophage activation and polarization in RAW264.7 cells treated with blank keratinocyte medium+ IgG, control KC-CM+ IgG, *Spry1* cKO KC-CM+ IgG, and *Spry1* cKO KC-CM+ 2μg/mL anti-CXCL10 antibody for 24h (n = 3). (**C**) Flow cytometric plots of TNF$\boldsymbol{\alpha}$, IL-1β, and CD86 expression in RAW264.7 cells treated with or without 100ng/mL recombinant murine CXCL10 for 24h, followed by stimulation with 50ng/mL LPS and BFA for 6h (n = 3). (**D**) UMAP plots (left) and violin plots (right) showing *Cd14* expression in all immune cell types of the periarticular tissue from control and *Spry1* cKO mice defined in **Figure 4, B** and **C**. (**E**) Negative controls for immunofluorescence staining of CXCL10 (without recombinant murine CXCL10 treatment or anti-CXCL10 antibody staining) in RAW264.7 cells in **Figure 7F**. Boxed areas are magnified below. Scale bar, 100 μm. (**F**) The 3D structures of CXCL10 and CD14 obtained from the Protein Data Bank (PDB). (**G**) 2D images showing key residue bindings between mouse CXCL10 and CD14. (**H)** Flow cytometric plots of CD14, CD86, TNF$\boldsymbol{\alpha}$, and IL-1β expression in RAW264.7 cells treated with NC or si*Cd14*, followed by stimulation with 100ng/mL recombinant murine CXCL10 for 24h and subsequent incubation with 50ng/mL LPS and BFA for 6h (n = 3). Error bars indicate mean ± SEM. P values were determined using One-way ANOVA (**B**).

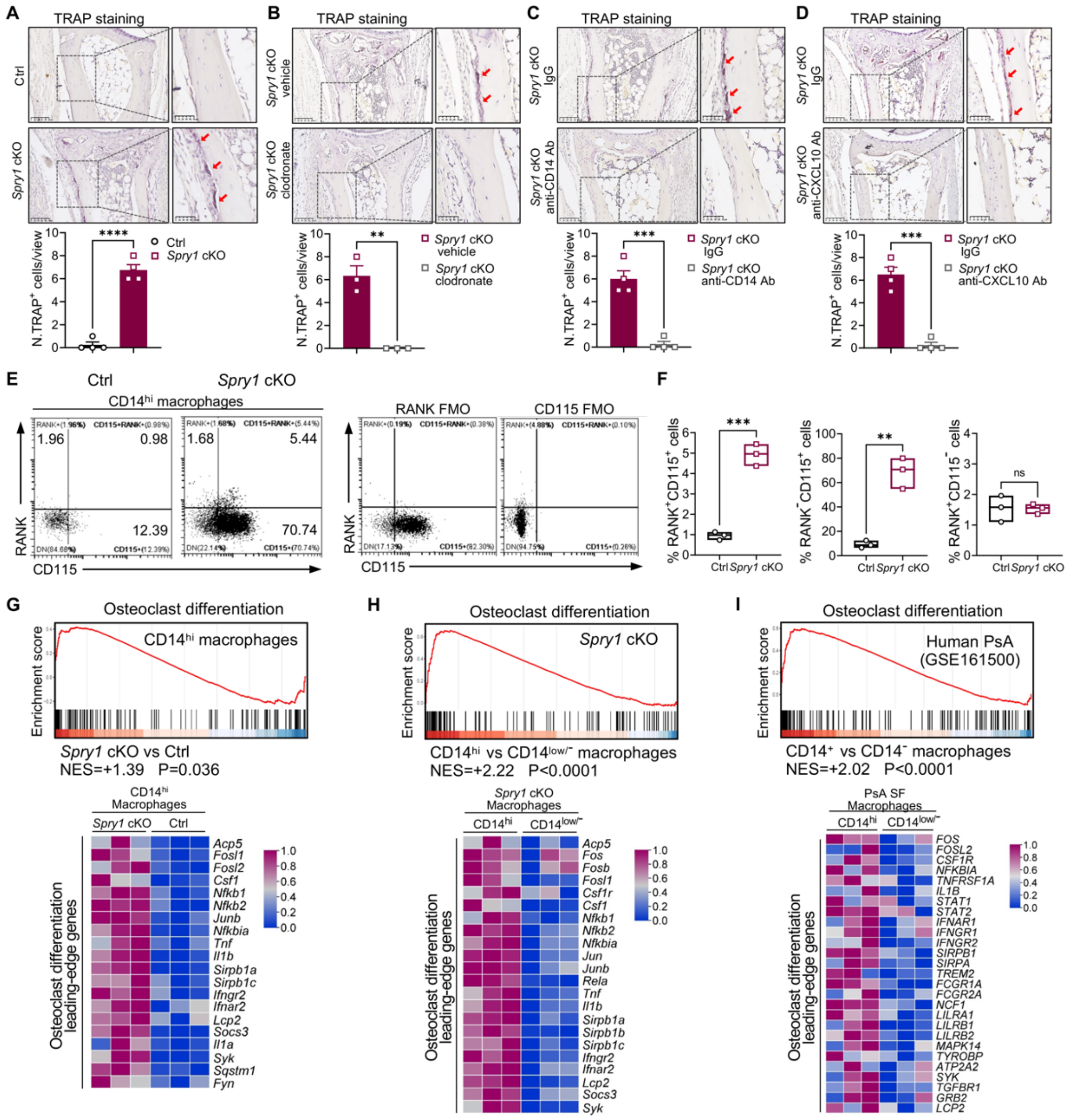

Supplemental Figure 11. Periarticular CD14^{hi} macrophages are predisposed to differentiate into osteoclasts. (**A-D**) TRAP staining of paws from control and *Spry1* cKO mice (n = 4) (**A**), *Spry1* cKO mice treated with clodronate liposomes and vehicle (n = 3) (**B**), *Spry1* cKO mice treated with anti-CD14 antibody and IgG (n = 4) (**C**), and *Spry1* cKO mice treated with anti-CXCL10 antibody and IgG (n = 4) (**D**). Scale bar, 100 μm; boxed areas are magnified on the right (scale bar, 50 μm); red arrows indicate TRAP^{+} cells. Lower panel shows the quantification of TRAP^{+} cells. (**E** and **F**) Flow cytometric analysis of RANK and CD115 (markers associated with osteoclast precursors) expression in periarticular CD14^{hi} macrophages from control and *Spry1* cKO mice (**E**), and frequencies (**F**) of RANK^{+}CD115^{+}, RANK^{-}CD115^{+}, and RANK^{-}CD115^{-} cells (n = 3). (**G-I**) GSEA of osteoclast differentiation pathway on genes of periarticular CD14^{hi} macrophages ranked by $\log_2$FC between *Spry1* cKO and control mice (scRNA-Seq) (**G**), on genes ranked by $\log_2$FC between CD14^{hi} and CD14$^{low/-}$ macrophages in periarticular tissue from *Spry1* cKO mice (scRNA-Seq) (**H**), on genes ranked by $\log_2$FC between CD14^{+} and CD14^{-} macrophages in synovial fluid from human PsA (scRNA-Seq, GSE161500) (**I**). The leading-edge genes are listed below. Error bars indicate mean ± SEM. P values were determined using two-tailed unpaired Student's t-test (**A-D**, and **F**).

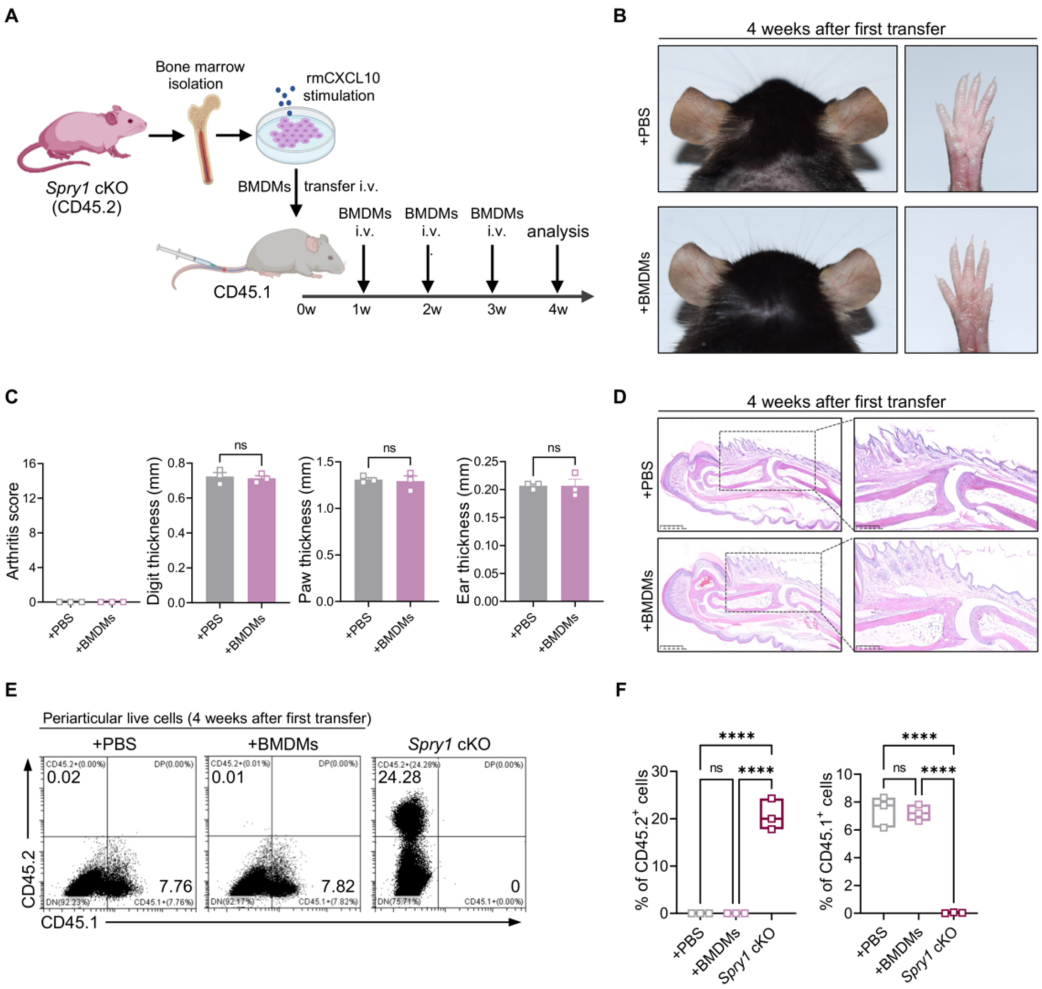

Supplemental Figure 12. Periarticular CD14^{hi} macrophages do not originate from circulating cells. (**A**) Scheme of adoptively transferring BMDMs from *Spry1* cKO mice (CD45.2) after recombinant murine CXCL10 stimulation to congenic wild-type CD45.1 mice by tail vein injection. (**B-D**) Macroscopic views of ears and paws (**B**); quantification of arthritis scores, and thickness of the digit, paw, and ear (n = 3) (**C**); representative H&E staining of paws (left scale bar, 500 µm; right scale bar, 250 µm) (**D**) from CD45.1 mice, four weeks after the first transfer of BMDMs or PBS (administered once a week for four weeks). (**E** and **F**) Flow cytometric plots (**E**) and frequencies (**F**) of CD45.1^{+} cells and CD45.2^{+} cells in the periarticular tissue from CD45.1 mice (four weeks after the first transfer of BMDMs or PBS) and *Spry1* cKO mice (CD45.2) (n = 3). Error bars indicate mean ± SEM. P values were determined using two-tailed unpaired Student's t-test (**C**) and One-way ANOVA (**F**).

Supplemental Table 1. Participant information

Participant	Gender	Age	BSA (%)	PASI	EASI	SCORAD	Sample type	Application
PsO patient-1	F	45	75	43.6	-	-	skin	IF and cell culture
PsO patient-2	M	36	65	36.5	-	-	skin	IF and cell culture
PsO patient-3	M	42	80	51.2	-	-	skin	IF and cell culture
PsO patient-4	F	51	60	31.5	-	-	skin	cell culture
PsO patient-5	M	21	50	25	-	-	serum	ELISA
PsO patient-6	M	47	55	27.9	-	-	serum	ELISA
PsO patient-7	F	55	65	42.4	-	-	serum	ELISA
PsO patient-8	F	29	48	30.6	-	-	serum	ELISA
PsO patient-9	M	28	25	11.3	-	-	serum	ELISA
PsO patient-10	M	48	52	28.2	-	-	serum	ELISA
PsO patient-11	F	60	64	36.8	-	-	serum	ELISA
PsO patient-12	F	24	32	15	-	-	serum	ELISA
PsO patient-13	M	37	75	40.8	-	-	serum	ELISA
PsO patient-14	M	46	55	25	-	-	serum	ELISA
PsO patient-15	F	40	60	28.7	-	-	serum	ELISA
PsO patient-16	F	47	28	16.6	-	-	serum	ELISA
PsO patient-17	F	27	45	26.3	-	-	serum	ELISA
PsO patient-18	M	35	55	36.9	-	-	serum	ELISA
PsO patient-19	M	28	30	19.2	-	-	serum	ELISA
AD patient-1	F	44	55	-	12.8	59.5	skin	IF
AD patient-2	F	39	60	-	28.5	67	skin	IF
AD patient-3	M	56	70	-	31.3	99.5	skin	IF
healthy control-1	F	29	-	-	-	-	skin	IF and cell culture
healthy control-2	F	48	-	-	-	-	skin	IF and cell culture
healthy control-3	M	40	-	-	-	-	skin	IF and cell culture
healthy control-4	M	36	-	-	-	-	skin	cell culture

healthy control-5	F	22	-	-	-	-	serum	ELISA
healthy control-6	F	39	-	-	-	-	serum	ELISA
healthy control-7	M	62	-	-	-	-	serum	ELISA
healthy control-8	M	53	-	-	-	-	serum	ELISA
healthy control-9	F	35	-	-	-	-	serum	ELISA
healthy control-10	M	43	-	-	-	-	serum	ELISA
healthy control-11	M	22	-	-	-	-	serum	ELISA
healthy control-12	F	53	-	-	-	-	serum	ELISA
healthy control-13	F	28	-	-	-	-	serum	ELISA
healthy control-14	M	52	-	-	-	-	serum	ELISA
healthy control-15	M	38	-	-	-	-	serum	ELISA
healthy control-16	M	26	-	-	-	-	serum	ELISA
healthy control-17	F	49	-	-	-	-	serum	ELISA
healthy control-18	M	34	-	-	-	-	serum	ELISA
healthy control-19	M	65	-	-	-	-	serum	ELISA

Abbreviations: PsO, psoriasis; AD, atopic dermatitis; BSA, body surface area; PASI, Psoriasis Area and Severity Index; ESAI, Eczema Area and Severity Index; SCORAD, SCORing Atopic Dermatitis; F, female; M, male; IF, immunofluorescence

Supplemental Table 2. Reagents and resources used in this study

Reagents and resources	Source	Identifier
**Reagent**		
Anti-Spry1 (Rabbit, Clone D9V6P)	Cell Signalling Technology	Cat# 13013; RRID: AB_2798087
Anti-Sprouty 1/SPRY1 (Mouse)	Abcam	Cat# ab56670; RRID: AB_945621
Anti-Ki67 (Rabbit)	Abcam	Cat# ab264429
Recombinant Anti-Cytokeratin 14 (Rabbit, Clone EPR17350)	Abcam	Cat# ab181595; RRID: AB_2811031
Recombinant Anti-CD4 (Rabbit, Clone EPR19514)	Abcam	Cat# ab183685; RRID: AB_2686917
Anti-Filaggrin (Rabbit)	Abcam	Cat# ab81468; RRID: AB_1640512
Anti-Loricrin Polyclonal (Rabbit)	Proteintech	Cat# 55439-1-AP; RRID: AB_2881332
Anti-Cathelicidin/CLP (Rabbit)	Abcam	Cat# ab180760
Recombinant Anti-Ly6g (Rabbit, Clone EPR22909-135)	Abcam	Cat# ab238132; RRID: AB_2923218
Recombinant Anti-CD14 (Rabbit, Clone EPR21847)	Abcam	Cat# ab221678; RRID: AB_2935854
Purified anti-mouse CD14 Antibody	Biolegend	Cat# 123302 ; RRID: AB_940592
Purified anti-mouse CD68 (Rat, Clone FA-11)	Biolegend	Cat# 137001; RRID: AB_2044003
CD68 Polyclonal antibody	Proteintech	Cat# 25747-1-AP ; RRID: AB_2721140
Purified anti-mouse CD45 (Rat, Clone 30-F11)	Biolegend	Cat# 103101; RRID: AB_312966
Purified anti-mouse F4/80 (Rat, Clone BM8)	Biolegend	Cat# 123101; RRID: AB_893504
Purified anti-mouse CD11c (Rat, Clone N418)	Biolegend	Cat# 117301; RRID: AB_313770
Anti-CXCL10/IP-10 Polyclonal (Rabbit)	Proteintech	Cat# 10937-1-AP; RRID: AB_2088002
Anti-CXCL10/IP-10 (Rabbit)	Bioss	Cat# bs-1502R; RRID: AB_10859700

Anti-Mouse CXCL10/IP-10/CRG-2 (Rat, Clone 134013)	R&D Systems	Cat# MAB466; RRID: AB_2292486
Anti-TLR4 (Mouse, Clone 3G9A4)	Proteintech	Cat# 66350-1-Ig; RRID: AB_2881730
Anti-TLR2 (Rabbit, Clone EPR20303)	Abcam	Cat# ab209217; RRID: AB_2814691
Anti-Phospho-JAK1 (Tyr1022, Tyr1023) (Rabbit)	Thermo Fisher Scientific	Cat# 44-422G; RRID: AB_2533648
Anti-JAK1 (Rabbit, Clone EPR349(N))	Abcam	Cat# ab133666; RRID: AB_2909402
Anti-Phospho-Jak2 (Tyr1007) (Rabbit, Clone D15E2)	Cell Signalling Technology	Cat# 4406; RRID: AB_10706164
Anti-Jak2 (Rabbit, Clone D2E12)	Cell Signalling Technology	Cat# 3230; RRID: AB_2128522
Anti-Phospho-Stat1 (Tyr701) (Rabbit, Clone D4A7)	Cell Signalling Technology	Cat# 7649; RRID: AB_10950970
Anti-STAT1 (phospho Y701) (Mouse, Clone M135)	Abcam	Cat# ab29045; RRID: AB_778096
Anti-Stat1 (Rabbit)	Cell Signalling Technology	Cat# 9172; RRID: AB_2198300
Anti-Stat1 (Mouse, Clone C-136)	Santa Cruz Biotechnology	Cat# sc-464; RRID: AB_675899
Anti-PI3 Kinase p110α (Rabbit, Clone C73F8)	Cell Signalling Technology	Cat# 4249; RRID: AB_2165248
Anti-Phospho-PI3 Kinase p85 (Tyr458)/p55 (Tyr199) (Rabbit)	Cell Signalling Technology	Cat# 4228; RRID: AB_659940
Anti-Phospho-Akt (Ser473) (Rabbit, Clone D9E)	Cell Signalling Technology	Cat# 4060; RRID:AB_2315049
Anti-Akt (Rabbit)	Cell Signalling Technology	Cat# 9272; RRID: AB_329827
Anti-Phospho-IKKα/β (Ser176/180) (Rabbit, Clone 16A6)	Cell Signalling Technology	Cat# 2697; RRID: AB_2079382
Anti-IKKβ (Rabbit, Clone D30C6)	Cell Signalling Technology	Cat# 8943; RRID: AB_11024092
Anti-Phospho-IκBα (Ser32) (Rabbit, Clone 14D4)	Cell Signalling Technology	Cat# 2859; RRID: AB_561111
Anti-IκBα (Mouse, Clone L35A5)	Cell Signalling Technology	Cat# 4814; RRID: AB_390781

Anti-Phospho-NF-κB p65 (Ser536) (Rabbit, Clone 93H1)	Cell Signalling Technology	Cat# 3033; RRID: AB_331284
Anti-NF-κB p65 (Rabbit, Clone D14E12)	Cell Signalling Technology	Cat# 8242; RRID: AB_10859369
Anti-β-Actin (Rabbit, Clone 13E5)	Cell Signalling Technology	Cat# 4970; RRID: AB_2223172
CXCL1 Monoclonal Antibody (48415)	Invitrogen	Cat# MA5-23745; RRID: AB_2609463
Atibuclimab (anti-CD14 antibody)	MedChemExpress	Cat# HY-P99008
InVivoMAb anti-mouse TNFα	BioXcell	Cat# BE0058; RRID: AB_1107764
InVivoMAb anti-mouse CXCR3 (CD183)	BioXcell	Cat# BE0249; RRID: AB_2687730
InVivoMAb rat IgG2a isotype control	BioXcell	Cat# BE0089; RRID: AB_1107769
InVivoMAb rat IgG1 isotype control	BioXcell	Cat# BE0088; RRID: AB_1107775
InVivoMAb polyclonal Armenian hamster IgG	BioXcell	Cat# BE0091; RRID: AB_1107773
Rabbit IgG	Proteintech	Cat# B900610
Mouse IgG	Proteintech	Cat# B900620
Anti-Rabbit IgG for IP, AlpSdAbs® VHH(HRP)	Alpvhhs	Cat# 025-100-005
Anti-Mouse IgG for IP, AlpSdAbs® VHH(HRP)	Alpvhhs	Cat# 001-100-005
HRP Conjugated AffiniPure Goat Anti-Rabbit IgG (H+L)	Boster	Cat# BA1054; RRID: AB_2734136
Brilliant Violet 510™ anti-mouse CD45 (Clone, 30-F11)	Biolegend	Cat# 103138; RRID: AB_2563061
PE anti-mouse F4/80 (Clone, BM8)	Biolegend	Cat# 123110; RRID: AB_893498
APC/Fire™ 750 anti-mouse CD86 (Clone, GL-1)	Biolegend	Cat# 105045; RRID: AB_2629769
Alexa Fluor® 700 anti-mouse CD68 (Clone, FA-11)	Biolegend	Cat# 137026; RRID: AB_2783097
APC/Cyanine7 anti-mouse Ly-6G (Clone, 1A8)	Biolegend	Cat# 127623 ; RRID:AB_10645331
BD Horizon™ BV421 anti-mouse CD11c (Clone, N418)	BD Biosciences	Cat# 565451; RRID: AB_2744278

PerCP-Cyanine5.5 anti-CD11b (Clone, M1/70)	eBioscience	Cat# 45-0112-80; RRID: AB_953560
FITC anti-CD14 (Clone, Sa2-8)	eBioscience	Cat# 11-0141-82; RRID: AB_464949
PE/Dazzle™ 594 anti-mouse CD163 (Clone, S15049I)	Biolegend	Cat# 155315; RRID: AB_2890708
PE/Dazzle™ 594 anti-mouse/human CD207 (Clone, 4C7)	Biolegend	Cat# 144211; RRID: AB_2876491
FITC anti-mouse CD3ε (Clone, 145-2C11)	Biolegend	Cat# 100306; RRID: AB_312670
Alexa Fluor® 700 anti-mouse CD4 (Clone, GK1.5)	Biolegend	Cat# 100430; RRID: AB_493699
PerCP/Cyanine5.5 anti-mouse CD8a (Clone, 53-6.7)	Biolegend	Cat# 100734; RRID: AB_2075239
PE anti-mouse CD8a Recombinant Antibody	Biolegend	Cat# 115007; RRID: AB_2783127
Brilliant Violet 421™ anti-mouse CD103 (Clone, 2E7)	Biolegend	Cat# 121422; RRID: AB_10900074
Brilliant Violet 650™ anti-mouse TNF-α (Clone, MP6-XT22)	Biolegend	Cat# 506333; RRID: AB_2562450
PE anti-IL-1 beta (Clone, NJTEN3)	eBioscience	Cat# 12-7114-82; RRID: AB_10732630
eFluor™ 660 anti-IL-23 p19 (Clone, 23dcdp)	eBioscience	Cat# 50-7823-42; RRID: AB_10597593
PE/Cyanine7 anti-mouse IL-12/IL-23 p40 (Clone, C15.6)	Biolegend	Cat# 505209; RRID: AB_2565644
PE/Dazzle™ 594 anti-mouse IFN-γ (Clone, XMG1.2)	Biolegend	Cat# 505845; RRID: AB_2563979
PE-Cyanine7 anti-IL-17A (Clone, eBio17B7)	eBioscience	Cat# 25-7177-82; RRID: AB_10732356
BD Horizon™ BV786 anti-mouse RORγt (Clone, Q31-378)	BD Biosciences	Cat# 564723; RRID: AB_2738916
PE anti-mouse CD183 (CXCR3) (Clone, CXCR3-173)	Biolegend	Cat# 126505; RRID: AB_1027656
APC anti-mouse CD183 (CXCR3) (Clone, S18001A)	Biolegend	Cat# 155905; RRID: AB_2814078
PE anti-Mouse CD183 (Clone, CXCR3-173)	BD Biosciences	Cat# 562152; RRID: AB_10897140

APC anti-mouse CXCR3 (Clone, 220803)	R&D Systems	Cat# FAB1685A
APC anti-CXCL10/IP10	Bioss	Cat# bs-1502R-APC; RRID: AB_10859700
APC anti-Sprouty1	Bioss	Cat# bs-11216R-APC
FITC Cytokeratin 14 (Clone, LL002)	Novus Biologicals	Cat# NBP2-34675F
PE/Cyanine7 anti-mouse CD115 (CSF-1R)	Biolegend	Cat# 135523; RRID: AB_2566459
PE anti-mouse CD265 (RANK)	Biolegend	Cat# 119805; RRID: AB_2205353
Brilliant Violet 421™ anti-mouse CD45.1	Biolegend	Cat# 110731; RRID: AB_10896425
PE anti-mouse CD45.2	Biolegend	Cat# 109807; RRID: AB_313444
TruStain FcX™ PLUS (anti-mouse CD16/32) (Clone, S17011E)	Biolegend	Cat# 156604; RRID: AB_2783137
Recombinant Murine IFN-γ	PeproTech	Cat# 315-05
Recombinant human IFN-γ	PeproTech	Cat# 300-02
Recombinant Murine IP-10 (CXCL10)	PeproTech	Cat# 250-16
Recombinant Murine M-CSF	PeproTech	Cat# 315-02
Recombinant Mouse CD14 (carrier-free)	BioLegend	Cat# 771904
Clodronate Liposomes	LIPOSOMA	Cat# CP-005-005
Resatorvid/TAK-242	MedChemExpress	Cat# HY-11109
Tofacitinib	MedChemExpress	Cat# HY-40354
Upadacitinib	MedChemExpress	Cat# HY-19569
Lipopolysaccharides	Sigma-Aldrich	Cat# L2630
Resiquimod (R848)	Sigma-Aldrich	Cat# SML0196
4-Hydroxytamoxifen	Sigma-Aldrich	Cat# H7904
Tamoxifen	MedChemExpress	Cat# HY-13757A
Dispase II	Gibco	Cat# 17105041
Trypsin-EDTA (0.25%)	Gibco	Cat# 25200056
Collagenase type IV	Sigma-Aldrich	Cat# C5138
DNase I	Roche	Cat# 11284932001
BD GolgiPlug™ Protein Transport Inhibitor (Containing Brefeldin A)	BD Biosciences	Cat# 555029
Cell Activation Cocktail (with Brefeldin A)	Biolegend	Cat# 423304
Zombie UV™ Fixable Viability Kit	Biolegend	Cat# 423107

Lipofectamine 3000 transfection reagent	Thermo Fisher Scientific	Cat# L3000015
Cell Available Lipid Nanoparticles (CALNP™) RNAi transfection reagent	D-Nano Therapeutics	Cat# DN001
ELISA MAX™ Deluxe Set Mouse TNF-α	Biolegend	Cat# 430904
ELISA MAX™ Deluxe Set Mouse IL-17A	Biolegend	Cat# 432504
ELISA MAX™ Deluxe Set Mouse IL-1β	Biolegend	Cat# 432604
ELISA MAX™ Deluxe Set Mouse IL-23	Biolegend	Cat# 433704
Mouse CXCL10/IP-10 ELISA Kit PicoKine™	Boster	Cat# EK0736
Human CXCL10/IP-10 ELISA Kit PicoKine™	Boster	Cat# EK0735
Mouse CRP Quick ELISA Kit	Boster	Cat# FEK0977
Mouse CXCL1/KC ELISA Kit	Boster	Cat# EK0723
Mouse RF-IgM (Rheumatoid Factor IgM) ELISA Kit	FineTest	Cat# EM4271
Mouse Luminex® Discovery Assay	R&D Systems	Cat# LXSAMSM
Modified Safranin O and Fast Green Stain Kit (For Bone)	Solarbio	Cat# G1371-5
Leukocyte Acid Phosphatase (TRAP) Kit	Sigma-Aldrich	Cat# 387A-1KT
BD Cytofix/Cytoperm™ Fixation and Permeabilization Solution	BD Biosciences	Cat# 554722
BD Perm/Wash™ Perm/Wash Buffer	BD Biosciences	Cat# 554723
Foxp3/Transcription Factor Staining Buffer Set	eBioscience	Cat# 00-5523-00
Annexin V-FITC Apoptosis Kit	Beyotime	Cat# C1062
TruSeq PE Cluster Kit v3-cBot-HS	Illumina	Cat# PE-401-3001
Chromium Single Cell 3′ GEM, Library & Gel Bead Kit v3	10x Genomics	Cat# PN-1000075
Protein A/G Magnetic Beads	MedChemExpress	Cat# HY-K0202
KGM™ Keratinocyte Growth Medium Bullet Kit™	Lonza	Cat# CC-3111
InVivoPure pH 7.0 Dilution Buffer	BioXcell	Cat# IP0070
UltraPure™ 1 M Tris-HCI Buffer, pH 7.5	Invitrogen	Cat# 15567027
**Mice**		
Mouse: C57BL/6	Shanghai SLAC Laboratory Animal Co., Ltd.	N/A
Mouse: *Spry1*$^{f/f}$	Cyagen Biosciences Inc.	N/A

Mouse: *K14*^{CreERT} (Tg (KRT14-cre/ERT)20Efu/J)	The Jackson Laboratory(8)	Strain NO.005107; RRID: IMSR_JAX:005107
Mouse: CD45.1 (C57BL/6Smoc-*Ptprc*$^{em1(K302E)Smoc}$)	Shanghai Model Organisms Center, Inc.	Cat# NO. NM-KI-210226
**Deposited data**		
RNA-seq data: skin samples of healthy controls and patients with psoriasis or atopic dermatitis	Choy et al.(9)	GSE153007
RNA-seq data: skin samples of healthy controls and psoriasis patients	Nair et al.(10)	GSE13355
RNA-seq data: epidermis samples of healthy controls and psoriasis patients	Qiu et al.(11)	GSE166388
RNA-seq data: skin samples of healthy controls and PsA patients	Deng et al.(12)	GSE205748
RNA-seq data: skin samples of patients with psoriasis, PsA, or ankylosing spondylitis	Johnsson et al.(13)	GSE186063
scRNA-seq data: synovial fluid of PsA patients	Abji et al.(14)	GSE161500
RNA-seq data: mouse epidermis	Cui et al.(15)	GSE232912
RNA-seq data: mouse dermis	This paper	GSE289141
scRNA-seq data: mouse periarticular tissue	This paper	GSE289142
**Software and algorithms**		
GraphPad Prism v9.0	GraphPad Software	https://www.graphpad.com/
FlowJo v10.4	Tree Star	https://www.flowjo.com/
CytExpert v2.6	Beckman Coulter	https://www.beckman.com/flow-cytometry/research-flow-cytometers/cytoflex/software
ImageJ	Schneider et al.(16)	https://imagej.net/ij/
qBase Plus	Bio-Rad	https://www.bio-rad.com/webroot/web/html/lsr/products/amplification_pcr/product_overlay/global/qbase-connect.html
HDOCK server	Yan et al.(17)	http://hdock.phys.hust.edu.cn/

NDP.view2	Hamamatsu	https://www.hamamatsu.com/us/en/product/life-science-and-medical-systems/digital-slide-scanner/U12388-01.html
LAS X	Leica	https://www.leica-microsystems.com/products/microscope-software/p/leica-las-x-ls/
Metascape	Zhou et al.(18)	https://metascape.org/
R v4.1.2	R Core Team	https://www.r-project.org/
Rstudio v1.4.1717	Rstudio Team	https://www.rstudio.com/
Cell Ranger v6.1.2	10x Genomics	https://10xgenomics.com/
Seurat v4.3.0.1	Satija et al.(4)	https://satijalab.org/seurat/

Supplemental Table 3. Primer sequences for real-time PCR

Species	Orientation	Gene	Sequence (5' to 3')
Mouse	F	*Actb*	TCATGAAGTGAGACGTTGAC
Mouse	R	*Actb*	CCTAGAAGCACTTGCGGTGCACGATG
Mouse	F	*Ccl20*	AACTGGGTGAAAAGGGCTGT
Mouse	R	*Ccl20*	GTCCAATTCCATCCCAAAAA
Mouse	F	*Cd14*	TTGAACCTCCGCAACGTGTCGT
Mouse	R	*Cd14*	CGCAGGAAAAGTTGAGCGAGTG
Mouse	F	*Cd86*	ACGTATTGGAAGGAGATTACAGCT
Mouse	R	*Cd86*	TCTGTCAGCGTTACTATCCCGC
Mouse	F	*Ctsk*	AGCAGAACGGAGGCATTGACTC
Mouse	R	*Ctsk*	CCCTCTGCATTTAGCTGCCTTTG
Mouse	F	*Cxcl10*	ATCATCCCTGCGAGCCTATCCT
Mouse	R	*Cxcl10*	GACCTTTTTTGGCTAAACGCTTTC
Mouse	F	*Fos*	GCGCAAAAGTCCTGTGTGTT
Mouse	R	*Fos*	CCCGGCTTTCCCCAAACTT
Mouse	F	*Il10*	CAGAGCCACATGCTCCTAGA
Mouse	R	*Il10*	TGTCCAGCTGGTCCTTTGTT
Mouse	F	*Il12*	ACGAGAGTTGCCTGGCTACTAG
Mouse	R	*Il12*	CCTCATAGATGCTACCAAGGCAC
Mouse	F	*Il17a*	CAGACTACCTCAACCGTTCCAC
Mouse	R	*Il17a*	TCCAGCTTTCCCTCCGCATTGA
Mouse	F	*Il18*	GACAGCCTGTGTTCGAGGATATG
Mouse	R	*Il18*	TGTTCTTACAGGAGAGGGTAGAC
Mouse	F	*Il1b*	CACAGCAGCACATCAACAAG
Mouse	R	*Il1b*	GTGCTCATGTCCTCATCCTG
Mouse	F	*Il22*	GCTTGAGGTGTCCAACTTCCAG
Mouse	R	*Il22*	ACTCCTCGGAACAGTTTCTCCC
Mouse	F	*Il23a*	CATGCTAGCCTGGAACGCACAT

Mouse	R	*Il23a*	ACTGGCTGTTGTCCTTGAGTCC
Mouse	F	*Il6*	ACCTGTCTATACCACTTC
Mouse	R	*Il6*	GCATCATCGTTGTTCATA
Mouse	F	*Mmp9*	GCTGACTACGATAAGGACGGCA
Mouse	R	*Mmp9*	TAGTGGTGCAGGCAGAGTAGGA
Mouse	F	*Nfatc1*	TAACTGTAGTGTTCTGCGGC
Mouse	R	*Nfatc1*	GCAGAGATTGGAGGCCTTGTG
Mouse	F	*Nos2*	GAGACAGGGAAGTCTGAAGCAC
Mouse	R	*Nos2*	CCAGCAGTAGTTGCTCCTCTTC
Mouse	F	*Tnf*	TGTAGCCCACGTCGTAGCAAA
Mouse	R	*Tnf*	CTGGCACCACTAGTTGGTTGT
Mouse	F	*Tnfrsf11a*	GGACAACGGAATCAGATGTGGTC
Mouse	R	*Tnfrsf11a*	CCACAGAGATGAAGAGGAGCAG
Mouse	F	*Tnfsf11*	GTGAAGACACACTACCTGACTCC
Mouse	R	*Tnfsf11*	GCCACATCCAACCATGAGCCTT
Mouse	F	*Acp5*	CCTCTGCAACTCTGGACTCTG
Mouse	R	*Acp5*	AATCCATCTTGGCGGTGGG
Human	F	*ACTB*	CACCATTGGCAATGAGCGGTTC
Human	R	*ACTB*	AGGTCTTTGCGGATGTCCACGT
Human	F	*CXCL10*	GGTGAGAAGAGATGTCTGAATCC
Human	R	*CXCL10*	GTCCATCCTTGGAAGCACTGCA

Supplemental Table 4. Key residues in the interaction between mouse CD14 and CXCL10, identified by molecular docking

CD14	CXCL10	Distance(Å)	Specific Interactions
Lys 306	Lys 66	2.2	Van Der Waals Force
Lys 306	Lys 62	0.8	Van Der Waals Force
Ser 304	Lys 66	2.7	Van Der Waals Force
Asn 302	Phe 68	3.6	Van Der Waals Force
Tyr 285	Lys 59	2.1	Van Der Waals Force
Tyr 285	Asn 63	1.5	Van Der Waals Force
Tyr 285	Lys 62	1.8	Van Der Waals Force
Ser 284	Asn 63	2.7	Van Der Waals Force
Ser 284	Lys 66	3.7	Van Der Waals Force
Asp 282	Lys 66	2.6	Van Der Waals Force
Asp 282	Asn 63	3.7	Van Der Waals Force
Val 280	Phe 68	3.7	Van Der Waals Force
Phe 264	Asn 63	1	Van Der Waals Force
Phe 264	Lys 59	1.5	Van Der Waals Force
Phe 264	Thr 60	3	Van Der Waals Force
Phe 264	Lys 62	3.8	Van Der Waals Force
Ser 263	Asn 63	2	Van Der Waals Force
Asn 261	Lys 66	2.9	Van Der Waals Force
Asn 261	Ala 67	2.6	Van Der Waals Force
Asn 261	Asn 63	3.5	Van Der Waals Force
Hid 239	Thr 60	3.2	Van Der Waals Force
Hid 239	Arg 20	3.8	Van Der Waals Force
Ser 238	Met 21	3.8	Van Der Waals Force
Asp 236	Met 21	2.8	Van Der Waals Force
Asn 212	Arg 20	2.1	Van Der Waals Force
Arg 211	Arg 20	1.6	Hydrogen Bond
Arg 211	Met 21	2.4	Van Der Waals Force
Arg 211	Arg 22	3.4	Van Der Waals Force
Asp 184	Arg 20	2.2	Salt Bridge
Ser 183	Arg 20	0.7	Van Der Waals Force
Asp 181	Arg 20	3.8	Van Der Waals Force
Asp 181	Arg 22	4	Van Der Waals Force
Asp 181	Met 21	3.1	Van Der Waals Force
Ser 157	Arg 22	1.4	Van Der Waals Force

Arg 131	Arg 22	2	Van Der Waals Force
Arg 131	Arg 20	2.9	Van Der Waals Force
Asn 129	Lys 46	1.5	Van Der Waals Force
Asn 129	Arg 22	2.5	Van Der Waals Force
Asn 129	Ala 23	2.6	Van Der Waals Force
Leu 128	Lys 46	3.6	Van Der Waals Force
Ile 127	Lys 46	1.7	Van Der Waals Force
Glu 105	Arg 22	2.8	Salt Bridge
Thr 103	Lys 46	2	Hydrogen Bond
Thr 103	Arg 22	3.3	Van Der Waals Force
Leu 102	Lys 46	2.6	Van Der Waals Force
Glu 101	Lys 46	1.6	Van Der Waals Force
Arg 78	Lys 47	0.5	Van Der Waals Force
Arg 78	Arg 22	3.5	Van Der Waals Force
Thr 76	Lys 47	1.9	Van Der Waals Force
Arg 74	Lys 46	2.3	Van Der Waals Force
Arg 74	Asn 48	3.2	Van Der Waals Force
Arg 74	Asp 49	1.9	Van Der Waals Force
Arg 74	Thr 44	2.9	Van Der Waals Force
Arg 74	Gly 25	2.9	Van Der Waals Force
Arg 74	Met 45	3.7	Van Der Waals Force
Arg 74	Lys 47	3.5	Van Der Waals Force
Glu 39	Asn 48	1.7	Van Der Waals Force
Glu 39	Lys 47	1.4	Hydrogen Bond and Salt Bridge
Val 38	Asn 48	3.8	Van Der Waals Force
Asp 37	Asn 48	1.8	Van Der Waals Force
Asp 37	Asp 49	2.6	Van Der Waals Force
Asp 37	Lys 47	4	Van Der Waals Force
Ser 14	Asn 48	2.2	Van Der Waals Force
Ser 14	Asp 49	3.8	Van Der Waals Force
Glu 13	Asp 49	2.3	Van Der Waals Force

References

1. Shi Z, et al. Targeting the CCR6/CCL20 Axis in Entheseal and Cutaneous Inflammation. *Arthritis Rheumatol.* 2021;73(12):2271-81.
2. Kabala PA, et al. Promotion of macrophage activation by Tie2 in the context of the inflamed synovia of rheumatoid arthritis and psoriatic arthritis patients. *Rheumatology (Oxford).* 2020;59(2):426-38.
3. Toda G, et al. Preparation and culture of bone marrow-derived macrophages from mice for functional analysis. *STAR Protoc.* 2021;2(1):100246.
4. Satija R, et al. Spatial reconstruction of single-cell gene expression data. *Nat Biotechnol.* 2015;33(5):495-502.
5. Yu G, et al. clusterProfiler: an R package for comparing biological themes among gene clusters. *OMICS.* 2012;16(5):284-7.
6. Hanzelmann S, et al. GSVA: gene set variation analysis for microarray and RNA-seq data. *BMC Bioinformatics.* 2013;14:7.
7. Smyth GK. Linear models and empirical bayes methods for assessing differential expression in microarray experiments. *Stat Appl Genet Mol Biol.* 2004;3:Article3.
8. Vasioukhin V, et al. The magical touch: genome targeting in epidermal stem cells induced by tamoxifen application to mouse skin. *Proc Natl Acad Sci U S A.* 1999;96(15):8551-6.
9. Choy DF, et al. Comparative transcriptomic analyses of atopic dermatitis and psoriasis reveal shared neutrophilic inflammation. *J Allergy Clin Immunol.* 2012;130(6):1335-43 e5.
10. Nair RP, et al. Genome-wide scan reveals association of psoriasis with IL-23 and NF-kappaB pathways. *Nat Genet.* 2009;41(2):199-204.

11. Qiu X, et al. ULK1 Inhibition as a Targeted Therapeutic Strategy for Psoriasis by Regulating Keratinocytes and Their Crosstalk With Neutrophils. *Front Immunol.* 2021;12:714274.

12. Deng J, et al. Multi-omics integration reveals a core network involved in host defence and hyperkeratinization in psoriasis. *Clin Transl Med.* 2022;12(12):e976.

13. Johnsson H, et al. Cutaneous lesions in psoriatic arthritis are enriched in chemokine transcriptomic pathways. *Arthritis Res Ther.* 2023;25(1):73.

14. Abji F, et al. Proteinase-Mediated Macrophage Signaling in Psoriatic Arthritis. *Front Immunol.* 2020;11:629726.

15. Cui YZ, et al. SPRY1 deficiency in keratinocytes induces follicular melanocyte stem cells migration to epidermis through p53/SCF/C-KIT signaling. *J Invest Dermatol.* 2024.

16. Schneider CA, et al. NIH Image to ImageJ: 25 years of image analysis. *Nat Methods.* 2012;9(7):671-5.

17. Yan Y, et al. HDOCK: a web server for protein-protein and protein-DNA/RNA docking based on a hybrid strategy. *Nucleic Acids Res.* 2017;45(W1):W365-W73.

18. Zhou Y, et al. Metascape provides a biologist-oriented resource for the analysis of systems-level datasets. *Nat Commun.* 2019;10(1):1523.
