# Supplementary material for: CXCL10 secreted by SPRY1-deficient epidermal keratinocytes fuels joint inflammation in psoriatic arthritis via CD14 signaling
Source: J Clin Invest. 2025 Jun 5;135(15):e186135. doi: 10.1172/JCI186135 (PMC12321389; doi:10.1172/JCI186135)

Full unedited blot for Figure 3

used in the Figures

Full unedited blot for Figure 3L

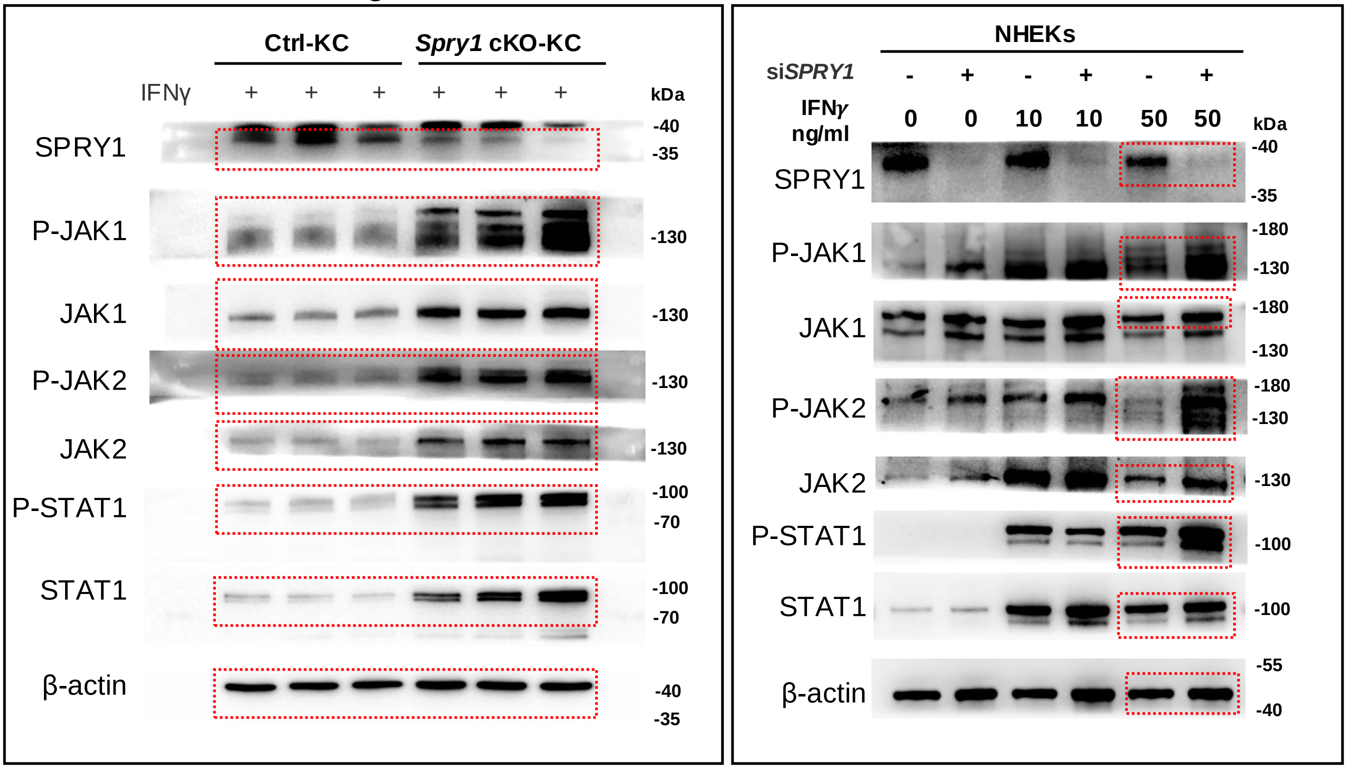

Merged with protein ladders

Merged with protein ladders

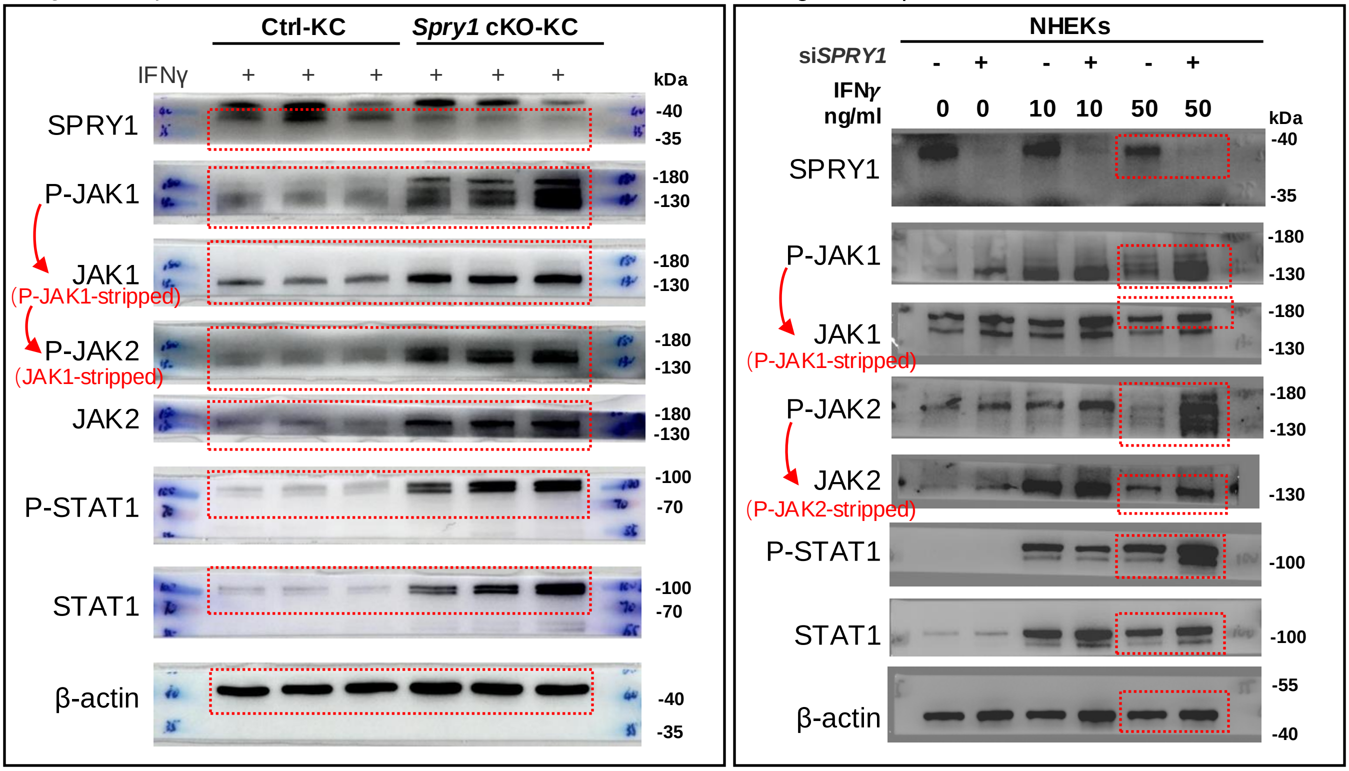

Full unedited blot for Figure 7

used in the Figures

Full unedited blot for Figure 7D

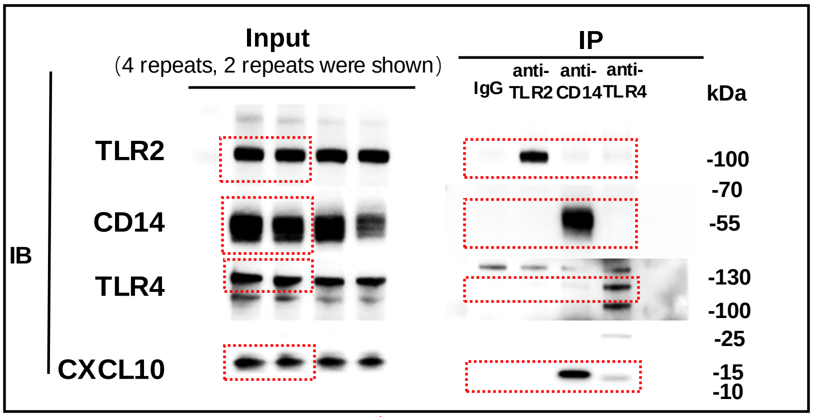

Full unedited blot for Figure 7E

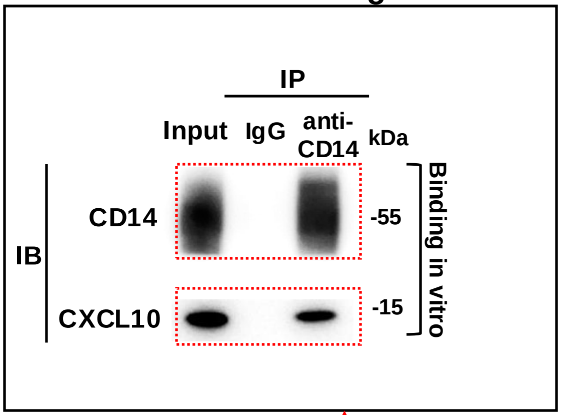

Merged with protein ladders

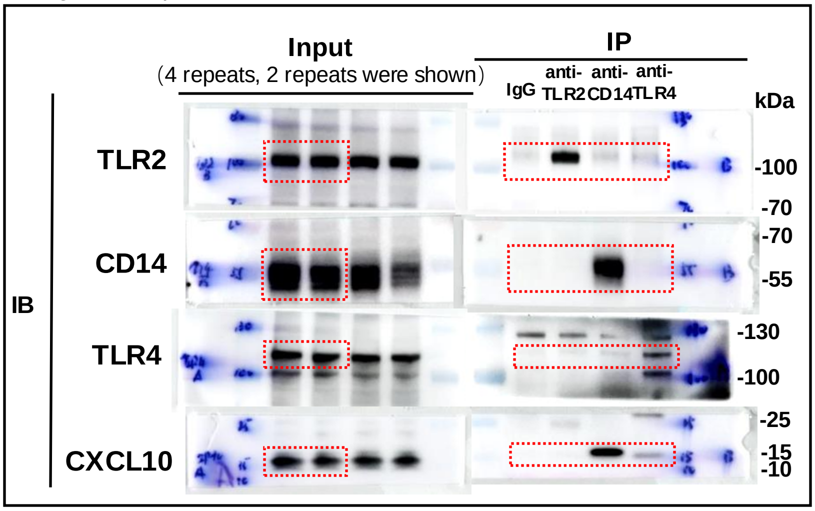

Merged with protein ladders

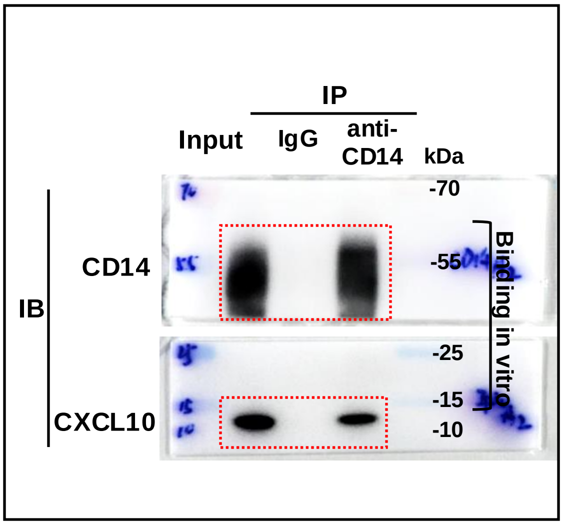

Full unedited blot for Figure 7H

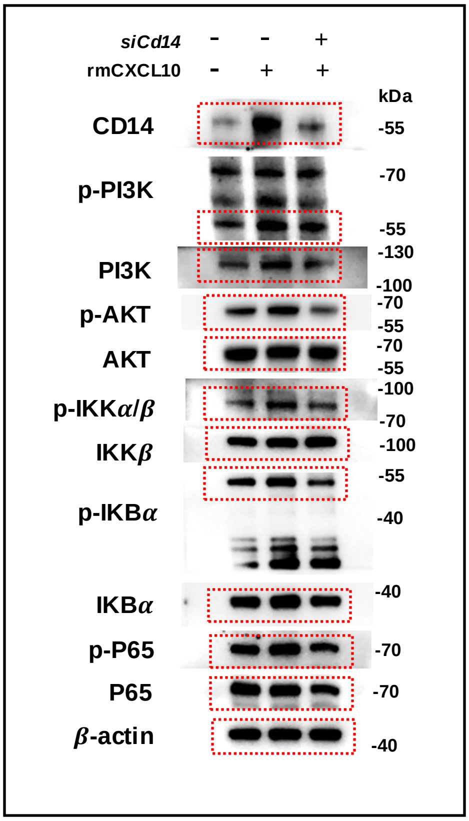

Merged with protein ladders

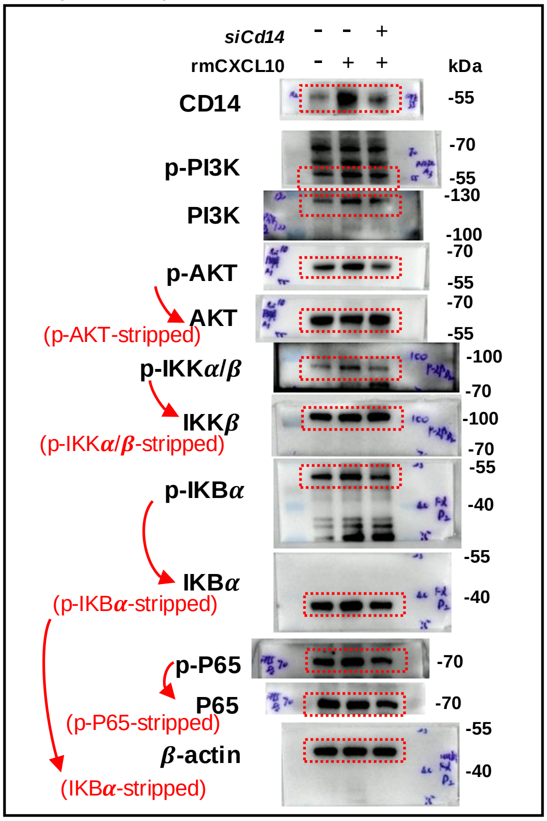

Full unedited blot for Supplemental Figure 1

used in the Figures

Full unedited blot for Supplemental Figure 1D

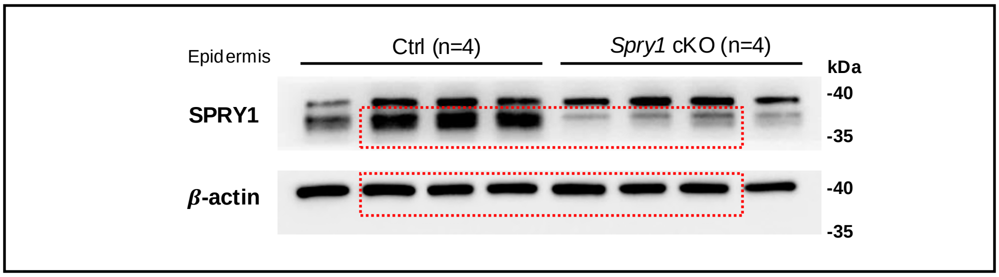

Merged with protein ladders

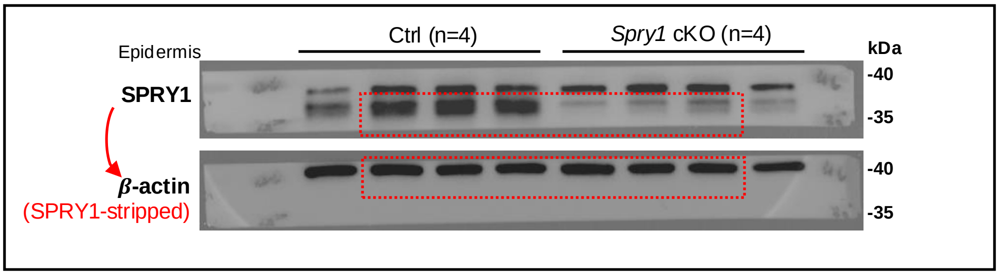

Full unedited blot for Supplemental Figure 4

used in the Figures

Full unedited blot for Supplemental Figure 4F

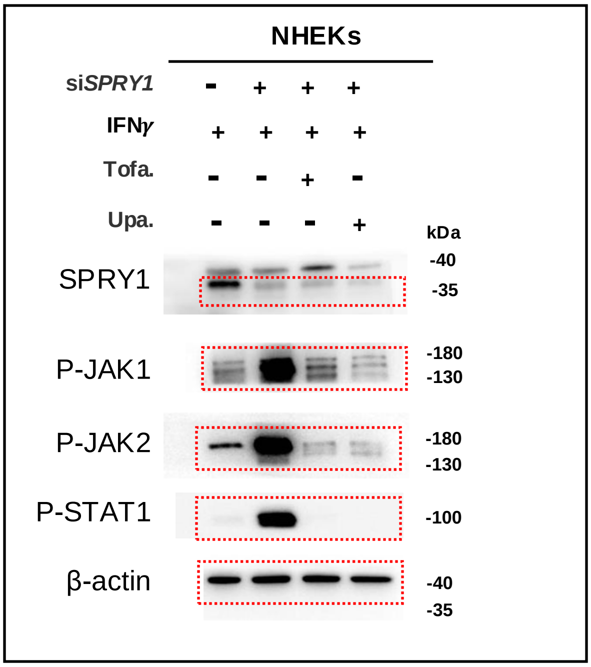

Merged with protein ladders

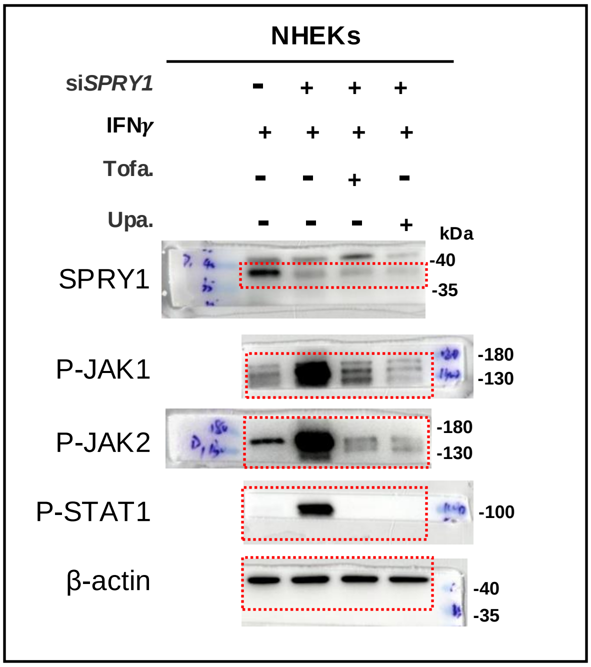

Supplement: Unedited blot and gel images [file jci-135-186135-s040.pdf]
